# Formaldehyde-Mediated Initial Carbon–Carbon Bond Formation in Zeolite-Catalyzed Methanol-to-Hydrocarbon Conversion

**DOI:** 10.1021/jacs.5c06141

**Published:** 2025-07-01

**Authors:** Wei Chen, Julia Sobalska, Wenqian Fu, Karolina A. Tarach, Massimo Bocus, Tiandi Tang, Kinga Góra-Marek, Veronique Van Speybroeck

**Affiliations:** † Center for Molecular Modeling, 26656 Ghent University, Technologiepark 46, 9052 Zwijnaarde, Belgium; ‡ Faculty of Chemistry, 37799 Jagiellonian University in Kraków, Gronostajowa 2, 30-387 Kraków, Poland; § Jiangsu Key Laboratory of Advanced Catalytic Materials and Technology, School of Petrochemical Engineering, 12412 Changzhou University, 213164 Changzhou, Jiangsu, P. R. China; ∥ Doctoral School of Exact and Natural Sciences, 37799 Jagiellonian University in Kraków, Łojasiewicza 11, 30-348 Kraków, Poland

## Abstract

Zeolite-catalyzed methanol-to-hydrocarbon conversion is a promising technology for the sustainable production of valuable hydrocarbon products. However, the mechanism behind the formation of the first carbon–carbon bond has been a subject of controversy for several decades. By comprehensive consideration of previous experimental phenomena and theoretical studies, a formaldehyde (HCHO)-based first carbon–carbon formation mechanism is proposed. Within the new mechanism, hydrated or methylated products of HCHO (methanediol, methyloxymethanol, and dimethyloxymethane) with much weaker C–H bond strengths replace methane in the traditional methane-HCHO mechanism, allowing energetically and kinetically favorable pathways to form the first C–C bond. The formed C–C bond products are further converted to ketene and olefins via the methylation-decarbonylation route. The plausibility of the newly proposed mechanism is confirmed by both theoretical calculations and experiments in various MTH zeolite catalysts. A key intermediate in this mechanism is glycolaldehyde, which was captured in situ by both mass spectrometry and Fourier transform infrared spectroscopy. The viability of the mechanism in different zeolites, as predicted theoretically, was also confirmed by gas chromatography. Not only does this new mechanism introduce an innovative pathway for the first C–C bond formation, but it also provides a comprehensive explanation of the specific role of HCHO in the early stage of the MTH process and associated reactions.

## Graphical abstract

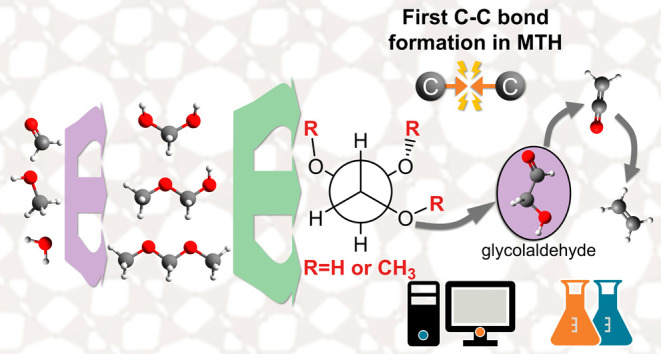

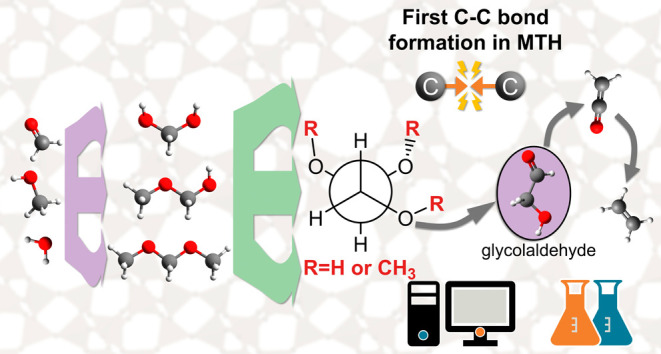

## 1 Introduction

The zeolite-catalyzed methanol-to-hydrocarbon (MTH) process is not only the most successful nonpetrochemical route for producing olefins and other chemicals^1–6^ but also an indispensable part of dual functional catalysts for C1 molecule conversion, such as syngas conversion,^7^ CO_2_ hydrogenation,^8^ etc. In the past few decades, enormous efforts have been devoted to elucidating the reaction mechanism, in both academia and industry. The MTH process can be divided into three periods, referred to as the induction, steady-state, and deactivation periods, as illustrated in Scheme 1A. Olefins are produced with high efficiency during the steady period via the so-called dual-cycle mechanism (i.e., olefin cycle and aromatic cycle),^9^ and shape selectivity to products is affected by the acidity,^10,11^ topology,^12–14^ morphology,^15,16^ and other factors of zeolites. Subsequently, the bulk intermediates (hydrocarbon pool species) confined in the voids of zeolites further grow toward coke precursors and impede the diffusion of reactants and products during the deactivation period.^17,18^ Despite intensive research, the mechanism of the first carbon–carbon bond formation during the induction period remains a topic of debate. The first olefin produced from these first C–C bond products serves as the fundamental feedstock for constructing hydrocarbon pool (HCP) species, therefore, it is of utmost importance to understand mechanistically the formation of the first C–C bonds and how they impact the further growth of the HCP species.

**Scheme 1:**
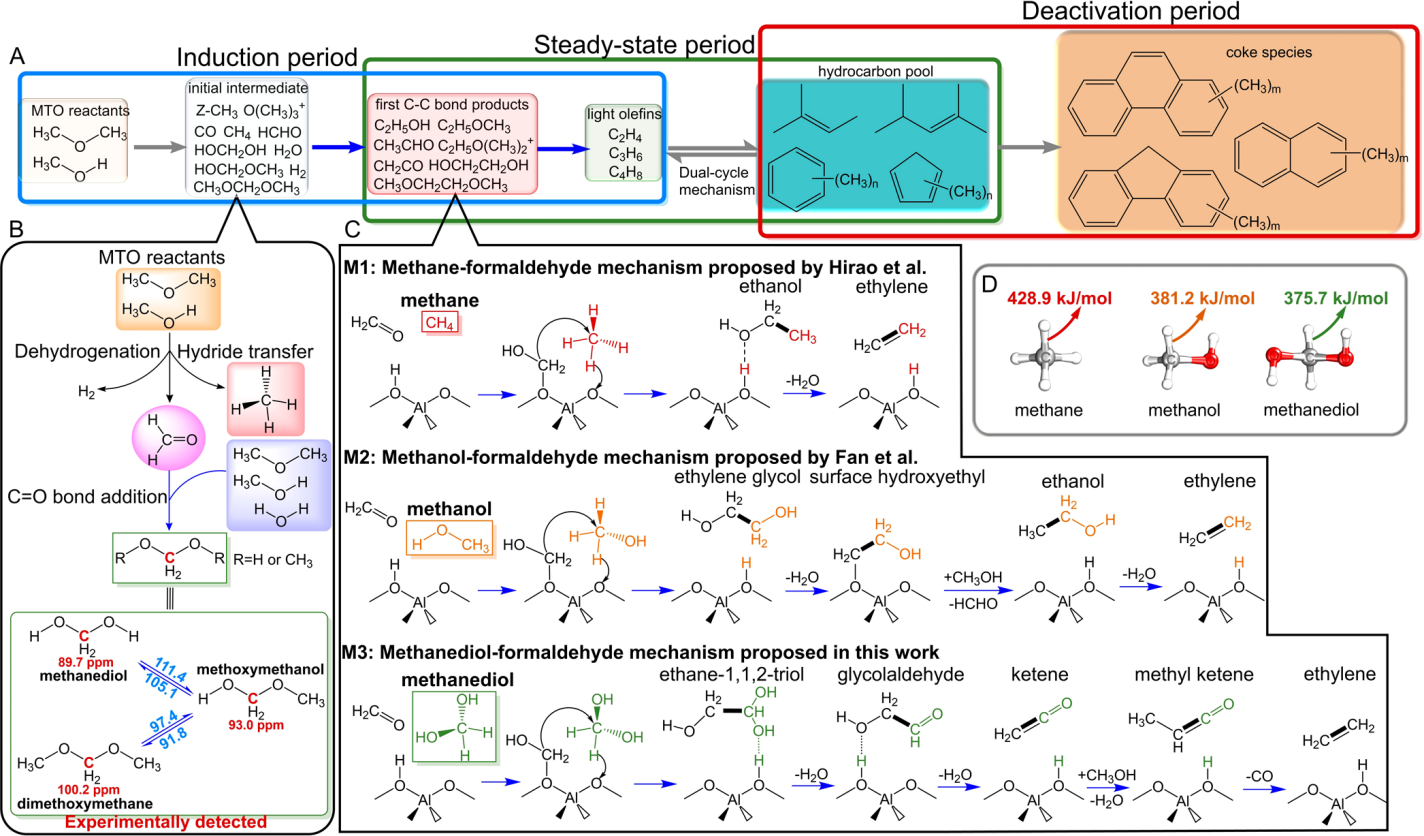
(A) Reaction Network and Different Periods of the MTH Process; (B) Route of HCHO Formation and Its Subsequent Addition to CH_2_(OR)_2_ (R=H or CH_3_) during the Induction Period of the MTH Process^a^; (C) First Carbon–Carbon Bond Formation Mechanism Based on HCHO in a Previous Work and This Work; (D) BDE of the Central C–H Bond in Methane, Methanol, and Methanediol, as the Representative Reactant in M1–M3 Mechanisms, Calculated by the PBE/def2-TZVP Method^b^

a The chemical shift of the central carbon atom in CH_2_(OR)_2_ was summarized and reported by Nastase et al.^53^ Values in blue are the calculated energy barriers of (de-)­methylation in SSZ-13 zeolite.
b All reactions indicated by the blue arrows in both (A) and (C) were calculated in this work.

The proposed mechanisms of the first C–C bond formation can be categorized into three types: carbene mechanism,^19,20^ cationic-based mechanisms (such as the oxonium-ylide mechanism^21^), and neutral-based mechanisms (such as the methane-formaldehyde (HCHO) mechanism,^22^ CO carbonylation mechanism,^23–25^ and direct mechanism^26–29^ ). The formation of carbene (:CH_2_) from the methyl group in the carbene mechanism is energetically highly demanding.^19,30^ Lesthaeghe et al.^31,32^ theoretically proved by means of DFT calculations the failure of both the oxonium-ylide and methane-formaldehyde mechanism caused by the instability of the ylide intermediates and the high energy barriers. Chu et al.^33,34^ developed a methane-HCHO mechanism based on the synergistic effect between BAS and LAS, reporting a lower barrier than the original one. Baltrusaitis et al.^30^ confirmed the high barriers of the oxonium-ylide mechanism via the Stevens rearrangement. The CO carbonylation mechanism was first proposed by Jackson and Bertsch^23^ in 1990 and further developed theoretically by Plessow and Studt.^24,25^ This mechanism is also known as the Koch-carbonylation route in the DME carbonylation to methyl acetate in zeolites and has been confirmed by some experimental proofs as the mechanism of the first C–C bond formation.^35–37^ Yet, the dehydrogenation of MTH reactants (methanol and DME) to HCHO^38^ and especially further dehydrogenation of HCHO to CO is energetically demanding by Bro̷nsted acid sites (BAS).^39^ On the other hand, HCHO, as the primary dehydrogenating product of methanol, is widely detected during the induction period of the MTH process (Scheme 1B). Further, numerous experimental results indicate the critical role of HCHO in the induction period of the MTH process owing to its strong correlation with olefin formation,^40–43^ but its precise role is still ambiguous. Aside from the induction period, it is accepted that HCHO also plays a role in deactivating the MTH catalyst.^44–48^ Indeed, HCHO can be formed via hydrogen transfer between olefins and methanol in the steady-state regime^40,49,50^ and actively contributes to the formation of coke species.^40,44–50^ In summary, HCHO plays a key role in the MTH process, nevertheless, its specific role to date has not been revealed, particularly in the induction period. Therefore, there is an urgent need to establish a reaction network for HCHO during the induction period of MTH in zeolites to gain a deeper understanding of both C1 chemistry and zeolite catalysis within MTH chemistry.

Due to the failure of the traditional methane-formaldehyde mechanism (M1) first proposed by Hirao et al.,^22^ Fan and co-workers^51,52^ suggested the use of methanol or dimethyl ether (DME) to replace methane for C–C bond formation (M2) and reported a lower barrier than the parent one (Scheme 1C). This modification is quite reasonable because the critical factor of the methane-HCHO mechanism is the activation of the inert C–H bond (with a bond dissociation enthalpy (BDE) of 428.9 kJ/mol) in methane. The BDEs of the C–H bond in methanol and DME significantly decrease to 381.2 and 382.0 kJ/mol by our calculations, which correlates with the lower barriers found by Fan and co-workers.^51,52^ However, the formation of ethylene from ethylene glycol still needs an energy-demanding hydride transfer process to produce ethylene.^51^ Given the decrease of the energy barrier from 149.6 kJ/mol in the M1 mechanism to 135.1 kJ/mol in the M2 mechanism as reported by Fan and co-workers,^51^ we question whether there is any other HCHO-based mechanism that gives low barriers to form the first C–C bond and subsequently allows one to produce ethylene with reasonable barriers. If such a mechanism existed, then it would entail a new mechanistic route to form the first ethylene in the MTH, which can afterward build up the HCP.

## 2 Results and Discussions

### 2.1 Indications for a New HCHO-Based Mechanism to Form the First C–C Bond

Regarding the reactions of HCHO in zeolites, the C=O double bond in HCHO can be involved in the nucleophilic addition reaction of water and methanol,^54^ resulting in the formation of CH_2_(OR)_2_ on the BAS of zeolite, that is, methanediol (MDO, HOCH_2_OH), methoxymethanol (MOM, HOCH_2_OCH_3_), and dimethoxymethane (DMM, CH_3_OCH_2_OCH_3_) as indicated in Scheme 1B. Note that there is an equilibrium between HCHO and MDO in aqueous HCHO (formalin solution),^55,56^ and both MDO and DMM were successfully detected by in situ solid-state NMR experiments during the initial MTH process.^34,53,57,58^ These experimental observations indicate the occurrence and potential contributions of MDO, MOM, and DMM during the induction period of the MTH process. Based on our calculations in SSZ-13, the formation of CH_2_(OR)_2_ via HCHO addition was found to be energetically favorable at low temperatures while becoming gradually more unfavorable with increasing temperature (Figures S1–S3). The HCHO addition with water to MDO catalyzed by BAS is a barrierless step, which presents an energy barrier of 170 kJ/mol in the gas phase according to Kaiser and co-workers.^59^ The addition products of HCHO can interconvert via (de)­methylation in zeolites with an energy barrier lower than 120 kJ/mol, as shown in Scheme 1B and Figure S4. The chemical form of these three compounds, CH_2_(OR)_2_, can be regarded as a decorated “methane” with two H atoms of CH_4_ replaced by the -OR group (R=H or CH_3_). The two -OR groups lead to reduced BDEs of the C–H bonds in CH_2_(OR)_2_ of 361.9 ∼ 375.7 kJ/mol (Scheme 1D and Table S1), suggesting a new mechanism for C–C bond formation involving HCHO, where instead of methane, the “decorated methane” molecules are involved. This newly proposed mechanism, hereafter referred to as the M3 mechanism (Scheme 1C), is expected to have much higher activity on the first C–C bond formation. After the first C–C bond formation, we propose that all M3 products (like ethane-1,1,2-triol as a geminal diol) may subsequently undergo a dehydration process to glycolaldehyde,^60^ which may further dehydrate to ketene. Ethylene can finally be formed by ketene methylation to methyl ketene and the decarbonylation of methyl ketene as proposed by Plessow and Studt.^24,25^ Therefore, it is highly likely that the first C–C bond can be formed via this new HCHO-based mechanism, as shown in Scheme 1C.

In what follows, we verify this newly proposed M3 mechanism both experimentally and theoretically. Density functional theory (DFT) calculations were first carried out to compare the reaction kinetics of the M1–M3 mechanisms in two chabazite frameworks. Subsequently, the influence of zeolite frameworks on the M3 mechanism was investigated in eight common MTH zeolite catalysts. Additionally, the further steps from M3 products to ethylene were confirmed to be plausible reaction routes characterized by sufficiently low free energy barriers. The theoretical calculations allow us to propose a set of plausible reaction pathways and intermediates, which are verified to occur via mass spectrometry, Fourier transform infrared spectroscopy, and gas chromatography. Herein, the multivariate curve resolution-alternating least-squares (MCR-ALS) algorithm analysis of the FT-IR spectrum helps us identify some critical intermediates in the M3 mechanism. In the MTH process using methanol or DME as the reactant, DMM as the reactant of the M3 mechanism is typically detected as an intermediate with a low concentration,^34,53,57,58^ and it is even more difficult to detect the intermediates formed from its conversion. Therefore, we designed experiments where DMM itself is used as the sole or cofeeding reactant to increase the emergence of some unstable and otherwise undetectable intermediates if the proposed M3 mechanism for the first C–C bond formation is possible.

### 2.2 First C–C Bond Formation Based on HCHO in Chabazites

During the MTH process, there is a dynamic equilibrium among the surface methyl species (SMS), DME, and methanol via dehydration.^61–63^ Herein, both SMS and BAS are considered active sites to react with HCHO and obtain two surface species (Int1A: Zeo-CH_2_OH; and Int1B: Zeo-CH_2_OCH_3_). Both Int1A and Int1B, regarded as chemically adsorbed HCHO, then couple with all six reactants (methane, methanol, DME, MDO, MOM, and DMM) to form the first C–C bond in the M1–M3 mechanisms, as shown in Figure 1.

**Figure 1:**
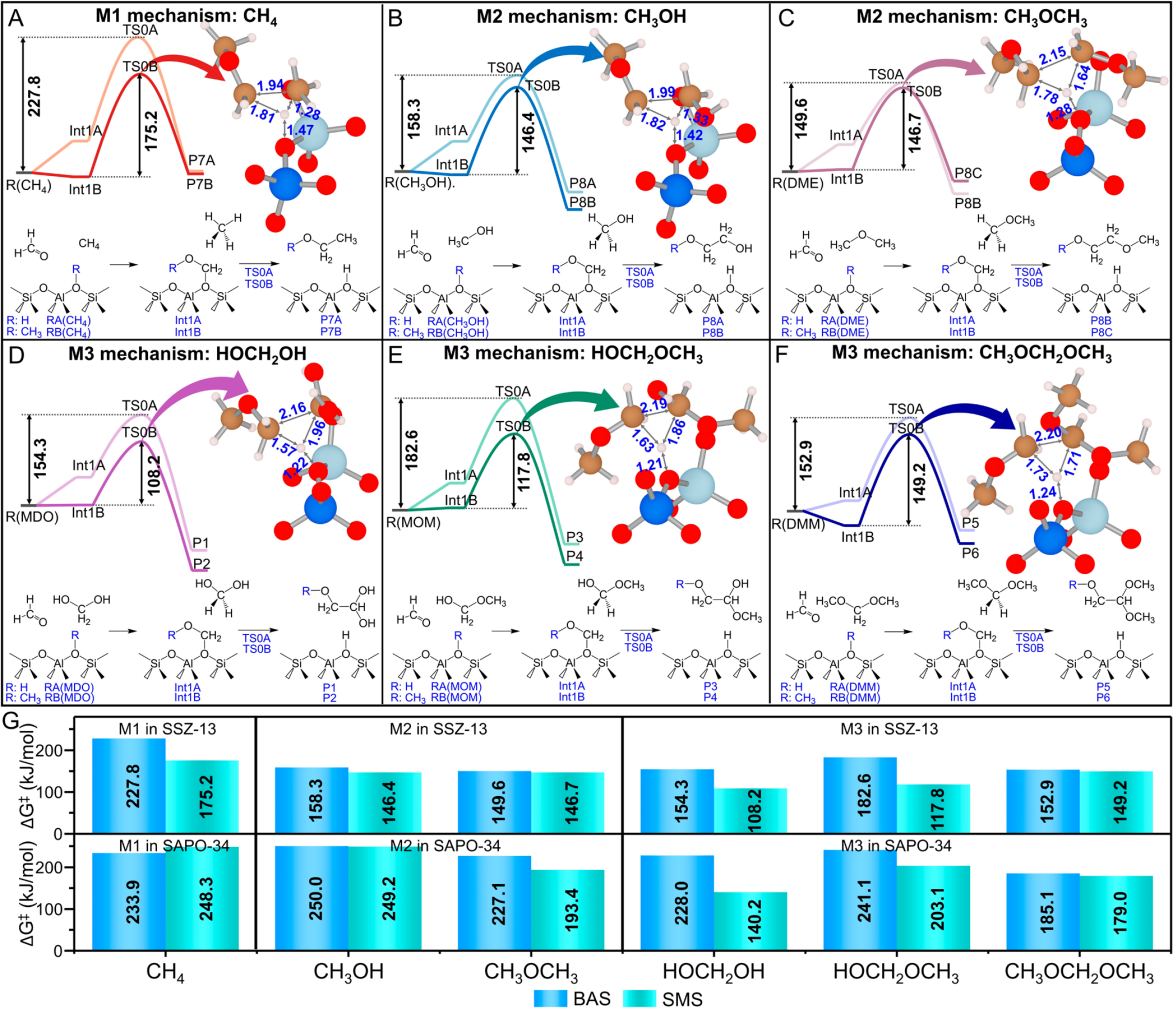
Free energy surfaces (673 K), reaction pathways, and transition state structure of the first C–C bond formation in the (A) M1 mechanism using CH_4_ as a reactant, (B, C) M2 mechanism using methanol and DME as reactants, and (D–F) M3 mechanism using MDO, MOM, and DMM as reactants. For each case, two paths are shown, starting from BAS (R: H) and SMS (R: CH_3_) as active sites, shown in lighter and darker colors, respectively. For the path using BAS as the active site, the letter A was added to the end of the label; likely, the RA­(MDO)–Int1A-TS0A-P1 path is the first C–C bond formation between MDO and HCHO on BAS via Int1A and TS0A. In contrast, B was added to the label when SMS was the active site. Distances are given in Å and free energy in kJ/mol. The double arrows represent the definition of the free energy barrier (Δ*G* ^‡^), defined as the free energy difference from R to TS0. Atom colors: carbon, orange; oxygen, red; hydrogen, white; silicon, blue; and aluminum, cyan. (G) Free energy barrier (Δ*G* ^‡^) comparison of the M1–M3 mechanisms using BAS or SMS as active sites in both SSZ-13 and SAPO-34.

To reveal the reactivities of M1–M3 reactants, free energy surfaces of the M1–M3 mechanisms in SSZ-13 were determined by periodic DFT calculations as shown in Figure 1A–F. The free energy barrier (Δ*G* ^‡^) was determined by the difference between the transition state and the lowest reactant state.^64^ Notably, SMS always has a higher catalytic activity than BAS, consistent with the earlier results found within ZSM-5 by Fan and co-workers.^52^ The lowest Δ*G* ^‡^ values of the M1–M3 mechanisms are 175.2 kJ/mol of methane, 146.7 kJ/mol of DME, and 108.2 kJ/mol of MDO. The significant decrease of Δ*G* ^‡^ from M1 to M2 and then to M3 suggests that the M3 mechanism is much more plausible than the traditional methane-HCHO mechanism and also more probable than the mechanisms starting from methanol or DME (M2). The decrease in the free energy barrier can be anticipated by the lower BDEs­(C–H), namely, 428.9 kJ/mol for CH_4_, CH_3_OR (381.2 and 382.0 kJ/mol), and CH_2_(OR)_2_ (361.9 ∼ 375.7 kJ/mol) (Table S1). The transition state for the C–C bond formation in the M1–M3 mechanism involves both a hydrogen transfer from the central carbon atom of the reactant to AlO_4_ and a C–H bond rupture leading to a triangle configuration of all TS0B. It is clear that the lengths of the triangles in TS0B are strongly dependent on different reactants. Furthermore, it can be seen that within the M3 reaction routes, the free energy barrier for the formation of P2 starting from MDO is much lower compared to the other products (P4 starting from MOM and P6 starting from DMM). This can be correlated to the stronger C–C bond in P2 (BDE­(C–C) = 328.9 kJ/mol) compared to the other products, namely, P4 (BDE­(C–C) = 318.8 kJ/mol) and P6 (BDE­(C–C) = 315.5 kJ/mol). Thus, BDE­(C–H) serves as the key descriptor for differentiating the catalytic activity of the M1–M3 mechanism, while BDE­(C–C) strongly correlates with the reactivity of three M3 reactants for the first C–C bond formation.

In addition to the inherent properties of reactants and products, the activity of the BAS catalytic reaction in zeolite is influenced by the acid strength and framework confinement.^17,65^ In terms of acid strength, SAPO-34 has the same CHA topology as SSZ-13. Still, their different elemental compositions result in a lower BAS strength in SAPO-34 as well as a different microscopic chemical environment.^66,67^ Herein, the Δ*G* ^‡^ of the first C–C bond formation in both SAPO-34 and SSZ-13 was compared for the M1–M3 mechanisms in Figure 1G. The catalytic activity of SAPO-34 is systematically much lower than that of SSZ-13, and the SMS also shows a higher activity than BAS, which is consistent with the findings in SSZ-13. These results indicate that both the acidic strength and internal surface polarity greatly affect catalytic activity. However, despite the lower reactivity observed within H-SAPO-34, the first C–C bond formation using MDO as a reactant is still likely to occur with a low Δ*G* ^‡^ of 140.2 kJ/mol compared to 248.3 kJ/mol for the parent methane-HCHO mechanism. Overall, the energetically unfavorable methane-HCHO mechanism seems to be plausible when considering three CH_2_(OR)_2_ molecules, i.e., methanediol (MDO), methoxymethanol (MOM), and dimethoxymethane (DMM), which are reactants in the newly proposed M3 reaction mechanism.

### 2.3 Influence of Different Zeolite Frameworks on the C–C Bond Formation

To verify the generality of the M3 mechanism to couple HCHO with CH_2_(OR)_2_, the proposed mechanism was investigated in different zeolite topologies, which are known for their high catalytic activity within the MTH process. To this end, we compare the catalytic activity of the M3 mechanism in five window-cage-like zeolites with different window sizes and cage sizes (SSZ-13 (CHA),^68^ SAPO-34 (CHA),^69^ SSZ-39 (AEI),^70^ RUB-50 (LEV),^14^ and ECR-10 (RHO)^71^) and three channel-like zeolites with different dimensions and channel sizes (ZSM-5 (MFI),^13^ ZSM-22 (TON),^72^ and β (BEA)^73^). In light of the much higher activity of SMS compared to the BAS for the M3 mechanism in SSZ-13 and SAPO-34, SMS was considered the sole active site to compare the Δ*G* ^‡^ in these eight zeolites. As displayed in Figure 2, HOCH_2_OH is systematically the most active reactant, with lower Δ*G* ^‡^ (104.7 ∼ 152.8 kJ/mol) compared to HOCH_2_OCH_3_ (119.2 ∼ 203.1 kJ/mol) and CH_3_OCH_2_OCH_3_ (146.0 ∼ 186.6 kJ/mol) in all zeolites. The broad range of Δ*G* ^‡^ indicates the great influence of different topologies on the M3 mechanism. Within SSZ-13, RUB-50, and β, the first C–C bond will more likely be formed via the M3 mechanism, as all Δ*G* ^‡^ values in these zeolites are lower than 153.2 kJ/mol. Notably, the low barriers in these three zeolites (Figure 2B,G,I) are not strictly related to the size of the zeolite channel/window: SSZ-13 and RUB-50 have the eight-ring (8R) window with the different sizes of 3.8 Å × 3.8 Å and 3.6 Å × 4.8 Å, respectively, whereas β has a much larger 12R channel (6.0 Å × 6.9 Å). For zeolites with low activity, ECR-10 has the same 8R window size as RUB-50 (3.6 Å × 4.8 Å) but leads to the lowest activity of HOCH_2_OH (Δ*G* ^‡^ = 152.8 kJ/mol in Figure 2H). SSZ-39 has the same size of 8R window as SSZ-13 but gives a much higher Δ*G* ^‡^ value (Figure 2B,F). Therefore, the reactivities of the M3 reactants are potentially influenced by the local microscopic chemical environments of different topologies, and the position of BAS with different local environments is expected to also influence the barrier. Moreover, the entropic effect was found to greatly contribute to the large scope of Δ*G* ^‡^ in different zeolites by the thermodynamical analysis based on harmonic oscillator approximation in Figures S5 and S6.

**Figure 2:**
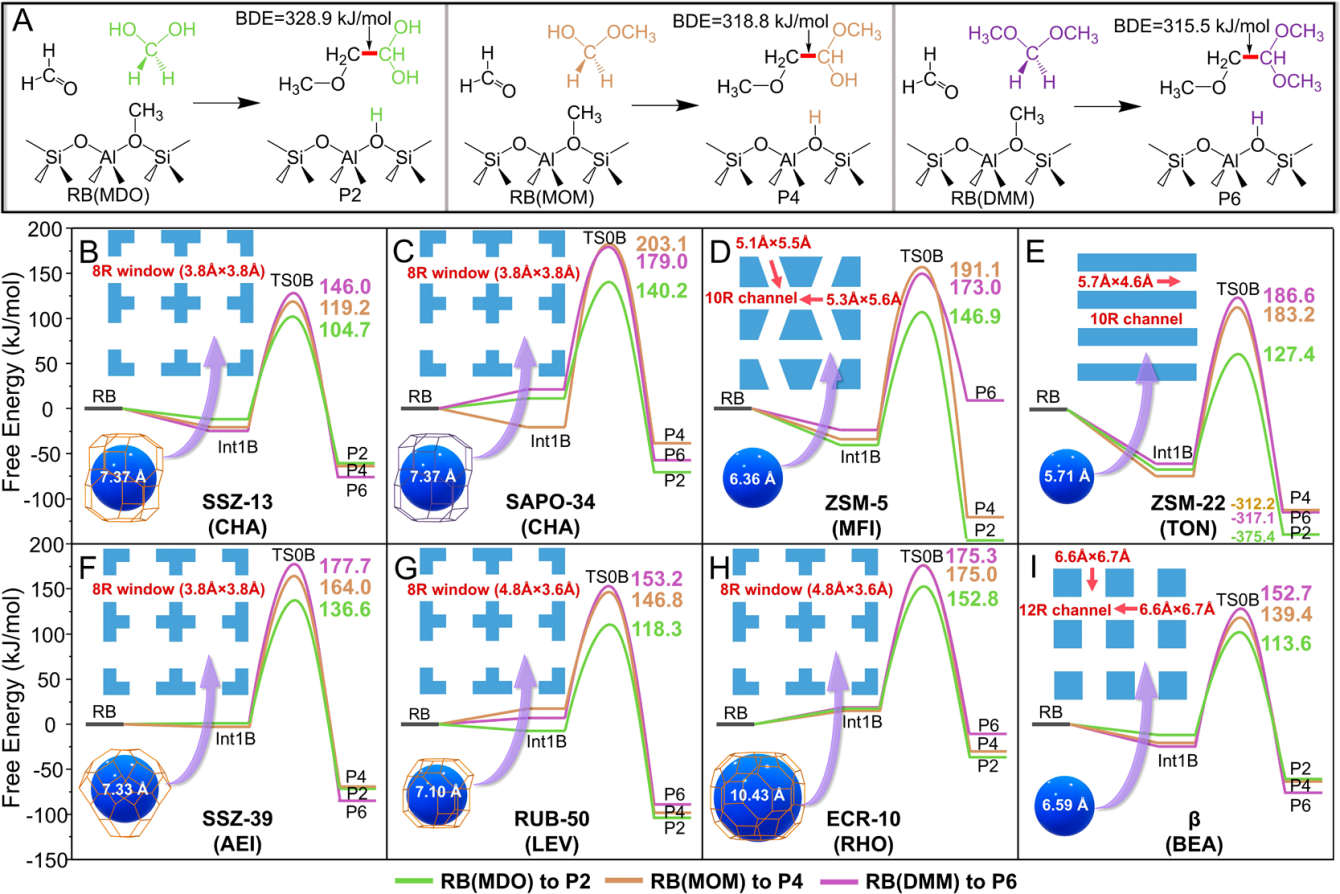
(A) Reaction pathways of the first C–C bond formation using MDO (left), MOM (medium), and DMM (right) as reactants following the M3 mechanism, and (B–I) free energy surfaces of reaction pathways shown in Panel A in different zeolites. All free energy barriers are defined by the free energy difference between TS0B and RB/Int1B. The values of spheres in the bottom-left corner are the maximum diameter of this zeolite topology that can be included.

In contrast to rigid methane, methanol, and DME in M1–M2 mechanisms, MDO, MOM, and DMM are reactants with much higher structural flexibility, which may be beneficial to fit the different confinement environments contributing to a lower Δ*G* ^‡^ of the first C–C bond formation in the M3 mechanism. To understand the structural flexibility of the reactants in M3, the rotation energy surfaces of three M3 reactants in both the gas phase and SSZ-13 zeolite were calculated (Figures S7–S10). The free energy barriers connecting the two isomers in the gas phase at 673 K are 31.6 kJ/mol for DMM, 34.3 kJ/mol for MOM, and 12.2 kJ/mol for MDO, demonstrating their flexibility even in the gas phase. Within SSZ-13, these barriers of DMM and MOM further decrease to 23.2 and 17.7 kJ/mol in SSZ-13, as the hydrogen-bonding interaction with BAS and van der Waals interaction with the framework promote their rotations in zeolites, as shown in Figure S10. Therefore, the structural variability of all M3 reactants facilitates their adjustment to fit the diverse pore architectures of zeolites. Furthermore, the bigger molecular size of M3 reactants also allows a better interaction with the zeolite framework via dispersion interaction, as displayed in Figure S11, contributing to the relatively low free energy barrier of the M3 mechanism.

Overall, MDO, MOM, and DMM, formed as the hydrated and methylated products of HCHO, are highly active reactants that couple with HCHO to form the first C–C bond more favorably than methane, methanol, and DME. The M3 mechanism of the first C–C bond formation is feasible in different zeolites. SSZ-13, RUB-50, and β showed much higher activities in forming the first C–C bond compared to the other MTH zeolite catalysts. The potential interconversion of the three M3 reactants by (de)­methylation also allows the adaptability of the M3 mechanism to the zeolite environment. Furthermore, the structural flexibility of the M3 reactants in zeolites enables them to better fit the pore architecture, leading to their high reactivity. Given the high possibility that the M3 mechanism is responsible for forming the first C–C bond, a reasonable and favorable route is also required to form olefins as feedstock to construct the HCP during the induction period of the MTH process starting from the products of the M3 route. Within the next section, ethylene production routes will be investigated starting from the M3 products, within the SSZ-13 catalyst, as this showed the best catalytic activity.

### 2.4 Production of the First Ethylene

After the C–C bond formation takes place via the M3 route, six products are formed (P1–P6 as shown in Figure 3A); however, the remaining −CH_2_–CH– unit in the M3 products cannot be directly converted to olefins by a dehydration or demethylation process. Herein, we propose subsequent routes to form the first ethylene in the induction period of the MTH process. Six M3 products can be divided into geminal diols (P1 and P2), hemiketals (P3 and P4), and geminal diethers (P5 and P6). On the premises of three rotatable -OR groups in P1–P6, these products have a much higher structural flexibility than the reactants, and the rotating −OR groups lead to at least four isomers, which are close in energy in the gas phase (Figure S12). The flexibility of the different products allows their self-accommodation to fit the local structure of the zeolite internal surface.

**Figure 3:**
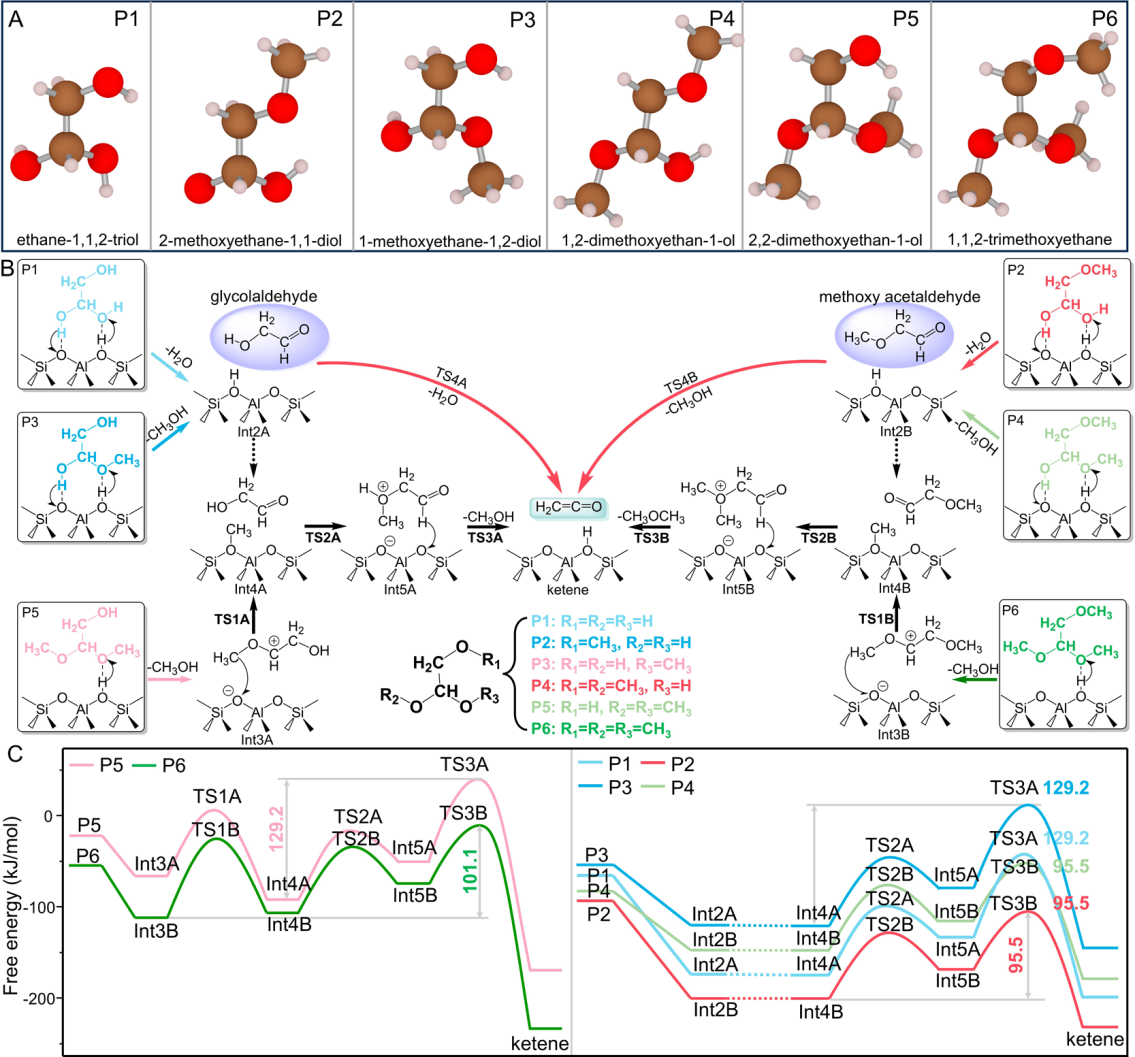
(A) Structures and nomenclature for the most stable conformers of the M3 products. (B) Reaction pathways for the conversion of M3 products to ketene. (C) Free energy surfaces (673 K) of all M3 products to ketene in SSZ-13, and values in different colors are (Δ*G* ^‡^) from P1–P6 to ketene, determined by the maximum free energy span.

Following a reaction equilibrium between aldehydes and diols in solution,^74,75^ two -OR groups on the terminal C atom of all six products can undergo an elimination reaction to release water or methanol as shown in Figure 3B, leading to Int2 and Int3. We found that the BAS in the zeolites catalyze this dehydration or demethylation of P1–P6 to form Int2 or Int3 with an energy barrier smaller than 50 kJ/mol (Figure S13). Herein, both Int2 and Int3 have two forms, Int2A is glycolaldehyde from P1 and P3, Int2B is methoxy acetaldehyde from P2 and P4, and Int3A and Int3B are two cationic species from P5 and P6, as displayed in Figure 3B. Note that the demethylation of two cations (Int3A and Int3B) via TS1A and TS1B will form glycolaldehyde and methoxy acetaldehyde with SMS, that is, Int4A and Int4B. Subsequently, the SMS of Int4 can transfer to the −OH group of glycolaldehyde and the -OCH_3_ group of methoxy acetaldehyde via TS2A and TS2B with the formation of Int5A and Int5B. Finally, ketene will be produced by a hydrogen transfer process based on Int5A and Int5B via TS3A and TS3B. In these routes, ketene will be formed via P5/P6–Int3–Int4–Int5–ketene with Δ*G* ^‡^ values of 129.2 and 101.1 kJ/mol (Figure 3C; all transition state structures are shown in Figure S14).

Alternatively, ketene can also be formed directly starting from P1 ∼ P4 via Int2A and Int2B, leading to glycolaldehyde and methoxy acetaldehyde adsorbed on BAS, which then produce ketene by a synergistic hydrogen transfer process (Figure 3B). Going through transition states (TS4A or TS4B), this route is kinetically less favorable, with free energy barriers Δ*G* ^‡^ of 171.8 and 189.1 kJ/mol (Figure S15). The higher Δ*G* ^‡^ of the Int2–ketene route on BAS than that of Int4–Int5–ketene on SMS shows that SMS is a more active site to convert both glycolaldehyde and methoxy acetaldehyde to ketene compared to BAS. Therefore, when Int2A or Int2B is formed directly from P1/P2 or P4/P5, glycolaldehyde and methoxy acetaldehyde should preferably migrate to SMS to further convert toward ketene via the Int2A–Int4A–ketene or Int2B–Int4B–ketene characterized by free energy barriers of 95.5 and 129.2 kJ/mol, respectively, as shown in Figure 3C. Additionally, the interconversion of P1–P6 via methylation was also considered, but it was found to be energetically unfavorable because of the high barriers (Figure S16). Summarizing, the conversion of P1 ∼ P6 to ketene via P1 ∼ P4–Int2–Int4–Int5–ketene or P5/P6–Int3–Int4–Int5–ketene is the preferred route, and glycolaldehyde or methoxy acetaldehyde serves as critical intermediates.

Once ketene is formed, light olefins (ethylene and propylene) can be produced by the methylation of ketene to (di)­methyl ketene and the following decarbonylation of (di)­methyl ketene,^24,25,76^ and decarbonylation is the rate-determining step with the Δ*G* ^‡^ of 137.6 kJ/mol from ketene to ethylene. Assembling all previous steps, we propose a complete reaction pathway and associated free energy surface for the formation of the first C–C bond and subsequent ethylene production according to the newly proposed M3 mechanism, as shown in Figures 4 and S17–S19. All Gibbs free energy values of the proposed reactions are listed in Tables S2–S4. Generally, the following reaction stages occur in the newly proposed mechanism, that is, C–C bond coupling with Δ*G* ^‡^ of 108.2 ∼ 182.6 kJ/mol, ketene production with Δ*G* ^‡^ of 95.5 ∼ 139.4 kJ/mol, and ethylene production with Δ*G* ^‡^ of 137.6 kJ/mol. The maximum Δ*G* ^‡^ of the optimal M3 route is 137.6 kJ/mol for both MDO and MOM (Figures S17–S18) and 149.2 kJ/mol for DMM (Figures 4 and S19). These low barriers indicate that the proposed M3 mechanism has a high probability of contributing to ethylene production during the induction period of the MTH process.

**Figure 4:**
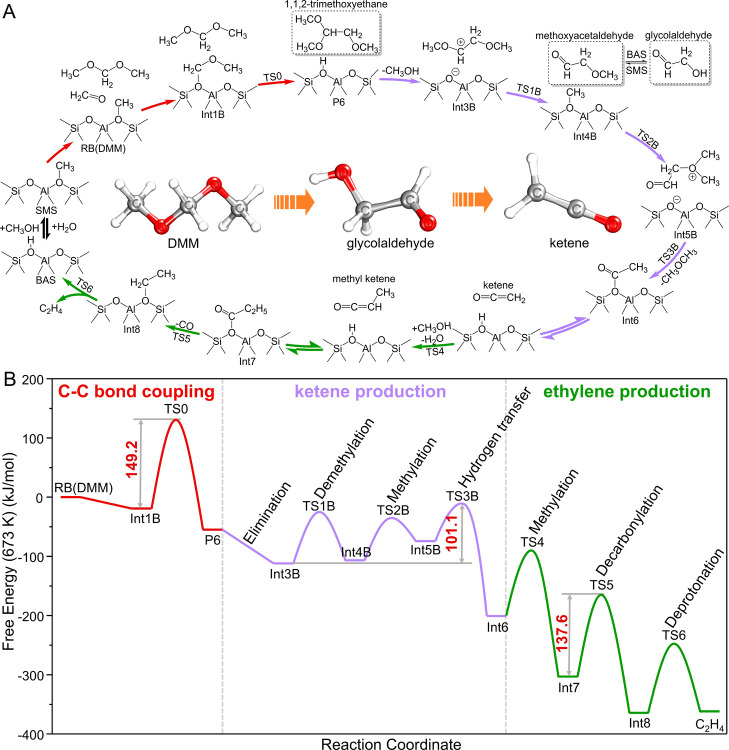
Optimal route of the M3 mechanism to ethylene in SSZ-13. (A) Reaction network and (B) free energy surfaces of DMM to ethylene catalyzed by SMS in the SSZ-13 zeolite at 673 K.

To comprehensively compare the contribution of three HCHO-based mechanisms for ethylene production, the complete pathways and free energy surfaces of the M1 and M2 mechanisms were also calculated, as shown in Figures S20–S22 and listed in Tables S5–S8. Ethylene can be directly formed by the dehydration of the M1 products (P7A: C_2_H_5_OH; P7B: C_2_H_5_OCH_3_), in contrast to the M2 products (P8A: HOCH_2_CH_2_OH; P8B: CH_3_OCH_2_CH_2_OH; P8C: CH_3_OCH_2_CH_2_OCH_3_). Based on the reported route by Fan and co-workers,^51,52^ M2 products will first dehydrate to two surface species (Int9A: Zeo-CH_2_CH_2_OH or Int9B: Zeo-CH_2_CH_2_OCH_3_ in Figure S21A); hydride transfer between two surface species and methanol/DME leads to the formation of C_2_H_5_OH and C_2_H_5_OCH_3_ as the substrate of ethylene production. Both the M1 and M2 mechanisms have barriers that are too high to substantially contribute to the formation of the first C–C bond. In the case of M2, a high barrier of hydride transfer of 179.8 ∼ 183.0 kJ/mol occurs, whereas in the M1 mechanism, high barriers are associated with the first C–C bond formation of 175.2 and 227.8 kJ/mol. Additionally, we calculated the Koch-carbonylation-based mechanism for ketene formation, shown in Figure S23, and compared it with the M3 mechanism (Table S9). The M3 mechanism is shown to be substantially more favorable than the carbonylation mechanism. (A more extensive discussion of the results is reported in Section 14 of the Supporting Information.)

Summarizing from our theoretical calculations, the M3 mechanism is an energetically more favorable HCHO-based route to produce the first ethylene in the construction of the HCP in the MTH process. Furthermore, some stable intermediates in the M3 mechanism, like 1,1,2-trimethoxyethane, methoxy acetaldehyde, and glycolaldehyde (Figure 4A), were identified, which may be used to experimentally confirm the proposed M3 mechanism.

### 2.5 Experimental Validations

Mass spectrometry, in situ time-resolved FT-IR spectroscopy, and gas chromatography were employed to capture the reaction intermediates of the MTH process, recognize the different activities of reactants in the M1–M3 mechanism, and compare the MTH catalytic activity of different zeolites. Methanol, DME, and DMM were used as reactants of the MTH process to examine their conversions in the theoretically optimal SSZ-13 zeolite. The species adsorbed were monitored by time-resolved FT-IR spectroscopy. The MCR-ALS analysis of FT-IR spectra^77,78^ can help identify the overlapping bands of different species that were ignored before.^79^ At the same time, products released from the catalyst surface were collected by mass spectrometry over the temperature range of 323–773 K, as displayed in Figure 5.

**Figure 5:**
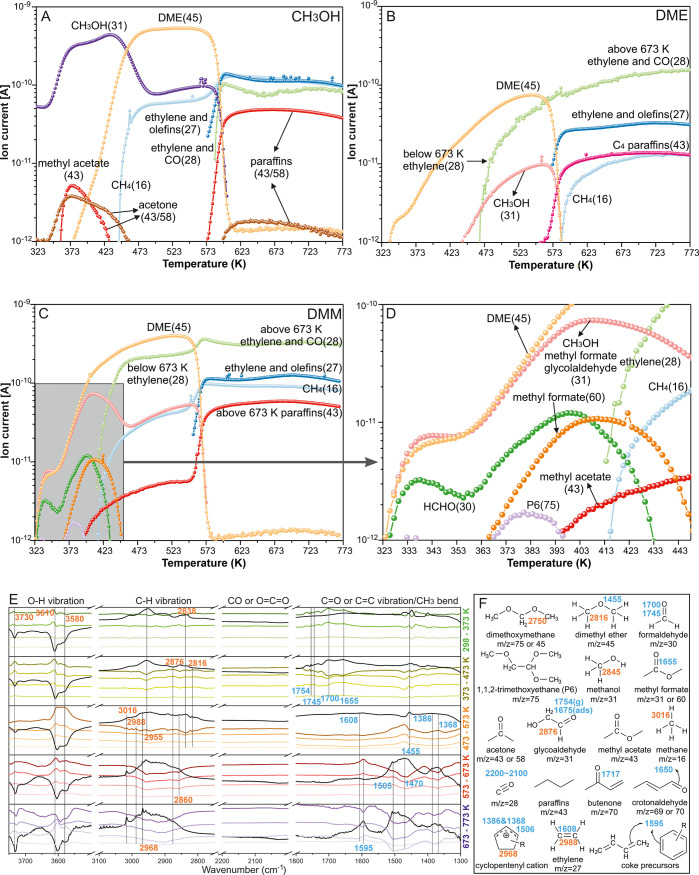
MS analysis of products using (A) CH_3_OH, (B) DME, and (C, D) DMM as reactants in H-SSZ-13 in the temperature range of 323–773 K, where D gives a more detailed view in the temperature range of 323–445 K. (E) FT-IR spectra of DMM as a reactant in H-SSZ-13 at 298–373, 373–473, 473–573, 573–673, and 673–773 K. For each temperature range, the first spectrum was taken as the reference one (marked with a black color), and this spectrum was subtracted from others in the same temperature range to follow the transformation of the reaction products. Thus, the minima in the spectra are attributed to the reagents consumed in a given temperature range, whereas the maxima are attributed to the newly formed species. (F) Assignments of wavenumbers (C–O and C–C vibrations, blue; O–H andC–H vibration, orange) and *m*/*z* ratios.

First, the reactivities of the respective reactants in the MTH process can be confirmed by the temperatures observed for the first ethylene (*m*/*z* = 28), that is, methanol (585 K) < DME (467 K) < DMM (413 K). This confirms that DMM shows a higher reactivity than both methanol and DME. As a M3 reactant, DMM will rapidly decompose into DME and HCHO at very low temperatures, and some HCHO may be further dehydrogenated to CO by Lewis acid sites such as extra-framework aluminum species, which could be present in the catalyst.^35,42^ Subsequently, the first C–C bond product (P6:1,1,2-trimethoxyethane) by DMM and HCHO in the M3 mechanism was detected at *m*/*z* = 75 (Figure 5D). Glycolaldehyde, as the critical intermediate in producing ketene, was observed by the relative intensity of *m*/*z* = 60 and 31, and methyl formate as the disproportionation product of DMM^80^ also contributes to the intensity of *m*/*z* = 60 and 31 and about 50% for each compound.

More importantly, the correlation between concentration changes of different components further confirms the mechanism we proposed. The consumption of HCHO below 373 K with the formation of 1,1,2-trimethoxyethane (P6 in Figure 3A) above 373 K indicates the first C–C bond formation between DMM and HCHO to 1,1,2-trimethoxyethane as a solid proof of the M3 mechanism in Figure 4A. Moreover, the occurrence of methyl acetate (*m*/*z* = 43, as the most stable addition product between ketene and methanol)^81^ accompanies the consumption of glycolaldehyde and 1,1,2-trimethoxyethane. This phenomenon further validates our proposed route of ketene formation in Figure 3, i.e., DMM–1,1,2-trimethoxyethane–glycolaldehyde–ketene, where methyl acetate will be rapidly formed by the addition of ketene with methanol.

Regarding DME as a reactant, no HCHO and its related intermediates were detected. When methanol was used as the reactant, acetone (*m*/*z* = 43 or 58) and methyl acetate (*m*/*z* = 43) were detected below 473 K (Figure 5A). Herein, acetone will be produced by the self-condensation of acetic acid, and isobutene will be formed by aldolization reaction and cracking of two acetones (Figure S24).^35,82,83^ Bhan and co-workers^84^ found the critical role of HCHO hydrolysis to MDO (M3 reactant) in the MTH reaction with cofeeding water, but the low HCHO concentration in our CH_3_OH/H_2_O = 1 experiments (Figures S25 and S26) still limits the direct detection of M3 intermediates. The cofeeding water with methanol slows down the MTH reaction, and only methyl acetate was detected after 473 K. Notably, we observe continuously rising concentrations of methane (*m*/*z* = 16), which are not further consumed in all four cases (Figures 5A–D and S25). This is a clear indication of the failure of the traditional methane-HCHO mechanism to form the first C–C bond. When starting from methanol or DME, HCHO is produced with very low efficiency only through dehydrogenation. Regrettably, acquiring a definitive indication of the initial C–C bond formation in this experimental setup is not feasible.

To avoid the misinterpretation of different compounds with the same *m*/*z* signal in Figures S27–S29, FT-IR spectra were also collected to identify the compounds occurring at different temperature ranges, as shown in Figures 5E and S30–S32. The MCR-ALS of the extracted representative spectrum profiles and their time-dependent concentration profiles shown in Figure S33 facilitate the temporal progression of intermediates with similar functional groups, like HCHO, glycolaldehyde, methyl formate, and DME. HCHO (1745, 1700 cm^–1^) and DME (2816, 1455 cm^–1^) assigned by the independent IR spectrum (Figure S30) are formed by the decomposition of DMM in the temperature range of 298–373 K. The bands at 1754 and 2876 cm^–1^, clearly detectable in two of the lowest temperature ranges, are assigned to glycolaldehyde. Finally, the 1655 cm^–1^ band is assigned to methyl formate. CO (2200–2100 cm^–1^) is generated by the dehydrogenation of HCHO in the temperature range of 373–473 K. At a higher temperature range, that is, 473–573 K, both ethylene (1608 and 2988 cm^–1^) and the cyclopentenyl cation (1506, 1386, 1368, and 2968 cm^–1^ based on a reported assignment^85^) start forming, accompanied by pronounced methane (3016 cm^–1^) and CO production. Olefins (*m*/*z* = 27) are transformed into more conjugated olefins or aromatics (i.e., coke precursors, 1595 cm^–1^) in the final two temperature ranges.

Unlike the abundant reaction intermediate detected at low temperatures with the use of DMM, neither HCHO nor other intermediates were detected in both MS and IR spectra when methanol or DME served as the initial feedstock (Figures S31 and S32). Dehydrogenation of methanol or DME to HCHO is energetically demanding, and a small amount of generated HCHO will rapidly contribute to the first C–C bond formation, and as such, HCHO itself is not detected.

Furthermore, in situ time-resolved FT-IR and mass spectrometry tandem experiments of cofeeding methanol and DMM reactants with a ratio of 1:1 were carried out to real-time trace the species evolution in H-SSZ-13 at 493 K (Figure 6A). DMM was decomposed into HCHO and DME during the first minute, and HCHO reached the highest amount at the same time and then persisted until 10–15 min. Again, glycolaldehyde (1754 cm^–1^, 1675 cm^–1^, 2876 cm^–1^) was detected after the first few minutes, confirming the M3 mechanism. Herein, 1754 cm^–1^ was assigned to glycolaldehyde in the gas phase, and 1675 cm^–1^ was assigned to the adsorbed glycolaldehyde based on the MCR-ALS analysis. Additionally, methyl acetate (*m*/*z* = 43) was detected again during the first 7 min (Figure 6B) as the stable product of ketene condensation with methanol (2845 cm^–1^, *m*/*z* = 31). The formation of methyl acetate correlates the first C–C bond formation with the production of ketene. Furthermore, both crotonaldehyde and butenone as the (cyclo)­addition products of ketene and ethylene (Figures S34 and S35) were also detected by the *m*/*z* signal of 70 and the band of 1717 cm^–1^ at 673 K (Figure S36), indicating a large amount of ketene and ethylene at high temperatures. Additionally, the consumption of HCHO accompanied by the accumulation of CO (Figure 6B) may indicate that CO is not very relevant in the formation of the first C–C bond. This is consistent with the free energy barrier comparison between the M3 mechanism and the carbonylation mechanism (Koch-carbonylation) for ketene formation reported in Table S9. Accumulation of CO may result from the dehydrogenation of HCHO over some Lewis acid sites of extra-framework Al in H-SSZ-13.

**Figure 6:**
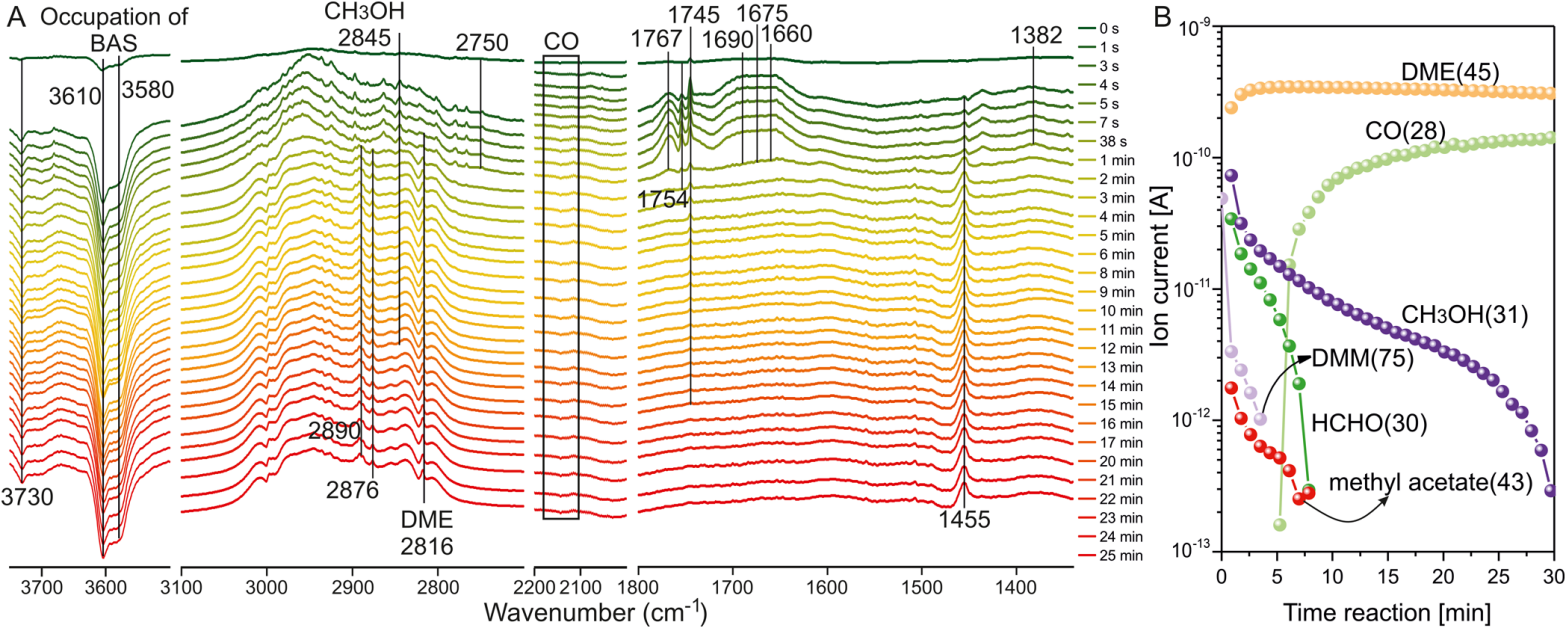
Time-resolved in situ FT-IR-MS spectra of CH_3_OH/DMM = 1 (2.666 kPa) as cofeeding reactants in H-SSZ-13 at 493 K. (A) FT-IR spectra and (B) mass spectra.

To further strengthen the plausibility of our proposed mechanisms in a more realistic MTH reaction setup, time-resolved in situ FT-IR-MS experiments were performed using methanol or DME as the reactant at 493 and 673 K (Figures S37–S40). The primary path involved the interconversion of methanol and DME at 493 K; neither HCHO nor glycolaldehyde was detected at this time. When methanol was used as the sole reactant, acetic acid, considered the most stable hydrated product of ketene (Figure S23), was produced. More importantly, crotonaldehyde was detected again by both FT-IR and MS spectra at 673 K (Figures S38 and S40), 1650 cm^–1^, and *m*/*z* = 69/70. The detection of crotonaldehyde in the experiments using DMM, DME, or CH_3_OH as the reactant implied that DMM and common MTH reactants will follow a similar reaction path to produce both ketene and ethylene with high probability (more details in Section 19 of the Supporting Information).

To confirm the validity of the proposed M3 mechanism and associated catalytic activity in other zeolites beyond H-SSZ-13, gas chromatography (GC) analysis using DMM as the reactant in H-SSZ-13, H-SAPO-34, and H-ZSM-5 was carried out from room temperature to ca. 773 K as displayed in Figure 7, along with the GC of reference gases, as shown in Figures S41 and S42. First, ethylene was detected at a lower temperature than propylene (Figures S43 and S44), indicating the priority of ethylene as the first olefin. The critical temperature for ethylene detection in three zeolites was 511 K for H-SSZ-13, 537 K for H-SAPO-34, and 538 K for H-ZSM-5, indicating the best catalytic activity of H-SSZ-13. The much higher critical temperature using methanol as the reactant in H-SSZ-13 (556 K in Figure 7A) also indicates the higher reactivity of DMM compared to methanol. This finding is consistent with the previously discussed MS and FT-IR spectra, as well as the reported temperature-programmed MS experiment in H-ZSM-5 (Si/Al = 15) by Liu et al.^42^ Furthermore, the least amount of DMM was quantitatively detected in H-SSZ-13, indicating a higher conversion of DMM than in the other two zeolites (Figure 7D–F). The better catalytic activity of H-SSZ-13 than that of both H-SAPO-34 and H-ZSM-5 is highly consistent with our calculated free energy barriers in Figure 2, 149.2 kJ/mol for H-SSZ-13, 179.0 kJ/mol for H-SAPO-34, and 173.0 kJ/mol for H-ZSM-5.

**Figure 7:**
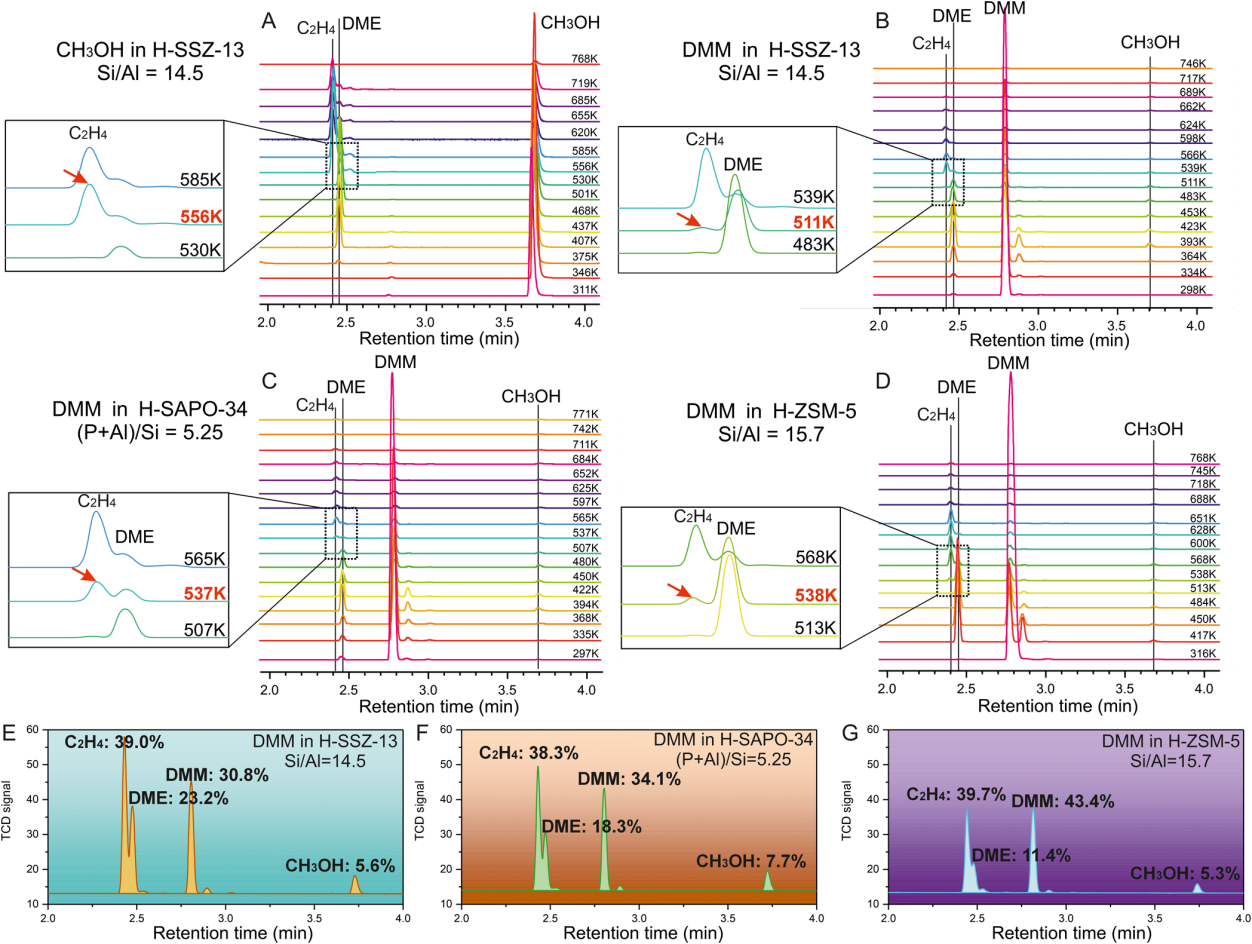
GC analysis of products using (A) CH_3_OH as the reactant in H-SSZ-13, DMM as the reactant in (B) H-SSZ-13 (Si/Al = 14.5), (C) H-SAPO-34 ((P+Al)/Si = 5.25), and (D) H-ZSM-5 (Si/Al = 15.7) at temperatures from 297 to 771 K. Quantitative analysis of products using DMM as the reactant in (E) H-SSZ-13, (F) H-SAPO-34, and (G) H-ZSM-5.

Based on the experimental observations combined with the computational results, it is concluded that the proposed M3 mechanism for the first C–C bond formation has been validated by the MS detection of 1,1,2-trimethoxyethane (P6) as the first C–C bond product when DMM is used as the reactant (Figure 1). Furthermore, both MS and FT-IR analyses of glycolaldehyde (Int3) as an intermediate validated the route for formation of ketene, as shown in Figure 4. The detection of crotonaldehyde as a common compound in experiments employing DMM, methanol, or DME as a reactant strengthens the relevance of our proposed mechanism to the realistic MTH reaction. Furthermore, the M3 mechanism in different zeolites (Figure 2) for DMM conversion was confirmed by GC analysis.

## 3 Summary

In this study, a HCHO-based mechanism for the first carbon–carbon bond formation was proposed to unravel the role of HCHO in the induction period of the MTH process. In this mechanism, the experimentally detected MDO, MOM, and DMM as methylated or hydrated products of HCHO are considered as reactants to replace methane in the parent methane-HCHO mechanism. Because of their much weaker C–H bonds compared with methane, they are expected to significantly reduce the barrier of the first C–C bond formation. Theoretical calculations of the free energy barriers confirmed the feasibility of the newly proposed mechanism to form the first C–C bond with HCHO as a key intermediate. MDO, in particular, showed the highest reactivity for C–C bond formation, regardless of the zeolite topology. SSZ-13, RUB-50, and β demonstrated the highest catalytic activity in this first C–C bond formation process. It is important to note that the acidity, pore structure, and size of zeolite will significantly influence the reactivity of different M3 reactants, and the mechanisms previously reported in the literature (like Koch-carbonylation-based mechanism, direct mechanism, etc.) may coexist with our currently proposed mechanism in the different catalytic environments. GC analysis confirmed the better catalytic activity of SSZ-13 compared with SAPO-34 and ZSM-5 for the conversion of DMM to ethene. Furthermore, an energetically feasible route to convert the six C–C bond products (geminal diols, geminal diethers, and hemiketals) to ethylene was found. The complete cycle to form ethylene involves the following steps: initially, the first C–C bonds are formed with HCHO as the key intermediate; then, these products are converted to ketene via demethylation/dehydration and hydride transfer processes; and in the final step, ketene produces olefins via the methylation-decarbonylation route. The MS detection of 1,1,2-trimethoxyethane and both MS and FT-IR detections of glycolaldehyde as the critical intermediate of this route convincingly prove our hypothesis.

Based on the free energy calculations of the three HCHO-based mechanisms, the route using DMM (easy interconversion with MDO and MOM) as a reactant to form ethylene in H-SSZ-13, proposed as a new mechanism in this work, is much preferred over the traditional methane-HCHO and methanol/DME-HCHO mechanisms. This new mechanism can be regarded as a self-reaction of HCHO, which allows us to explain the generation and consumption of HCHO during the initial MTH process. Thus, our work shows that HCHO can not only further dehydrogenate to CO but also is itself directly involved in the first C–C bond formation via the new proposed mechanism. In accordance with the experimental findings, we firmly established a reaction network among HCHO, glycolaldehyde, and ketene as the experimentally detected species through the proposed mechanism. Furthermore, the shorter induction period achieved through cofeeding HCHO in the MTH process can be explained by the current mechanisms being implemented. It is worth noticing that the proposed HCHO reaction network within zeolites can also be involved in the conversion of other C1 molecules in zeolitic systems or zeolite-based catalysts, like the CO_*x*_-to-hydrocarbons (*x* = 1 or 2) process.

## Supplementary Material



## Data Availability

Original FT-IR and MS data generated in this study have been deposited in the RODBUK repository at 10.57903/UJ/ISQT9Z, and all computational data have been deposited in the Zenodo repository at 10.5281/zenodo.15437171.

## Notes

Additional computational and experimental details, including DFT and AIMD methods, spectroscopic analyses (FT-IR, MS, MCR-ALS), bond dissociation energies, reaction pathways (M1–M3), thermodynamic parameters, and benchmarking data (PDF)
