# Supplementary material for: Formaldehyde-Mediated Initial Carbon–Carbon Bond Formation in Zeolite-Catalyzed Methanol-to-Hydrocarbon Conversion
Source: J Am Chem Soc. 2025 Jul 1;147(28):24719–33. doi: 10.1021/jacs.5c06141 (PMC12272692; doi:10.1021/jacs.5c06141)
Supplement: Supplementary file 1 [file ja5c06141_si_001.pdf]

Supporting Information for

Formaldehyde-Mediated Initial Carbon–Carbon Bond Formation in Zeolite-Catalyzed Methanol-to-Hydrocarbons

Wei Chen,[1] Julia Sobalska,[2,4] Wenqian Fu,[3] Karolina A. Tarach,[2] Massimo Bocus,[1] Tiandi Tang,[3] Kinga Góra-Marek,[2,*] Veronique Van Speybroeck[1,*]

[1]Center for Molecular Modeling, Ghent University, Technologiepark 46, 9052 Zwijnaarde, Belgium

[2]Faculty of Chemistry, Jagiellonian University in Kraków, Gronostajowa 2, 30-387 Kraków, Poland

[3]Jiangsu Key Laboratory of Advanced Catalytic Materials and Technology, School of Petrochemical Engineering, Changzhou University, 213164, Changzhou, Jiangsu, PR China

[4]Doctoral School of Exact and Natural Sciences, Jagiellonian University in Kraków, Łojasiewicza 11, 30-348 Kraków, Poland

*E-mail: kinga.gora-marek@uj.edu.pl; veronique.vanspeybroeck@ugent.be

Table of Contents

1. Computational Methods 3

  1.1 Static DFT calculations 3

  1.2 AIMD simulations 4

2. Experimental Details 4

  2.1 Zeolite preparation 4

  2.1 Fourier transform infrared spectroscopy and mass spectrometry analysis supported by MCR-ALS 5

  2.2 Gas chromatography 6

3. BDE of C-H bond and C-C bond 7

4. Formation and Interconversion of three M3 reactants from formaldehyde 8

5. Thermodynamics analysis of the first C-C bond formation in different zeolites 12

6. Flexibility of reactants in the M3 mechanism 13

7. Geometrical structure search of products for M3 mechanism 17

8. Barrierless step of P1-P6 to Int2 and Int3 18

9. P1-P6 to ketene 19

10. Interconversion of different M3 products via dehydration & methylation 21

11. Ethylene formation from M3 mechanism 22

12. Ethylene formation from M1 mechanism 28

13. Ethylene formation from M2 mechanism 29

14. Comparison between M3 mechanism and carbonylation mechanism for ketene formation 34

15. Ketene hydrates 36

16. MS spectrum from NIST Chemistry book and additional analysis 38

17. Additional FT-IR analysis 44

18. MCR-ALS analysis 46

19. Time resolved FT-IR-MS analysis for methanol, DME, and DMM as reactants at 493 K and 673 K 47

20. GS analysis of gas compounds 53

21. Benchmark Study 56

References 59

1. Computational Methods

1.1 Static DFT calculations

All the zeolite structures with CHA, MFI, TON, AEI, LEV, RHO, and BETA topologies were adapted from the International Zeolite Association website. Except for the TON, the supercell of the other zeolites was set to 1 × 1 × 1, and the supercell of TON was set to 1 × 1 × 3. To consider the influence of different elemental compositions, both SSZ-13 (aluminosilicate) and SAPO-34 (aluminosilicophosphate) with the same CHA topology were considered, and the optimized lattice constants were a =13.924 Å, b = 13.927 Å, c = 15.073 Å, α = 89.91°, β = 90.11°, and γ = 120.06° for SSZ-13 and a = 13.704 Å, b = 13.710 Å, c = 14.710 Å, α = 90.06°, β = 89.99°, and γ = 120.06° for SAPO-34. To introduce Brønsted acidic sites, one silicon atom at the T1 site in SSZ-13 was replaced with an aluminum atom with Si/Al = 35, and one phosphorus atom at the T1 site in SAPO-34 was replaced with a silicon atom (Al+P)/Si = 35. For the other topologies, Al substitution at the T12 site of MFI gives ZSM-5 with Si/Al = 95, on the T3 site of TON gives ZSM-22 with Si/Al = 71, on the T3 site of AEI gives SSZ-39 with Si/Al = 47, on the T2 site of LEV gives RUB-50 with Si/Al = 53, on the T1 site of RHO gives ECR-10 with Si/Al = 47, and on the T4 site of BEA gives β with Si/Al = 63.

All DFT and AIMD simulations were performed using CP2K software version 2022.1,[1], and the revised Perdew–Burke–Ernzerhof (revPBE) functional[2-3] with consideration of Grimme's D3 dispersion corrections,[4] that is, the revPBE-D3 functional, was chosen for the DFT calculations. A triplet-zeta (ζ) valence-polarized (TZVP) basis set[5], together with the Goedecker–Teter–Hutter (GTH) pseudopotential[6], was used for the system. During the self-consistent field procedure, a 360 Ry density CUTOFF criterion with the finest grid level was employed along with multi-grid number four (NGRID 4 and REL CUTOFF 70), and the SCF convergence criterion was set to 10^{-7} a.u. During the geometry optimization and transition state search, the convergence criteria for the maximum geometry change (MAX_DR), maximum force component (MAX_FORCE), root mean square geometry change (RMS_DR), and root mean square force (RMS_FORCE) were 10^{-4} bohr, 10^{-5} $bohr^{-1}$ au, 10^{-4} bohr, 10^{-5} $bohr^{-1}$ au, respectively. To search for the transition state, both climbing image-nudged elastic band (CI-NEB) and dimer methods were used. The transition states were verified to have only one negative frequency along the

vibrational direction to connect the reactant and product based on partial Hessian vibrational analysis (PHVA). Gibbs free energies were calculated at 493 K and 673 K from PHVA, where 493 K is the temperature that enables the observation of the initial C-C bond formation by solid-state NMR experiment in SSZ-13,[7] and 673 K is the common experimental temperature of the MTH reaction. PHVA is used for absorbed structures considering the absorbed species, the Al atom in zeolites, and the four connected O-T groups only. Frequencies lower than 12 cm^{-1} were raised to that value to prevent large entropic inaccuracies because of the harmonic-oscillator approximation.[8-9] For gaseous molecules, translational and rotational degrees of freedom were also considered. During the M1-M3 mechanisms, both BAS and SMS can directly couple with formaldehyde to form the corresponding surface species, and the two routes are considered separately using BAS and SMS as active sites. All thermodynamics were obtained using Shermo software [10] based on the frequency calculations from the CP2K software. To confirm the accuracy of the revPBE-D3/TZVP level of theory, a benchmark study using the random phase approximation (RPA) result as a reference is also carried out in **Section 21**.

1.2 AIMD simulations

To obtain the free energy surfaces of $HOCH_2OH$, $HOCH_2OCH_3$, and $CH_3OCH_2OCH_3$ isomerization in SSZ-13, ab initio molecular dynamics simulations with the acceleration of the metadynamics method were carried out at 673 K, and the linked PLUMED code of version 2.9 was used to carry out the metadynamics (MTD) methods.[11] all settings about electronic structure settings were consistent with the static DFT calculations. The temperature of the AIMD simulations was controlled by a chain of five Nosé–Hoover thermostats,[12] and the integration time step was set to 0.5 fs. In the metadynamics method, two torsion angles are defined as collective variables, similar to structural scanning in the gas phase. The Gaussian hills spawned in the CVs space every 50 fs, with an initial height of 2 kJ·mol^{-1}.

2. Experimental Details

2.1 Zeolite preparation

ZSM-5 zeolite was synthesized from an aluminosilicate gel containing the structure-directing agent tetraethyl ammonium hydroxide (TPAOH) in a 5.0 L stainless steel autoclave. Typically, 61.5 g $NaAlO_2$ was dissolved in 3775 g

deionized water, and then 52.5 g NaOH was added with stirring for 60 min. Subsequently, 125 mL of 25 wt.% TPAOH aqueous solution was added slowly drop by drop and stirring was continued for 30 min. Finally, 900 g of SiO_2 was added, and the mixture was stirred for 3 h. The obtained gel was dynamically crystallized at 170 °C for 48 h. The solid product was then filtered, dried, and calcined at 550 °C for 4 h. The H-form ZSM-5 (H-ZSM-5) zeolite was obtained using an ion-exchange method. The ZSM-5 sample was initially ion-exchanged with 1 M NH_4NO_3 aqueous solution at 80 °C for 4 h, and then the supernatant solution was filtered, washed, dried at 100 °C, and calcined at 450 °C for 4 h. The exchange process was repeated twice. SAPO-34 and SSZ-13 zeolites were synthesized using an improved method based on published works.[13-14] H-form SAPO-34 and SSZ-13 (H-SAPO-34 and H-SSZ-13) zeolites were also prepared using the ion-exchange method, and the procedure was the same as that for the H-ZSM-5 zeolite described above.

2.1 Fourier transform infrared spectroscopy and mass spectrometry analysis supported by MCR-ALS

The samples were pressed into pellets (5-10 mg·cm^{-2}) and placed in a custom-made quartz IR cell connected to a mass spectrometer. The catalysts were activated to remove adsorbed molecules at 773 K for 60 min under high vacuum (10^{-5}). After cooling the system to room temperature, the sorption of reactants (MeOH (Sigma-Aldrich, 99.9%), DMM (Sigma-Aldrich, 99%), and DME (AEROS 99.99%)) was performed until the hydroxyl groups were consumed entirely. The reaction was conducted in a closed catalytic system in the 323-773 K temperature range at a rate of 2 K·min^{-1}. Surface processes were monitored by recording the spectra every minute using a Bruker Vertex 70 spectrometer equipped with an MCT detector. The spectral resolution is 2 cm^{-1}. The gas-phase products released from the catalyst surface were recorded using a mass spectrometer (QMS 200, Pfeiffer) in the range of m/z = 2-100.

Isothermal FT-IR-MS experiments were performed at both 493 K and 673 K. An excess of MeOH (Sigma-Aldrich, 99.9%) (666.7 Pa in the gas phase) was introduced into the IR cell, and the spectra were recorded every 1 s (rapid scan mode, scanner velocity 80 kHz). The released products were monitored using mass spectrometry. The same experiments were performed for mixtures of MeOH and DMM (volume ratio 1:1) and MeOH and DME (1:1).

Multivariate curve resolution-alternating least-squares (MCR-ALS) algorithm analysis was applied to distinguish both the concentration and spectrum profiles of spectroscopically active components during the processes under isothermal conditions. MCR-ALS is a methodology for data analysis that is quite useful for time-resolved experiments, and MCR-ALS analysis was applied to X-ray absorption near edge structure (XANES) results in Cu-CHA zeolite catalyst.[15] From MCR-ALS analysis of FT IR spectroscopy, we obtained two sets of data: spectral components (IR spectra in a given range representing species) and time-dependent concentration profiles (of those species). Thus, it provides new information based on the “old” set of data that is much richer than simple traces of the respective IR bands, especially for many overlapping bands of different species. In this study, MCR-ALS analysis was applied to the spectral region of overlapping C−H stretching modes (3200 – 2600 cm^{-1}) and C=O, C-C, and C-H deformation modes (1800 – 1300 cm^{-1}). The analysis was conducted using the toolbox provided in the MATLAB graphical user interface.[16-18] The singular value decomposition (SVD) algorithm was used to estimate the appropriate number of components describing cracking or coke oxidation processes. Non-negativity, unimodality, and equality constraints were applied in the analysis. The MCR-ALS algorithm iteratively optimizes the initial estimates for each spectrum and coverage until a convergence criterion of 10^{-6} is achieved.

2.2 Gas chromatography

The temperature-programmed desorption of DMM (DMM-TPD) over the zeolite was performed using a Micromeritics AutoChem ASAP 2920 instrument. In a typical run, 200 mg of the sample was loaded into a quartz tube and pretreated under helium flow (30 cm^3 min^{-1}) at 550 °C for 2 h at a heating rate of 15 °C · min^{-1}. After cooling the sample to room temperature in a helium stream, DMM vapor was introduced into the zeolite in a N_2 stream (30 cm^3 min^{-1}) and adsorbed for 30 min. The N_2 stream was then switched to a helium stream, and the sample was subsequently heated to 500 °C at a rate of 3 °C min^{-1} in a He stream. The operational procedure for the temperature-programmed desorption of CH_3OH (CH_3OH-TPD) over the zeolite was the same as that for DMM-TPD, except that CH_3OH vapor was used instead of DMM vapor. The desorbed gas was collected at set intervals and analyzed using an Agilent 7890 B GC equipped with a hydrogen

flame ion detector (FID) and a DB-FFAP capillary column (30 m, 0.25 mm diameter, and 0.25 μm film). The temperature program of the column oven was as follows: an initial temperature of 50 °C was maintained for 5 min at 50 °C, then the oven was heated to 100 °C at a rate of 20 $°C \cdot min^{-1}$. The heater and detector temperatures were 200 and 260 °C, respectively. Due to the fast detection but the low distinguishability of DB-FFAP capillary column in distinguishing ethylene and propylene, we further used PLOT-Q capillary column to observe the order of output between ethylene and propylene at the reaction condition.

3. BDE of C-H bond and C-C bond

The BDE of C-H bond for the reactant and C-C bond for the product in M1-M3 mechanisms was calculated by PBE/def2-TZVP method, and all these calculations were carried out by Gaussian 16 program.[19]

BDE(C-H) was defined as the enthalpy change of the chemical equation at room temperature:

$CH_2(OR)_2 \rightarrow CH(OR)_2\bullet + H\bullet$

BDE(C-H)= $E(CH(OR)_2\bullet) + E(H\bullet) - E(CH_2(OR)_2)$

BDE(C-C) is defined as the enthalpy change of the chemical equation at room temperature:

$ROCH_2CH(OR)_2 \rightarrow ROCH_2\bullet + CH(OR)_2\bullet$

BDE(C-C)= $E(ROCH_2\bullet) + E(CH(OR)_2\bullet) - E(ROCH_2CH(OR)_2)$

Table S1. BDE (kcal/mol) of the C-H bond in reactants and C-C bond in products for M1-M3 mechanisms by the PBE/def2-TZVP method.

Mechanisms	Reactants	BDE(C-H)	Products	BDE(C-C)
M1	CH_4	428.9	CH_3CH_2OH	352.3
			$CH_3CH_2OCH_3$	352.7
M2	CH_3OH	381.2	$HOCH_2CH_2OH$	319.7
			$HOCH_2CH_2OCH_3$	318.8
M2	CH_3OCH_3	382.0	$HOCH_2CH_2OCH_3$	318.8
			$CH_3OCH_2CH_2OCH_3$	318.0

M3	$HOCH_2OH$	375.7	$HOCH_2CH(OH)_2$	331.0
			$CH_3OCH_2CH(OH)_2$	328.9
M3	$HOCH_2OCH_3$	367.8	$HOCH_2CH(OCH_3)(OH)$	335.6
			$CH_3OCH_2CHOCH_3OH$	318.8
M3	$CH_3OCH_2OCH_3$	361.9	$HOCH_2CH(OCH_3)_2$	316.7
			$CH_3OCH_2CH(OCH_3)_2$	315.5

4. Formation and Interconversion of three M3 reactants from formaldehyde

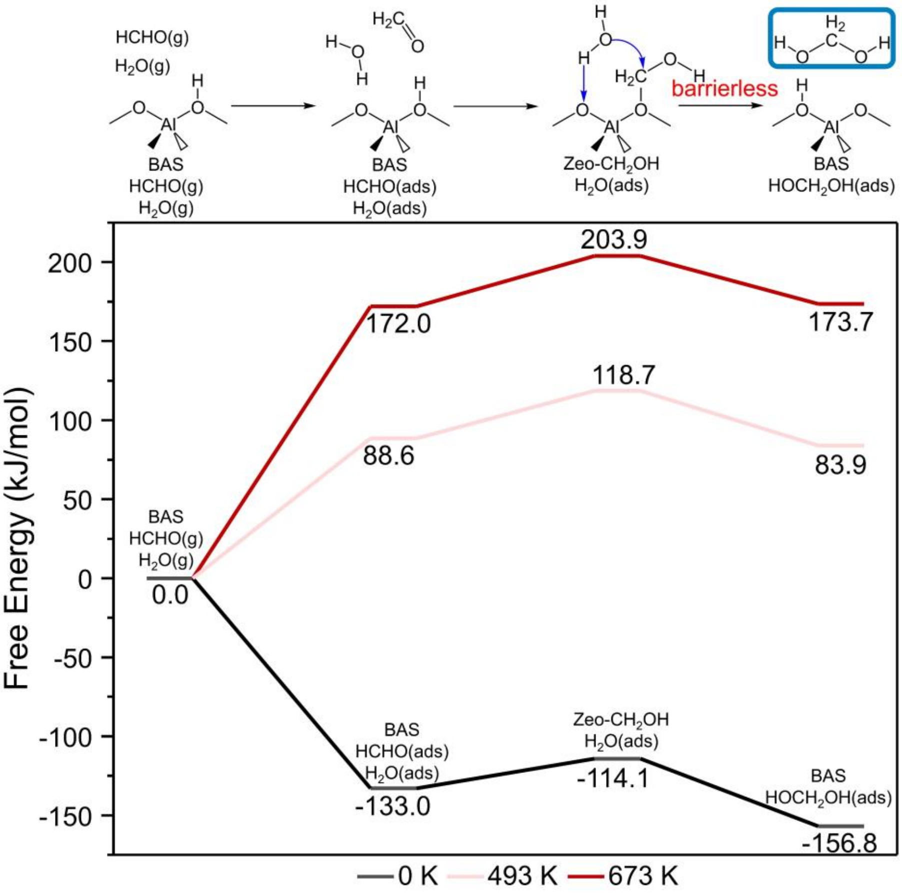

Figure S1. Free energy surface of $HOCH_2OH$ formation from the addition of formaldehyde in SSZ-13.

Methanediol ($HOCH_2OH$) was formed by adding formaldehyde to water, as shown in **Figure S1**. BAS first adsorbed water and formaldehyde, and then formaldehyde was protonated to the surface hydroxymethyl group (Zeo-CH_2OH), $HOCH_2OH$ was finally formed by the reaction between Zeo-CH_2OH and water as a barrierless step. In this pathway, adsorbed $HOCH_2OH$ is the most stable species, and the formation of $HOCH_2OH$ is thermodynamically favorable at 0 K. However, the

adsorption of formaldehyde and water on BAS at higher temperatures is difficult, especially at 673 K, leading to a significantly increased free energy for this addition reaction. Therefore, we can infer that the formation of $HOCH_2OH$ is highly sensitive to temperature, and higher concentrations of water and formaldehyde promote the formation of $HOCH_2OH$.

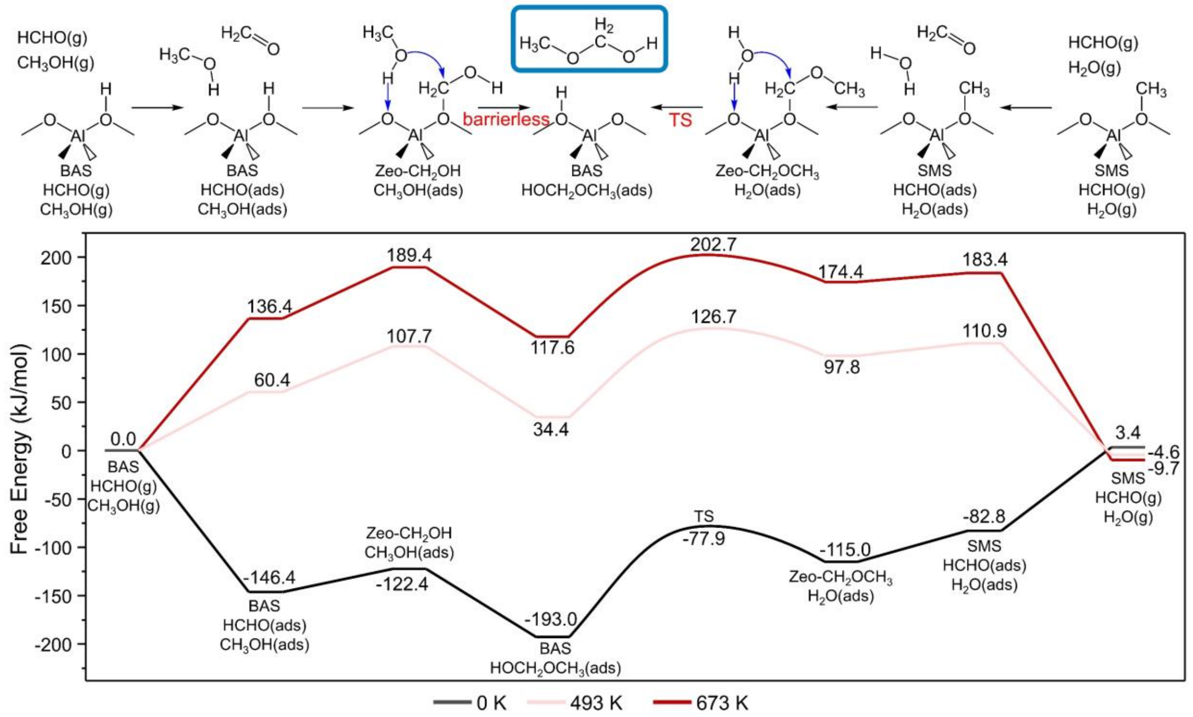

Figure S2. The free energy surface of $HOCH_2OCH_3$ was formed from the addition of formaldehyde to SSZ-13.

Methoxymethanol ($HOCH_2OCH_3$) can be formed by the addition of formaldehyde to both water on SMS and methanol on BAS, as shown in **Figure S2**. Using methanol as the addition agent, both formaldehyde and methanol were adsorbed onto the BAS, and the adsorbed formaldehyde was protonated to Zeo-CH_2OH. $HOCH_2OCH_3$ is formed by a coupling between Zeo-CH_2OH and methanol without a barrier. Using water as the addition agent, both formaldehyde and water were adsorbed onto SMS, and the adsorbed formaldehyde was protonated to the surface methoxymethyl group (Zeo-CH_2OCH_3). $HOCH_2OCH_3$ was finally formed by the reaction between Zeo-CH_2OCH_3 and water by overcoming an energy barrier of 37.1 kJ/mol. Among these two pathways, adsorbed $HOCH_2OCH_3$ is the most stable at 0 K, and the formation of $HOCH_2OCH_3$ is both thermodynamically and kinetically favorable. However, the adsorption of formaldehyde, water, and methanol at high temperatures becomes energetically unfavorable, and the preferred route of

$HOCH_2OCH_3$ requires a compensating free energy of 107.7 kJ/mol at 493 K and 189.4 kJ/mol at 673 K. Therefore, the formation of $HOCH_2OCH_3$ is also sensitive to the temperature as $HOCH_2OH$, and a lower temperature and higher reactant concentration will provide a higher possibility for the formation of $HOCH_2OCH_3$.

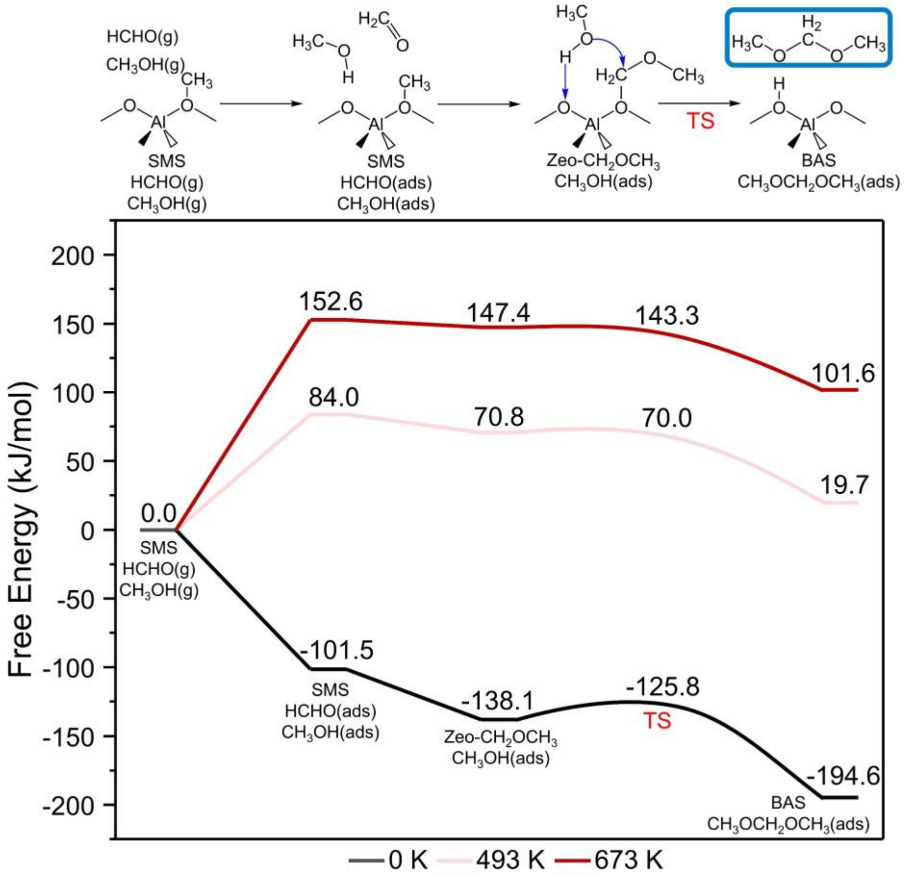

Figure S3. The free-energy surface of $CH_3OCH_2OCH_3$ was formed by the addition of formaldehyde to SSZ-13.

Dimethoxymethane ($CH_3OCH_2OCH_3$) can be formed by adding formaldehyde to methanol on SMS. Formaldehyde and methanol were first adsorbed on SMS, and then formaldehyde was protonated to Zeo-CH_2OCH_3; the reaction between Zeo-CH_2OCH_3 and methanol finally formed CH_3OCH_2OCH via a transition state. In this pathway, the adsorbed $CH_3OCH_2OCH_3$ is the most stable species and the barrier of $CH_3OCH_2OCH_3$ is only 12.3 kJ/mol at 0 K. However, the adsorption of formaldehyde and methanol on SMS at the higher temperatures will be difficult, especially at 673 K, leading to the significantly increased free energy for this addition reaction. Like $HOCH_2OCH_3$ and $HOCH_2OH$, also $CH_3OCH_2OCH_3$ is sensitive to temperature, and

higher concentrations of methanol and formaldehyde promote the formation of $CH_3OCH_2OCH_3$.

However, the formation of $CH_3OCH_2OCH_3$ is energetically more favorable than that of both $HOCH_2OCH_3$ and $HOCH_2OH$, especially at high temperatures. At 0 K, the formation of $HOCH_2OH$, $HOCH_2OCH_3$, and $CH_3OCH_2OCH_3$ is an exothermic process with the energy of -156.8, -193.0, and -194.6 kJ/mol; At 493 K, the formation of $HOCH_2OH$, $HOCH_2OCH_3$, and $CH_3OCH_2OCH_3$ is an endothermic process with the energy of 83.9, 34.4, and 19.7 kJ/mol; At 673 K, the endothermic energies are 173.7kJ/mol for $HOCH_2OH$, 117.6 kJ/mol for $HOCH_2OCH_3$, and 101.6 kJ/mol for $CH_3OCH_2OCH_3$.

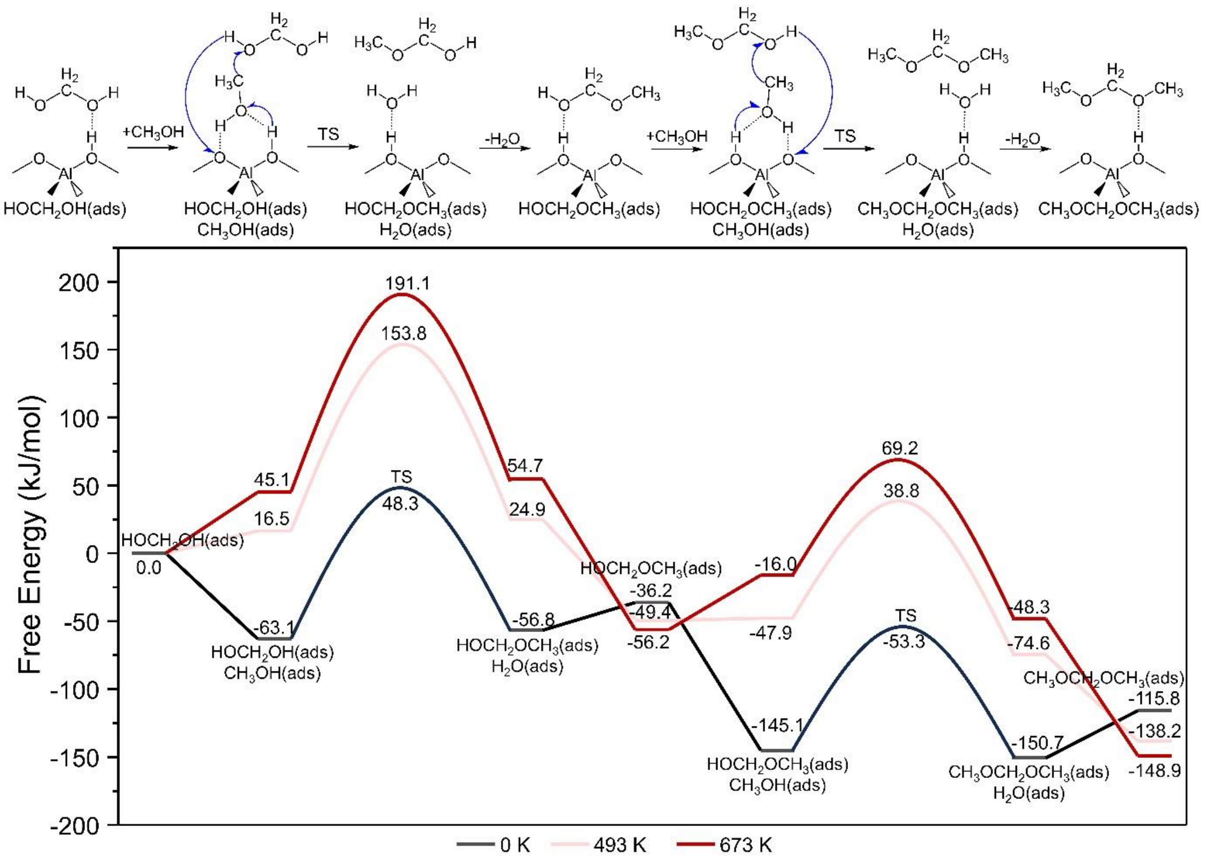

Figure S4. Free energy surface of the interconversion between $HOCH_2OH$, $HOCH_2OCH_3$, and $CH_3OCH_2OCH_3$ by the (de-)methylation process in SSZ-13 with methanol as the methylating agent.

In addition to the formation of $CH_2(OR)_2$ from the addition of formaldehyde, the three formulas of $CH_2(OR)_2$ can also be interconverted via (de-)methylation, as displayed in **Figure S4**. Their relative thermodynamic stabilities are $HOCH_2OH$ <

$HOCH_2OCH_3$ < $CH_3OCH_2OCH_3$, and the methylation from $HOCH_2OH$ to $CH_3OCH_2OCH_3$ is more favorable than the hydrolysis from $CH_3OCH_2OCH_3$ to $HOCH_2OH$, but the methylation of $HOCH_2OH$ to $HOCH_2OCH_3$ is protected by a free energy barrier of 191.1 kJ/mol at 673 K, and the further methylation of $HOCH_2OCH_3$ to fully methylated $CH_3OCH_2OCH_3$ needs to overcome a free energy barrier of 125.3 kJ/mol. These methylation processes are highly sensitive to temperature, and the low temperature of 493 K will greatly promote their interconversions, as indicated by the significantly decreased barrier of 153.8 kJ/mol and 88.3 kJ/mol. In practical experiments, the stability and concentration of $CH_2(OR)_2$ are also strongly affected by other factors such as zeolite acidity, reactant ratio, and water content. Therefore, all three $CH_2(OR)_2$ were considered possible reactants to form the first C-C bond via M3 path.

5. Thermodynamics analysis of the first C-C bond formation in different zeolites

As the most reactive reactant, the small-size $HOCH_2OH$ should only be weakly influenced by different zeolite topologies but our findings point towards a wide range of $\Delta G^{\ddagger}$ values from 108.2 kJ/mol to 152.8 kJ/mol at 673 K. To understand this unusual phenomenon, we investigated the free energy barriers at three different temperatures, as shown in **Figure S5**. Evidently, the energy barrier at 0 K varies in a smaller scope of 98.5 kJ/mol to 132.8 kJ/mol, and increasing the temperature has completely different effects on the $\Delta G^{\ddagger}$ within different zeolites. Within ECR-10, SAPO-34, ZSM-5, and RUB-50, $\Delta G^{\ddagger}$ increased significantly from 0 to 673 K (20 kJ/mol), whereas in SSZ-13, SSZ-39, and β, the increase was much smaller (less than 11.8 kJ/mol). Interestingly, $\Delta G^{\ddagger}$ in ZSM-22 decreases slightly with increasing temperature. The different trends in $\Delta G^{\ddagger}$ with temperature were interpreted as different entropic effects in the different zeolites (**Figure S6**). The entropy increase led to a slight decrease in $\Delta G^{\ddagger}$ for ZSM-22, but a substantial entropy decrease led to a significant increase in $\Delta G^{\ddagger}$.

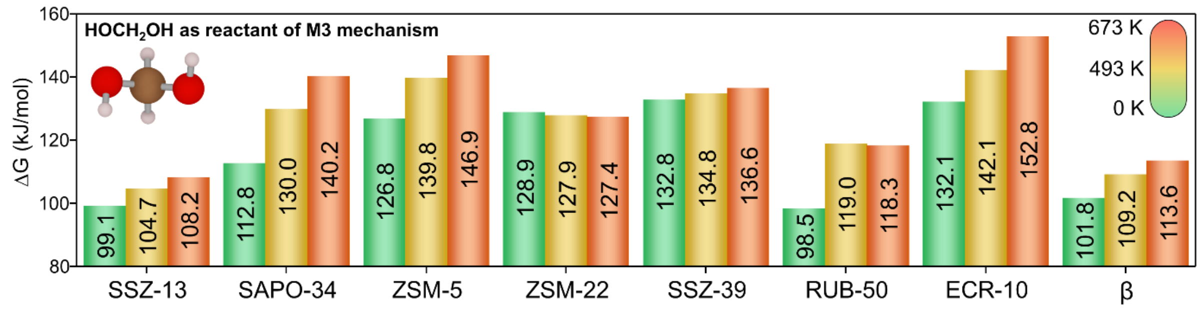

Figure S5. Free energy barriers of SMS + $HOCH_2OH$ + HCHO → $CH_3OCH_2CH(OH)_2$ in eight zeolites from 0 to 673 K.

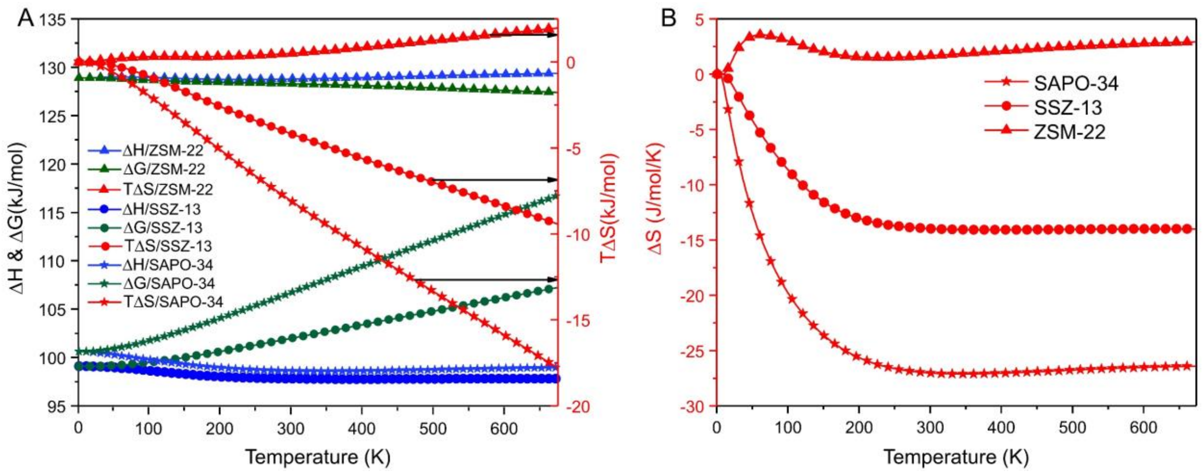

Figure S6. (A) $\Delta G^{\ddagger}$, $\Delta H^{\ddagger}$, $T\Delta S^{\ddagger}$, and (B) $\Delta S^{\ddagger}$ calculated from the difference between TS0 and Int1 for the SMS + $HOCH_2OH$ + HCHO → $CH_3OCH_2CH(OH)_2$ reaction in SSZ-13, SAPO-34, and ZSM-22 zeolites from 0 K to 673 K.

6. Flexibility of reactants in the M3 mechanism

The reactants of the M3 mechanism ($HOCH_2OH$, $HOCH_2OCH_3$, and $CH_3OCH_2OCH_3$) may exist in different isomers by rotating along two C-O bonds close to the central C atom, as illustrated in **Figure S7**. To obtain the potential energy profiles of $HOCH_2OH$, $HOCH_2OCH_3$, and $CH_3OCH_2OCH_3$ in the gas phase, relaxed structural scanning of these reactants was carried out using two torsion angles (∠O1-C1-O2-C2 and ∠O2-C1-O1-C3 in DMM) as variables, and the search range of the angle was -180° to 180°, with a scanning step size of 5°. The 2D potential energy surfaces of $HOCH_2OH$, $HOCH_2OCH_3$, and $CH_3OCH_2OCH_3$ obtained in the gas phase are displayed in **Figures S8A-S8C**, and the minimum potential energy paths are shown in **Figure S8D**.

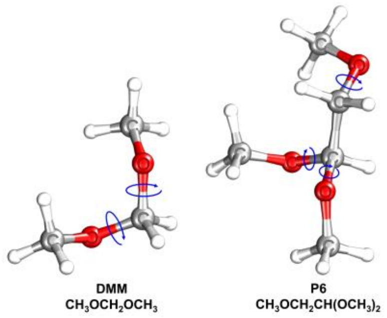

Figure S7. C–O bond rotations in DMM and P6 for dimerization during structural scanning and searching.

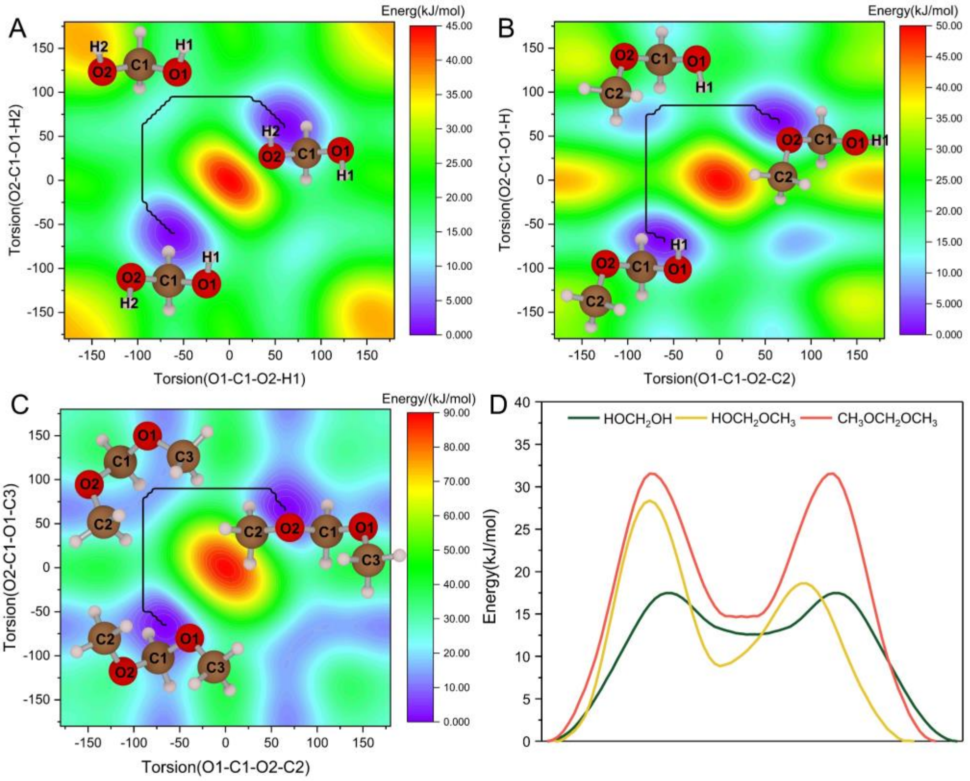

Figure S8. 2D rotation potential energy surfaces of (A) $HOCH_2OH$, (B) $HOCH_2OCH_3$, (C) $CH_3OCH_2OCH_3$, and (D) the minimum potential energy path connecting three local minima in the gas phase.

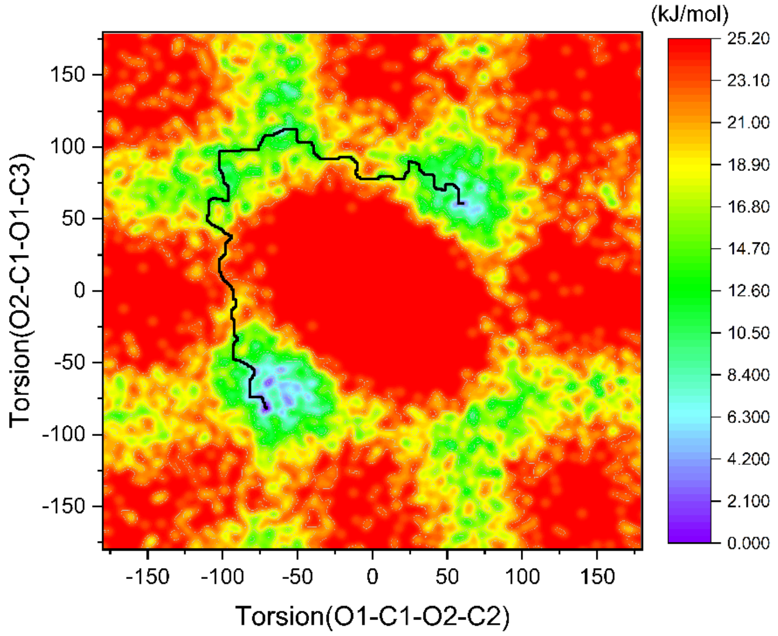

Figure S9. 2D rotation potential energy surfaces of $CH_3OCH_2OCH_3$ in SSZ-13 at 673 K.

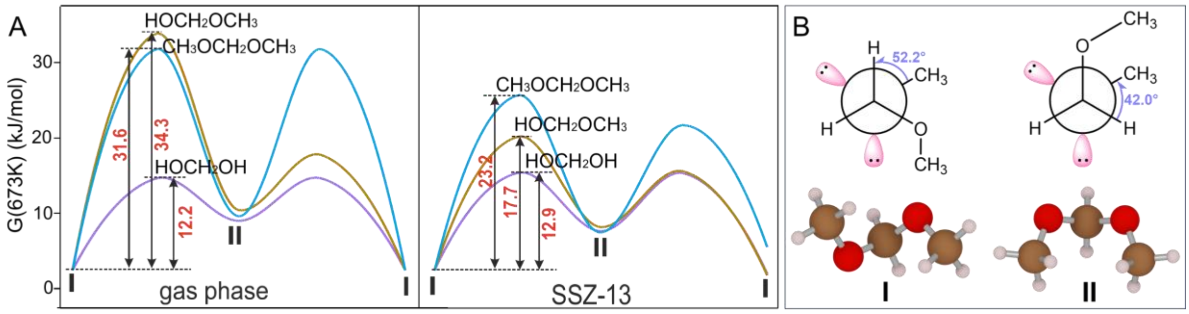

Figure S10. (A) Free energy surfaces of $HOCH_2OH$, CH_3OCH_2OH, and $CH_3OCH_2OCH_3$ rotations in the gas phase and SSZ-13 at 673 K, and (B) geometrical structures of the two minima during the rotation of $CH_3OCH_2OCH_3$.

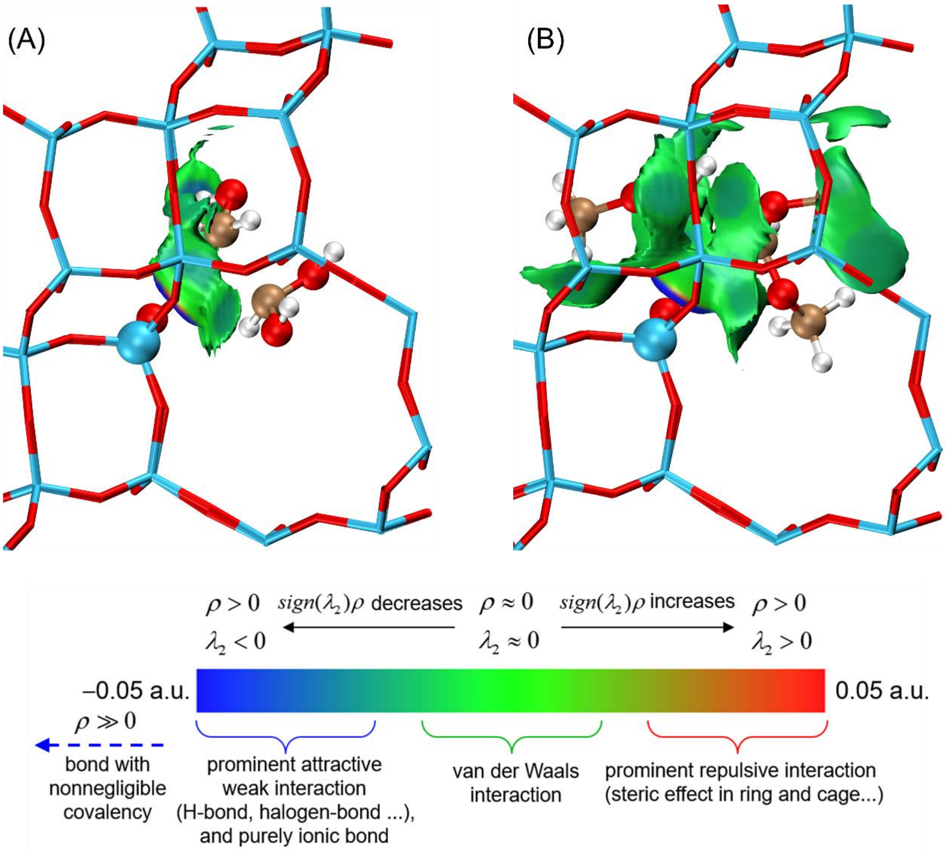

Figure S11. Weak interaction analysis of TS0 for the first C-C bond formation in the reaction of (A) HCHO + $HOCH_2OH$ → $HOCH_2CH(OH)_2$ and (B) SMS + HCHO + $CH_3OCH_2OCH_3$ → $CH_3OCH_2CH(OCH_3)_2$ + BAS.

7. Geometrical structure search of products for M3 mechanism

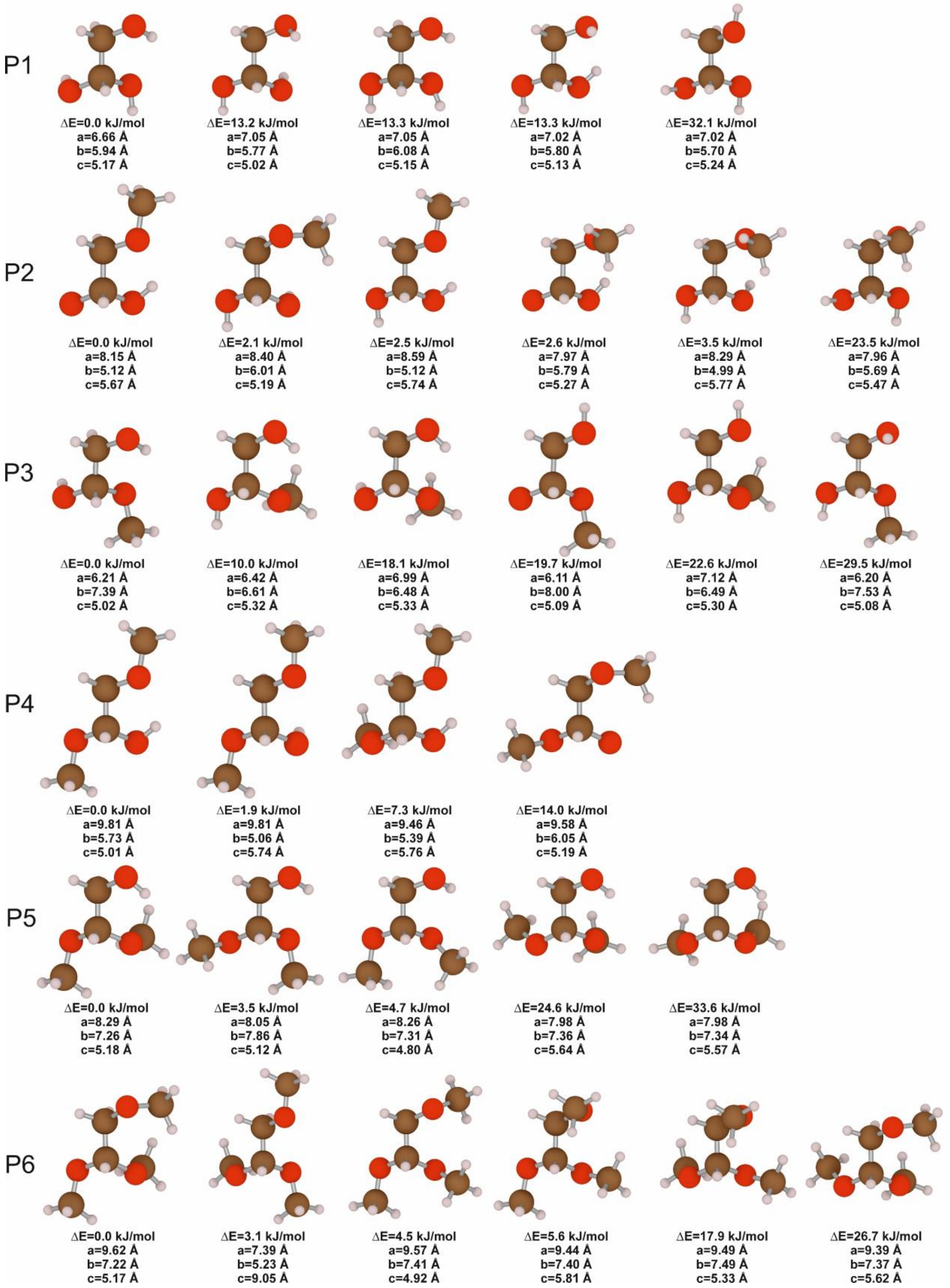

Figure S12. Geometrical structures of the isomers of the first C-C bond products (P1-P6) based on the newly proposed mechanisms in the gas phase and the energy

difference (ΔE) relative to the global minimum. a, b, and c are the side lengths of the minimum cuboids that contain each structure.

To obtain all possible isomers of P1-P6, the initial structures were generated based on the rotation of three terminal -O-R groups, with a rotation interval of 30°, and 1728 initial structures of each M3 product were finally generated. All of these structures were first optimized to the minima at the PBE-D3/TZVP level of theory, and then the reduplicative structures were removed. The most stable isomers with ΔE values < 50 kJ/mol are shown in **Figure S12**.

8. Barrierless step of P1-P6 to Int2 and Int3

CI-NEB method was employed to explore the dehydration or demethylation process of P1-P6, and 15 geometries were created by performing a linear interpolation between the initial (P1-P6) and final (Int2/Int3) states. Then, 13 geometries between the initial and final states were optimized based on the CI-NEB method, and all six optimized paths were generated, as displayed in **Figure S13**. Herein, the energy curves along with the reaction coordinate are not traditional parabolas, but fluctuating rising curves. The dehydration or demethylation of P1-P6 is an endothermic process with an energy difference smaller than 45.3 kJ/mol at 0 K, and the final state is always the maximum point in all six paths. All these fluctuations originate from multiple hydrogen bonding interactions between BAS and P1-P6 during the hydrogen transfer process. The largest fluctuation in these paths occurs in the demethylation process of P3 to Int3A, but the fluctuation in energy during this process is only 35.5 kJ/mol, as indicated in **Figure S13**. In this context, all processes of P1-P6 to Int2/Int3 can be considered barrierless.

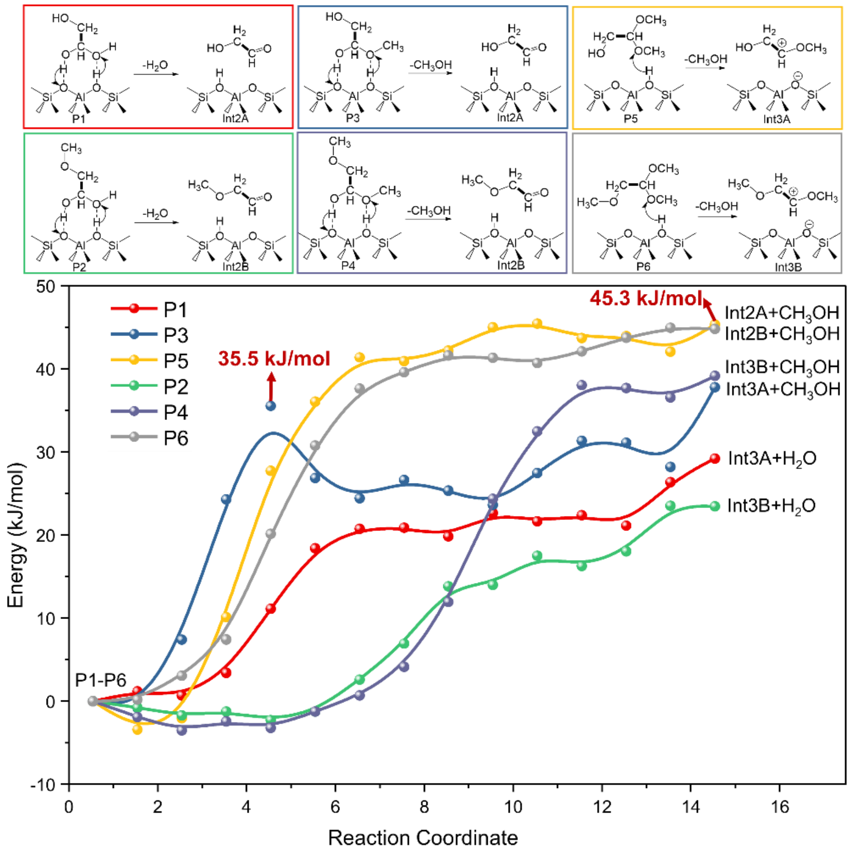

Figure S13. The converged potential energy surface of different C-C products to Int2 and Int3 obtained by the CI-NEB method.

9. P1-P6 to ketene

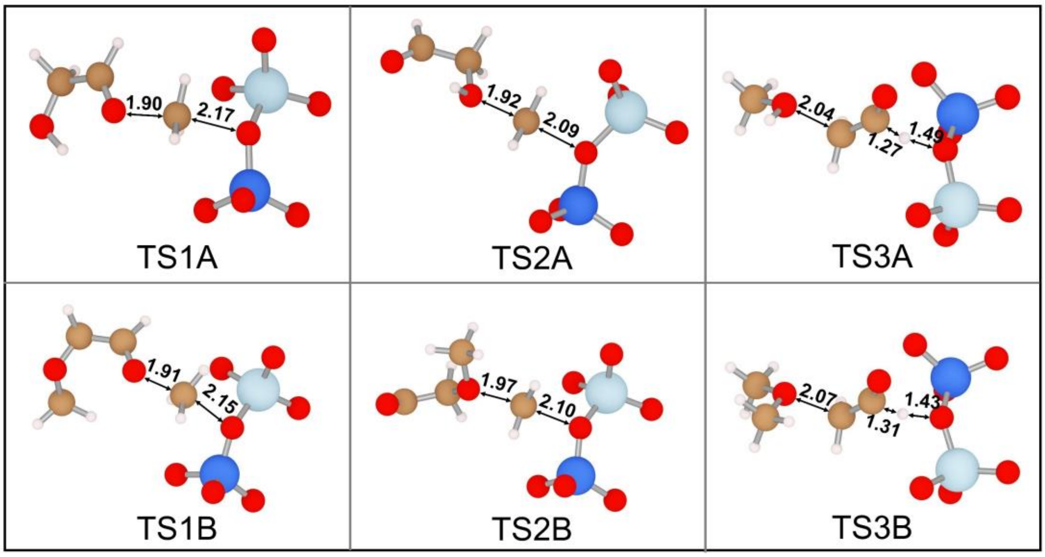

Figure S14. The related transition-state structures (TS1A, TS2A, TS3A, TS1B, TS2B, and TS3B) are shown in **Figure 3**. Unit of length: Å.

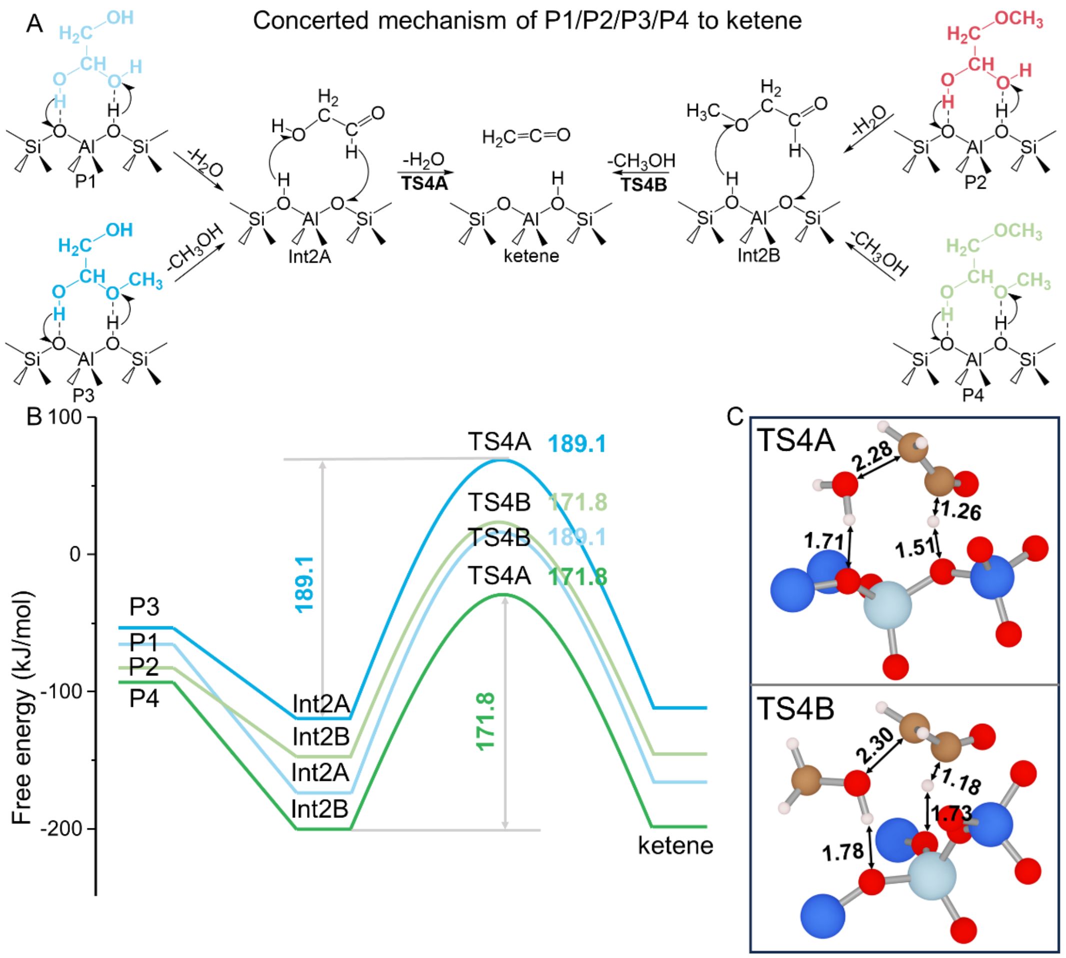

Figure S15. Concerted mechanism of P1/P2/P3/P4 to ketene. (A) Reaction pathway, (B) free-energy surface, and (C) transition-state structures (unit of length: Å) in zeolite SSZ-13.

10. Interconversion of different M3 products via dehydration & methylation

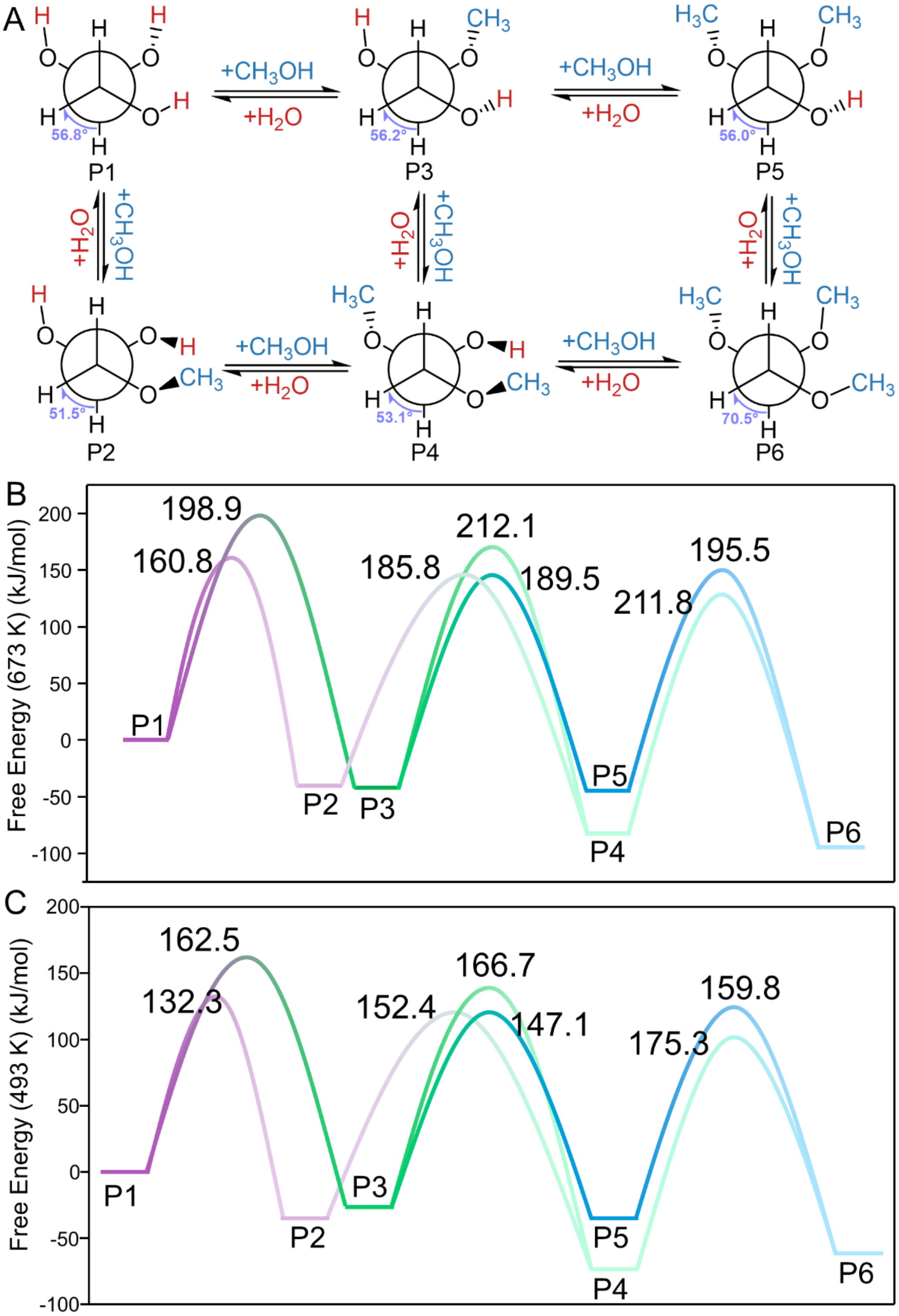

Figure S16. (A) Reaction network of the interconversion of different M3 products and the corresponding free-energy surfaces at (B) 673 K and (C) 493 K.

11. Ethylene formation from M3 mechanism

Table S2. Gibbs free energy values (kJ/mol) of conversion of $HOCH_2OH$ and HCHO to C_2H_4 in SSZ-13 at 673 K.

Species	Name	G (673K)	Species	Name	G (673K)
BAS+HCHO+$HOCH_2OH$	RA(MOD)	0.0	SMS+HCHO+$HOCH_2OH$	RB(MOD)	0.0
Zeo-CH_2OH+$HOCH_2OH$	Int1A	48.3	Zeo-CH_2OCH_3+$HOCH_2OH$	Int1B	1.0
TS0A	TS0A	154.3	TS0B	TS0B	108.2
BAS+$HOCH_2CH(OH)_2$	P1	-65.7	BAS+$CH_3OCH_2CH(OH)_2$	P4	-93.4
BAS+$HOCH_2CHO$	Int2A	-173.9	BAS+CH_3OCH_2CHO	Int2B	-200.4
SMS+$HOCH_2CHO$	Int4A	-173.9	SMS+CH_3OCH_2CHO	Int4B	-200.4
TS1A	TS2A	-98.0	TS1B	TS2B	-128.9
$CH_3OHCH_2CHO^+$	Int5A	-132.4	$(CH_3)_2OCH_2CHO^+$	Int5B	-168.4
TS2A	TS3A	-34.5	TS2B	TS3B	-104.9
Zeo-$COCH_3$+CH_3OH	Int6	-251.4	Zeo-$COCH_3$+CH_3OCH_3	Int6	-294.7
TS3A	TS4	-137.4	TS3B	TS4	-184.1
Zeo-COC_2H_5	Int7	-401.6	Zeo-COC_2H_5	Int7	-396.8
TS5	TS5	-264.0	TS5	TS5	-259.1
Zeo-C_2H_5	Int8	-463.0	Zeo-C_2H_5	Int8	-458.1
TS6	TS6	-346.0	TS6	TS6	-341.1
BAS+C_2H_4	C_2H_4	-460.6	BAS+C_2H_4	C_2H_4	-455.8

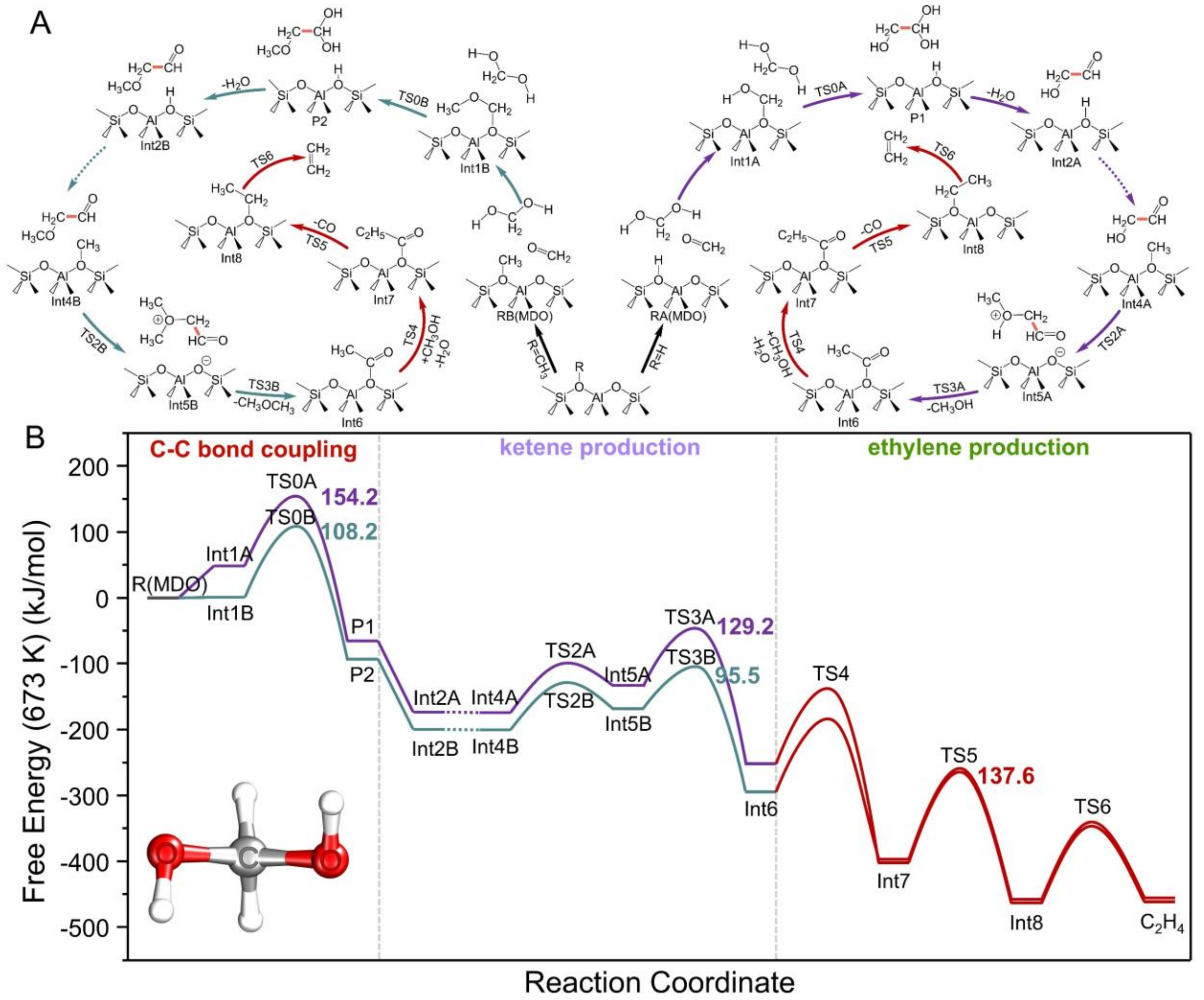

Figure S17. (A) Reaction pathways and (B) free energy surfaces of $HOCH_2OH$ to C_2H_4 via the M3 mechanism using BAS or SMS as the active sites in zeolite SSZ-13 at 673 K. Bold values in (B) are the free energy barriers determined by the maximum free energy span during C-C bond coupling, ketene production, and ethylene production.

Table S3. Gibbs free energy values (kJ/mol) of conversion of $HOCH_2OCH_3$ and HCHO to C_2H_4 in SSZ-13 at 673 K.

Species	Name	G (673K)	Species	Name	G (673K)
BAS+HCHO+$HOCH_2OCH_3$	RA(MOM)	0.0	SMS+HCHO+ $HOCH_2OCH_3$	RB(MOM)	0.0
Zeo-CH_2OH+ $HOCH_2OCH_3$	Int1A	56.3	Zeo-CH_2OCH_3+$HOCH_2OCH_3$	Int1B	5.1
TS0A	TS0A	182.6	TS0B	TS0B	117.8
BAS+$HOCH_2CH(OH)(OCH_3)$	P2	-54.0	BAS+$CH_3OCH_2CH(OH)(OCH_3)$	P5	-82.9
BAS+$HOCH_2CHO$	Int2A	-120.1	BAS+CH_3OCH_2CHO	Int2B	-147.5
SMS+$HOCH_2CHO$	Int4A	-120.1	SMS+CH_3OCH_2CHO	Int4B	-147.5
TS1A	TS2A	-44.1	TSB2	TS2B	-75.9
$CH_3OHCH_2CHO^+$	Int5A	-78.6	$(CH_3)_2OCH_2CHO^+$	Int5B	-115.4
TS3A	TS3A	19.3	TSB3	TS3B	-51.9
Zeo-$COCH_3$+CH_3OH	Int6	-197.6	Zeo-$COCH_3$+CH_3OCH_3	Int6	-241.7
TS4A	TS4	-83.6	TSB4	TS4	-131.1
Zeo-COC_2H_5	Int7	-347.8	Zeo-COC_2H_5	Int7	-343.8
TS5	TS5	-210.2	TS5	TS5	-206.2
Zeo-C_2H_5	Int8	-409.1	Zeo-C_2H_5	Int8	-405.1
TS6	TS6	-292.2	TS6	TS6	-288.2
BAS+C_2H_4	C_2H_4	-406.8	BAS+C_2H_4	C_2H_4	-402.8

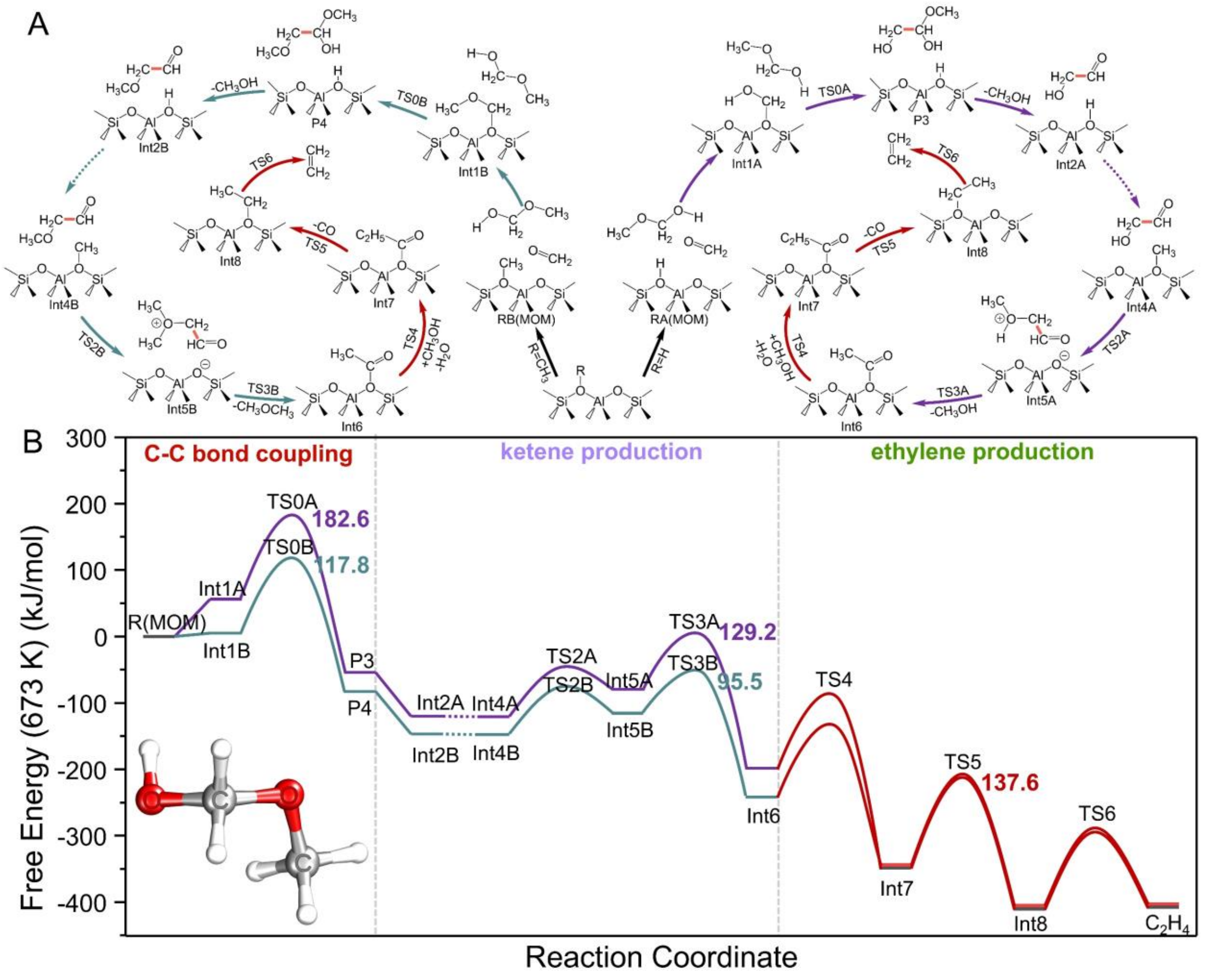

Figure S18. (A) Reaction pathways and (B) free energy surfaces of $HOCH_2OCH_3$ to C_2H_4 via M3 mechanism using BAS or SMS as active sites in zeolite SSZ-13 at 673 K. Bold values in (B) are the free energy barriers determining the maximum free energy span during the C-C bond coupling, ketene production, and ethylene production.

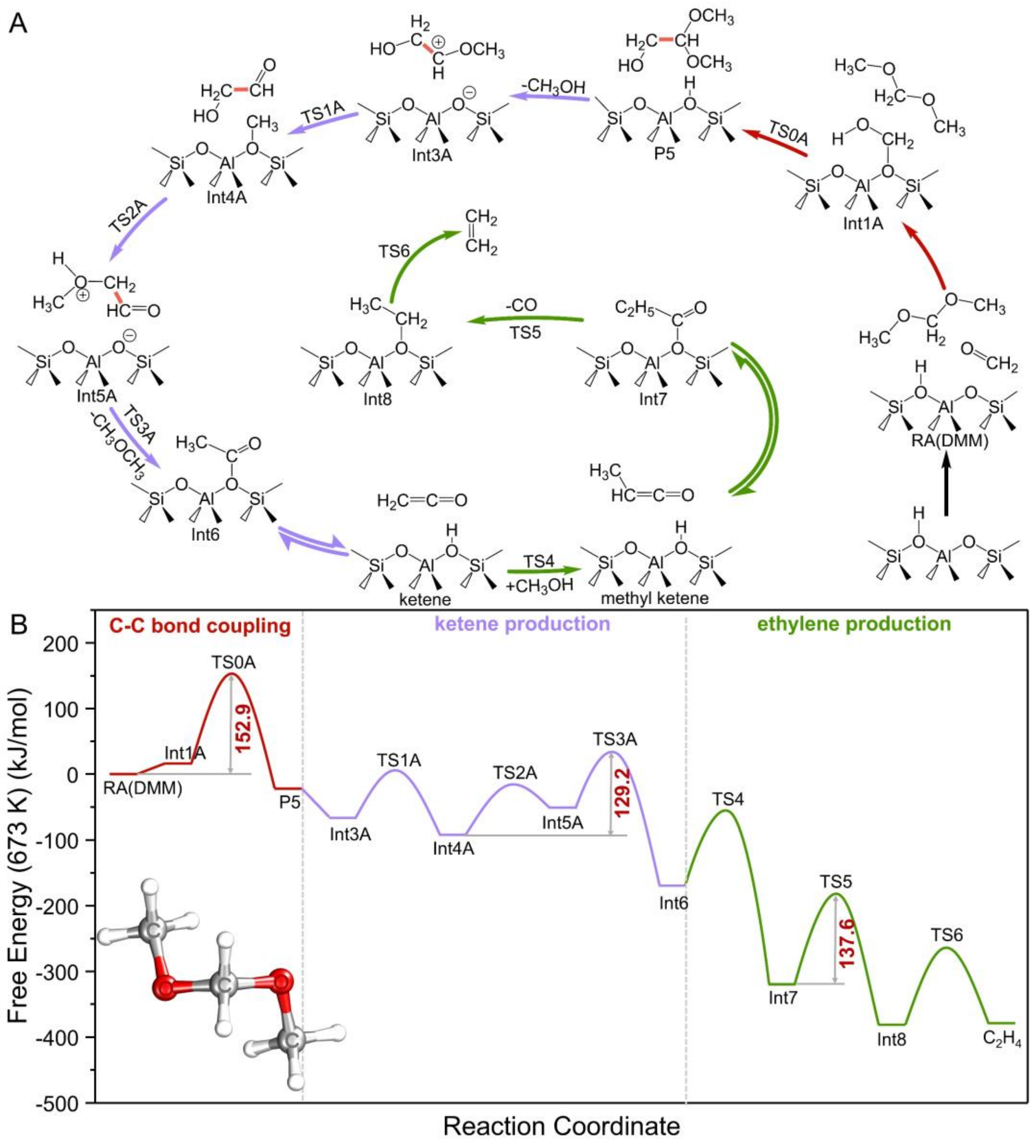

Figure S19. (A) Reaction pathways and (B) free energy surfaces of $CH_3OCH_2OCH_3$ to C_2H_4 via the M3 mechanism using BAS as the active site in SSZ-13 at 673 K. Bold values in (B) are the free energy barriers determined by the maximum free energy span during the C-C bond coupling, ketene production, and ethylene production.

Table S4. Gibbs free energies (kJ/mol) of $CH_3OCH_2OCH_3$ (DMM) and HCHO to C_2H_4 in SSZ-13.

Species	Name	G (673K)	Species	Name	G (673K)
BAS+HCHO+$CH_3OCH_2OCH_3$	RA(DMM)	0.0	SMS+HCHO+$CH_3OCH_2OCH_3$	RB(DMM)	0.0
Zeo-CH_2OH+$CH_3OCH_2OCH_3$	Int1A	16.1	Zeo-CH_2OCH_3+$CH_3OCH_2OCH_3$	Int1B	-19.1
TS0A	TS0A	152.9	TS0B	TS0B	130.1
BAS+$HOCH_2CH(OCH_3)_2$	P3	-22.1	BAS+$CH_3OCH_2CH(OCH_3)_2$	P3	-54.8
$HOCH_2CHOCH_3^+$	Int3A	-66.5	$CH_3OCH_2CHOCH_3$	Int3B	-112.1
TS1A	TS1A	5.6	TS1B	TS1B	-25.2
SMS_$HOCH_2CHO$	Int4A	-92.1	SMS_CH_3OCH_2CHO	Int4B	-106.6
TS2A	TS2A	-16.2	TS2B	TS2B	-35.1
$CH_3OHCH_2CHO^+$	Int5A	-50.6	$CH_3OCH_3CH_2CHO^+$	Int5B	-74.5
TS3A	TS3A	37.1	TS3B	TS3B	-11.0
Zeo-$COCH_3$+CH_3OH	Int6	-169.6	Zeo-$COCH_3$+CH_3OCH_3	Int6	-200.9
TS4	TS4	-55.6	TS4	TS4	-90.3
Zeo-COC_2H_5	Int7	-319.8	Zeo-COC_2H_5	Int7	-303.0
TS5	TS5	-182.2	TS5	TS5	-165.3
Zeo-C_2H_5	Int8	-381.2	Zeo-C_2H_5	Int8	-364.3
TS6	TS6	-264.2	TS6	TS6	-247.3
BAS+C_2H_4	C_2H_4	-378.8	BAS+C_2H_4	C_2H_4	-361.9

12. Ethylene formation from M1 mechanism

Methane, as a reactant for ethylene formation, goes through three transition states, and the first C-C bond via TS0 in the M1 mechanism is the rate-determining step with free energy barrier of 227.8 and 175.2 kJ/mol for BAS and SMS, respectively, as active sites in SSZ-13 at 673 K (**Figure S20** and **Table S5**). C_2H_5OH (P7A) and $C_2H_5OCH_3$ (P7B) as the C-C bond products on BAS can directly convert to surface ethyl species (SES) with the release of H_2O and CH_3OH, and SES will be deprotonated to ethene

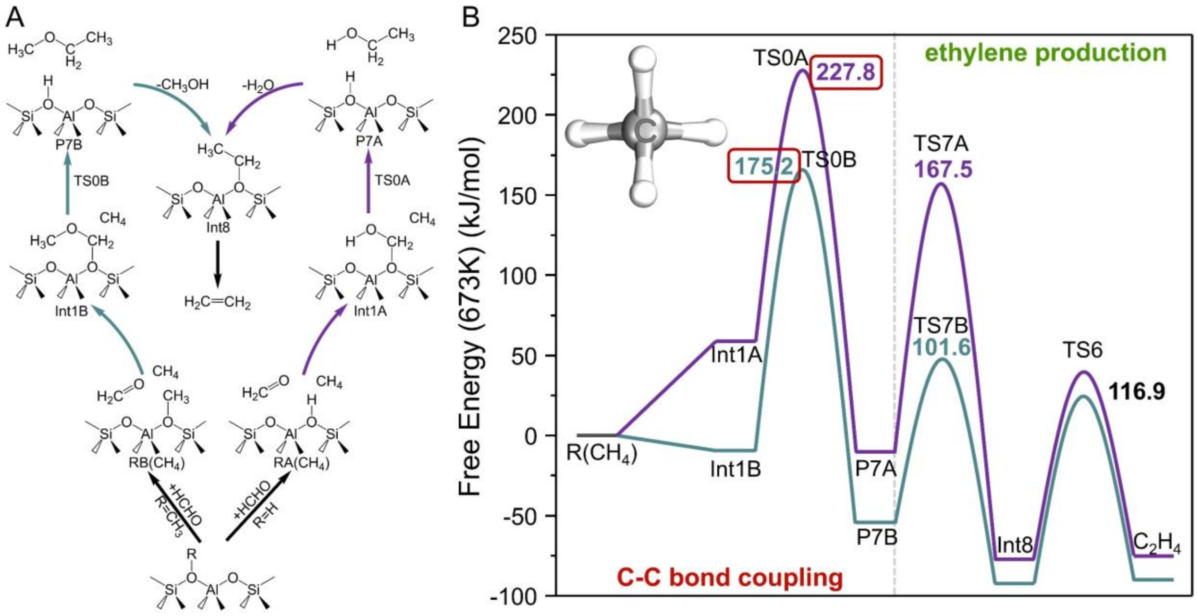

Figure S20. (A) Reaction pathways and (B) free energy surfaces of CH_4 to C_2H_4 via the M1 mechanism using BAS or SMS as the active center in SSZ-13 at 673 K. Bold values in (B) are the free energy barriers determined by the maximum free energy span during C-C bond coupling and ethylene production. The value in the red square represents the maximum barrier for this route.

Table S5. Gibbs free energy (kJ/mol) of CH_4 and HCHO conversion to C_2H_4 in SSZ-13.

Species	Name	G (673K)	Species	Name	G (673K)
BAS+HCHO+CH_4	RA(CH_4)	0.0	SMS+HCHO+CH_4	RB(CH_4)	0.0
Zeo-CH_2OH+CH_4	Int1A	58.9	Zeo-CH_2OCH_3+CH_4	Int1B	-9.4
TS0A	TS0A	227.8	TS0B	TS0B	165.8
BAS+C_2H_5OH	P7A	-10.3	BAS+$C_2H_5OCH_3$	P7B	-54.3
TS7A	TS7A	157.2	TS7B	TS7B	47.3

Zeo-C_2H_5	Int8	-77.5	Zeo-C_2H_5	Int8	-92.4
TS6	TS6	39.5	TS6	TS6	24.5
BAS+C_2H_4	C_2H_4	-75.1	BAS+C_2H_4	C_2H_4	-90.1

Table S6. Reported reaction barriers for the first C-C bond formation in the M1-M2 mechanism.

	Zeolite	Reactant	Active site	Method	Barrier (kJ/mol)	Temp (K)	Ref
M1	3T	HCHO+CH_4	BAS	BLYP/cc-pVDZ	185	0	20
M1	5T	HCHO+CH_4+H_2O	BAS	B3LYP/6-31G(d,p)	186	0	21
M1	ZSM-5	HCHO+CH_4	BAS	ωB97XD/6-	196	673	22
M1	ZSM-5	HCHO+CH_4	SMS	311+G(2df,2p)	184	673	22
M1	ZSM-5	HCHO+CH_4	BAS	ωB97XD/6-	175	573	23
M1	SSZ-13	HCHO+CH_4	BAS	31G(d,p)	190	573	23
M1	SAPO-34	HCHO+CH_4	SMS	PBE/PAW	125	0	24
M1	SSZ-13	HCHO+CH_4	BAS	revPBE-D3/TZVP	228	673	this
M1	SSZ-13	HCHO+CH_4	SMS	revPBE-D3/TZVP	175	673	work
M1	SAPO-34	HCHO+CH_4	BAS	revPBE-D3/TZVP	234	673	this
M1	SAPO-34	HCHO+CH_4	SMS	revPBE-D3/TZVP	248	673	work
M2	ZSM-5	HCHO+CH_3OH	BAS	ωB97XD/6-	181	673	22
M2	ZSM-5	HCHO+CH_3OH	SMS	311+G(2df,2p)	161	673	22
M2	ZSM-5	HCHO+DME	BAS	ωB97XD/6-	197	673	22
M2	ZSM-5	HCHO+DME	SMS	311+G(2df,2p)	179	673	22
M2	SAPO-34	HCHO+DME	SMS	PBE/PAW	95	0	24
M2	SSZ-13	HCHO+CH_3OH	BAS	revPBE-D3/TZVP	158	673	this
M2	SSZ-13	HCHO+CH_3OH	SMS	revPBE-D3/TZVP	146	673	work
M2	SAPO-34	HCHO+CH_3OH	BAS	revPBE-D3/TZVP	250	673	this
M2	SAPO-34	HCHO+CH_3OH	SMS	revPBE-D3/TZVP	249	673	work
M2	SSZ-13	HCHO+DME	BAS	revPBE-D3/TZVP	150	673	this
M2	SSZ-13	HCHO+DME	SMS	revPBE-D3/TZVP	147	673	work
M2	SAPO-34	HCHO+DME	BAS	revPBE-D3/TZVP	227	673	this
M2	SAPO-34	HCHO+DME	SMS	revPBE-D3/TZVP	193	673	work

13. Ethylene formation from M2 mechanism

The first C-C bond formation via the M2 mechanism produces three products: P8A: $HOCH_2CH_2OH$, P8B: $HOCH_2CH_2OCH_3$, and P8C: $CH_3OCH_2CH_2OCH_3$. The direct dehydration or dealcoholization of these products by BAS results in the formation of acetaldehyde; however, further conversion of acetaldehyde to ethene requires the hydrogenation of acetaldehyde to ethanol by H_2.[25] Due to the low H_2 concentration in the actual MTH process and the difficulty in dissociating the H-H bond. Fan et al[24] proposed a hydride transfer pathway for converting M2 products to C_2H_5OH and $C_2H_5OCH_3$. In this pathway, $HOCH_2CH_2OH$, $HOCH_2CH_2OCH_3$, and $CH_3OCH_2CH_2OCH_3$ will first transform two surface species (Int9A: Zeo-CH_2CH_2OH

or Int9B: Zeo-$CH_2CH_2OCH_3$) with the output of H_2O or CH_3OH by overcoming a free energy barrier of 59.7 ~ 124.4 kJ/mol and then a hydride transfer between CH_3OH (or CH_3OCH_3) and the two surface species will lead to the formation of C_2H_5OH and $C_2H_5OCH_3$ with a high barrier of 179.8~183.0 kJ/mol, as displayed in **Figures S21-S22** and listed in **Tables S6-S7**. Therefore, hydride transfer is the rate-determining step in ethylene formation via the M2 mechanism.

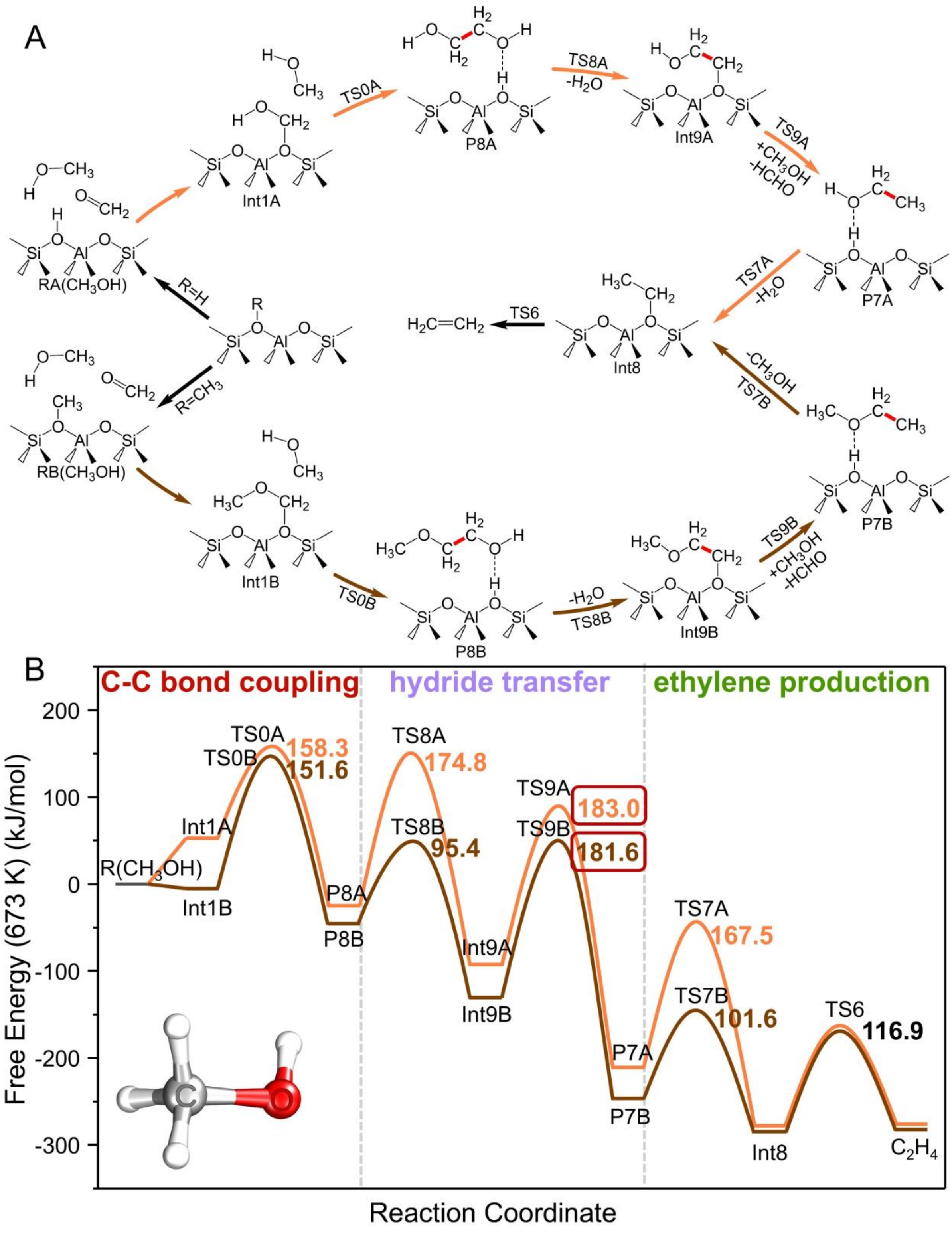

Figure S21. (A) Reaction pathways and (B) free energy surfaces of CH_3OH to C_2H_4 via the M2 mechanism using BAS or SMS as the active sites in SSZ-13 at 673 K. Bold values in hydride transfer and ethylene production of (B) are the free energy barriers determined by the difference between transition states and referred reactant states during the C-C bond coupling, and bold values in the C-C bond coupling are

the free energy barriers determined by the maximum free energy span. The value in the red square represents the highest barrier for this route. Herein, CH_3OH serves as the reactant for C-C bond formation and a hydride donor in the hydride transfer step.

Table S7. Gibbs free energy (kJ/mol) of conversion of CH_3OH to C_2H_4 in SSZ-13.

Species	Name	G (673K)	Species	Name	G (673K)
BAS+HCHO+CH_3OH	RA(CH_3OH)	0.0	SMS+HCHO+CH_3OH	RB(CH_3OH)	0.0
Zeo-CH_2OH+CH_3OH	Int1A	53.0	Zeo-CH_2OCH_3+CH_3OH	Int1B	-5.2
TS0A	TS0A	158.3	TS0B	TS0B	146.4
BAS+$HOCH_2CH_2OH$	P8A	25.2	BAS+$HOCH_2CH_2OCH_3$	P8B	-45.8
TS8A	TS8A	149.6	TS8B	TS8B	49.6
Zeo-CH_2CH_2OH	Int9A	-92.8	Zeo-$CH_2CH_2OCH_3$	Int9B	-130.6
TS9A	TS9A	90.2	TS9B	TS9B	51.0
BAS+C_2H_5OH	P7A	-211.2	BAS+$C_2H_5OCH_3$	P7B	-246.7
TS7A	TS7A	-43.7	TS7B	TS7B	-145.1
Zeo-C_2H_5	Int8	-278.4	Zeo-C_2H_5	Int8	-284.9
TS6	TS6	-161.5	TS6	TS6	-167.9
BAS+C_2H_4	C_2H_4	-276.1	C_2H_4	C_2H_4	-282.5

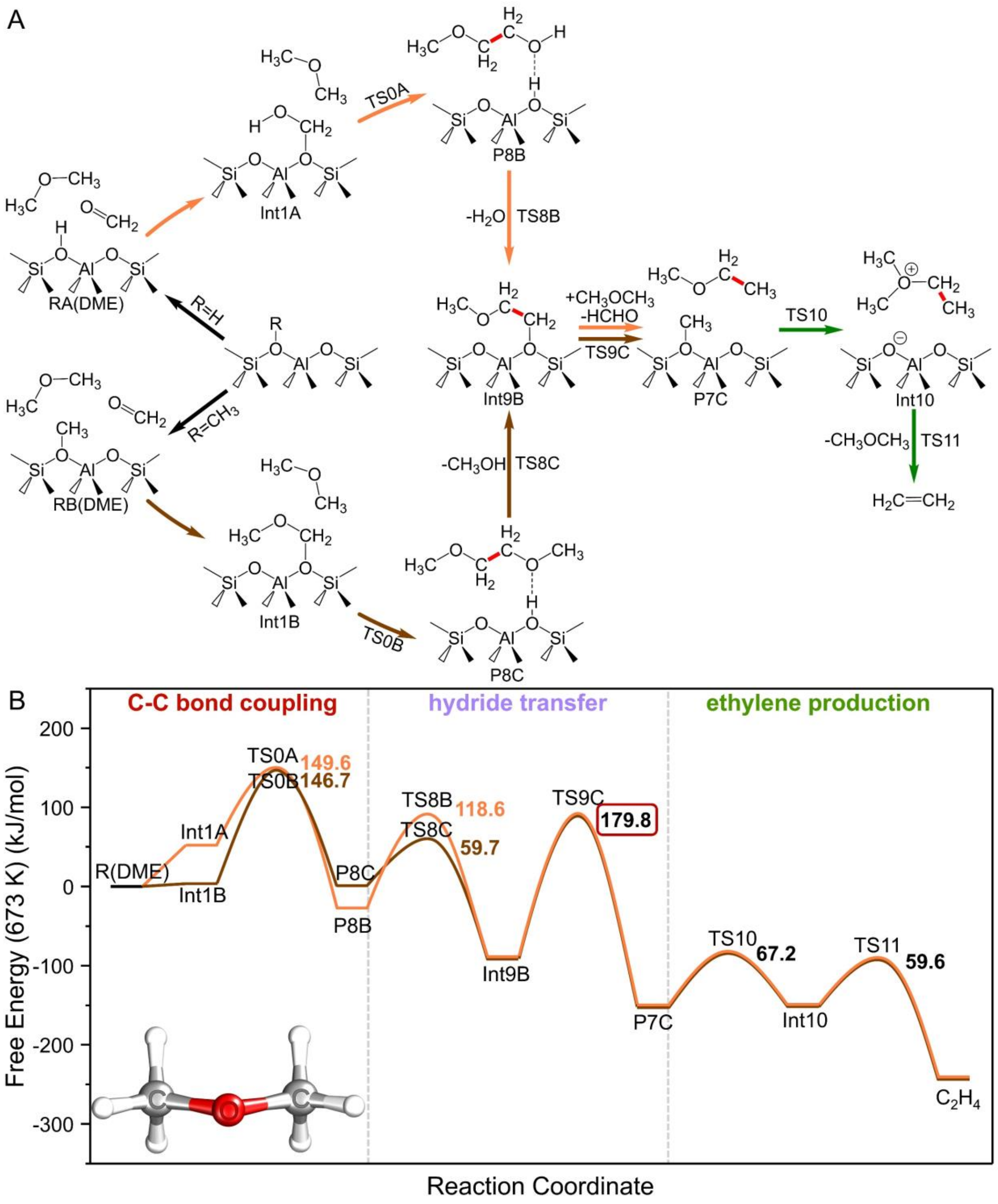

Figure S22. (A) Reaction pathways and (B) free energy surfaces CH_3OCH_3 to C_2H_4 via M2 mechanism using BAS or SMS as active sites in SSZ-13 at 673 K. Bold values in hydride transfer and ethylene production of (B) are the free energy barriers determined by the difference between transition states and referred reactant states during the C-C bond coupling, and bold values in the C-C bond coupling are the free energy barriers determined by the maximum free energy span. The value in the red square represents the highest barrier for this route. Herein, CH_3OCH_3, as the

reactant for C-C bond formation, also contributes as a hydride donor to the hydride transfer step.

Table S8. Gibbs free energy (kJ/mol) of conversion of CH_3OCH_3 to C_2H_4 in SSZ-13.

Species	Name	G (673K)	Species	Name	G (673K)
BAS+HCHO+CH_3OCH_3	RA(DME)	0.0	SMS+HCHO+CH_3OCH_3	RB(DME)	0.0
Zeo-CH_2OH+CH_3OCH_3	Int1A	52.0	Zeo-CH_2OCH_3+CH_3OCH_3	Int1B	3.6
TS0A	TS0A	149.6	TS0B	TS0B	146.7
BAS+$HOCH_2CH_2OCH_3$	P8B	-27.2	BAS+$CH_3OCH_2CH_2OCH_3$	P8C	0.8
TS8B	TS8B	91.4	TS8C	TS8C	60.5
Zeo-$CH_2CH_2OCH_3$	Int9B	-88.8	Zeo-$CH_2CH_2OCH_3$	Int9B	-91.8
TS9C	TS9C	91.0	TS9C	TS9C	88.0
SMS+$C_2H_5OCH_3$	P7C	-149.8	SMS+$C_2H_5OCH_3$	P7C	-152.8
TS10	TS10	-81.8	TS10	TS10	-84.8
$C_2H_5O(CH_3)_2^+$	Int10	-149.0	$C_2H_5O(CH_3)_2^+$	Int10	-152.0
TS11	TS11	-89.4	TS11	TS11	-92.4
BAS+C_2H_4	C_2H_4	-240.7	BAS+C_2H_4	C_2H_4	-243.7

14. Comparison between M3 mechanism and carbonylation mechanism for ketene formation

As the other mechanism for the production of ketene, Koch carbonylation mechanism for the first C-C bond formation is the C-C bond coupling between SMS and CO with the production of ketene or acetyl species. Both processes, i.e., Koch carbonylation and M3 mechanisms, produce ketene as the intermediate, which warrants the comparison of these two mechanisms. Herein, HCHO was considered as the same reactant of M3 and carbonylation mechanisms for the product of ketene in H-SSZ-13, and HCHO will go through a dehydrogenation process to produce surface formyl (Zeo-CHO) with BAS or SMS. Then Zeo-CHO will deprotonate to CO with the recovery of BAS. Finally, ketene will be formed by the C-C bond coupling between SMS and CO as described in **Figure S23A**. Our calculations indicate that the initial dehydrogenation step of HCHO is the rate-determining step, exhibiting a high barrier of 197.9 kJ/mol when a Brønsted acid site (BAS) is the active site. However, the involvement of SMS will significantly reduce this barrier to 118.0 kJ/mol, and the first C-C bond formation with the barrier of 129.4 kJ/mol will be the rate-determined (**Figure S23B**).

As listed free energy barriers (**Table S9)**, the M3 mechanism using BAS as active site is more favorable overall than carbonylation mechanism, i.e., 152.9 ~ 182.6 kJ/mol of M3 and 197.9 kJ/mol of carbonylation. When SMS is active site in two mechanisms, M3 mechanism using MDO or MOM as reactants also has the lower free energy barrier than carbonylation mechanism, but M3 mechanism using DMM as reactant is less favorable than carbonylation, and they are highly competitive. In summary, M3 mechanism is generally more favorable than carbonylation mechanism for the production of ketene.

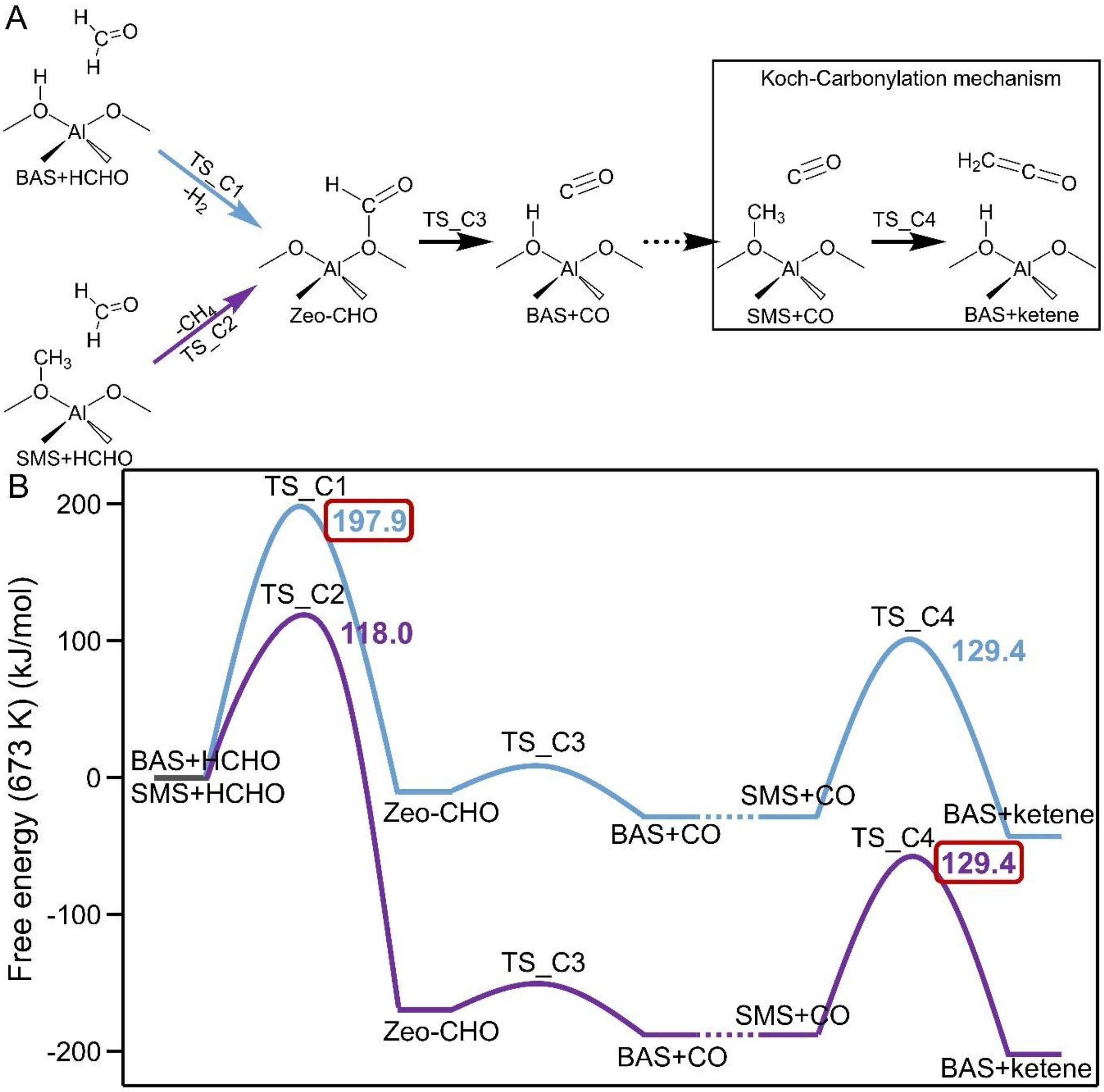

Figure S23. (A) Reaction pathways and (B) free energy surfaces HCHO to ketene via M2 mechanism using BAS or SMS as active site zeolite in SSZ-13 at 673 K.

Table S9. Free energy barrier (673 K) comparison of M3 mechanism and carbonylation mechanism for the ketene formation.

	M3 mechanism			Carbonylation
Reactants	MDO+HCHO	MOM+HCHO	DMM+HCHO	HCHO
BAS	154.2	182.6	152.9	197.9
SMS	108.2	117.8	149.2	129.4

15. Ketene hydrates

There are three hydrated products of ketene via three addition types, as displayed in **Figure S24**, and the route to acetic acid is obviously the most favorable, but the other two routes are unfavorable. However, the interconversion between acetic acid and ethene-1,1-diol is possible via hydrogen transfer, and the stable acetic acid can produce acetone by self-condensation and decarboxylation, and the formed acetone will further convert to isobutene via aldol self-condensation and cracking.[26] In this context, we can rationalize the formation of acetone via the M3 mechanism in H-SSZ-13 when methanol is the reactant (**Figure 5A**); M3 reactants will form the first C-C bond with HCHO with the production of P1-P6, and P1-P6 will transfer to glycolaldehyde with the release of water, methanol, or DME. Glycolaldehyde, as one of the hydrated products of ketene, is converted to the other ketene hydrate (acetic acid) using ketene as an intermediate, and the self-condensation and decarboxylation of the two acetic acids will produce acetone.

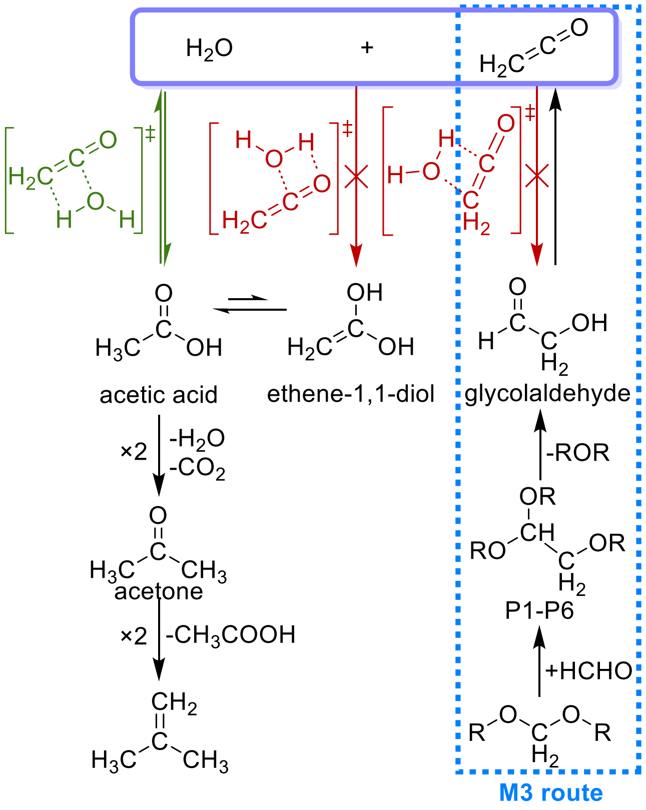

Figure S24. Three ketene hydrates and their relationships.

16. MS spectrum from NIST Chemistry book and additional analysis

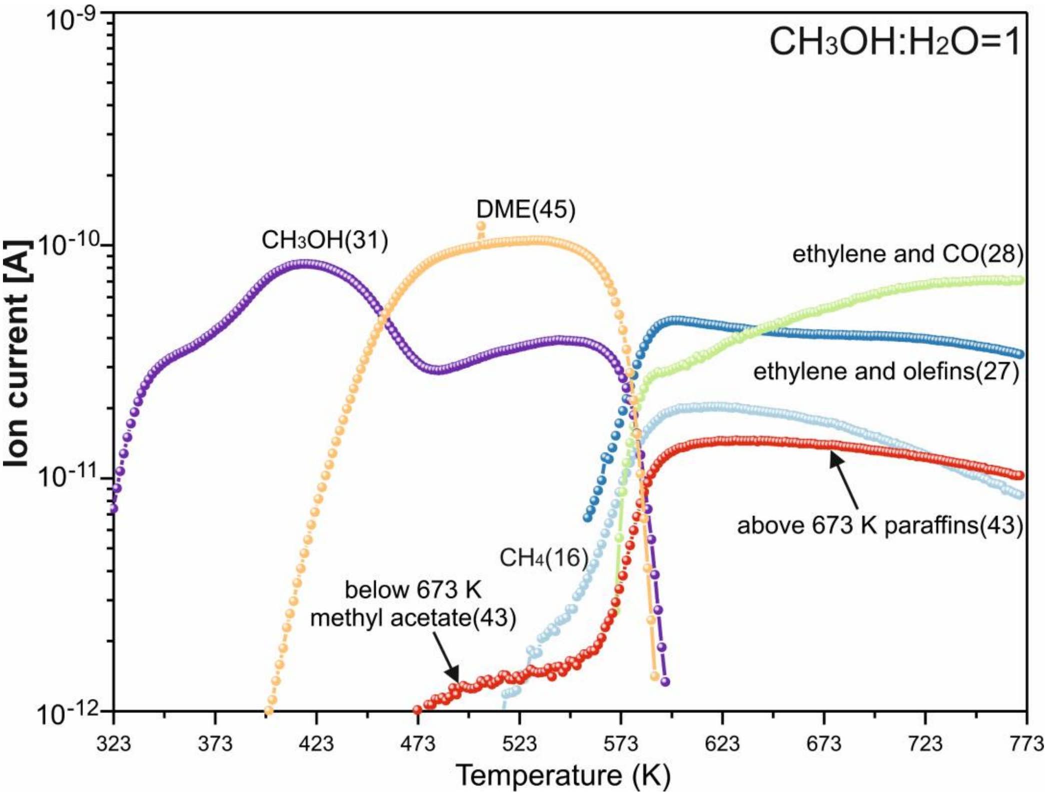

Figure S25. MS analysis of products using cofeeding $CH_3OH/H_2O=1$ as reactants in H-SSZ-13 in the temperature range 323 K-773 K.

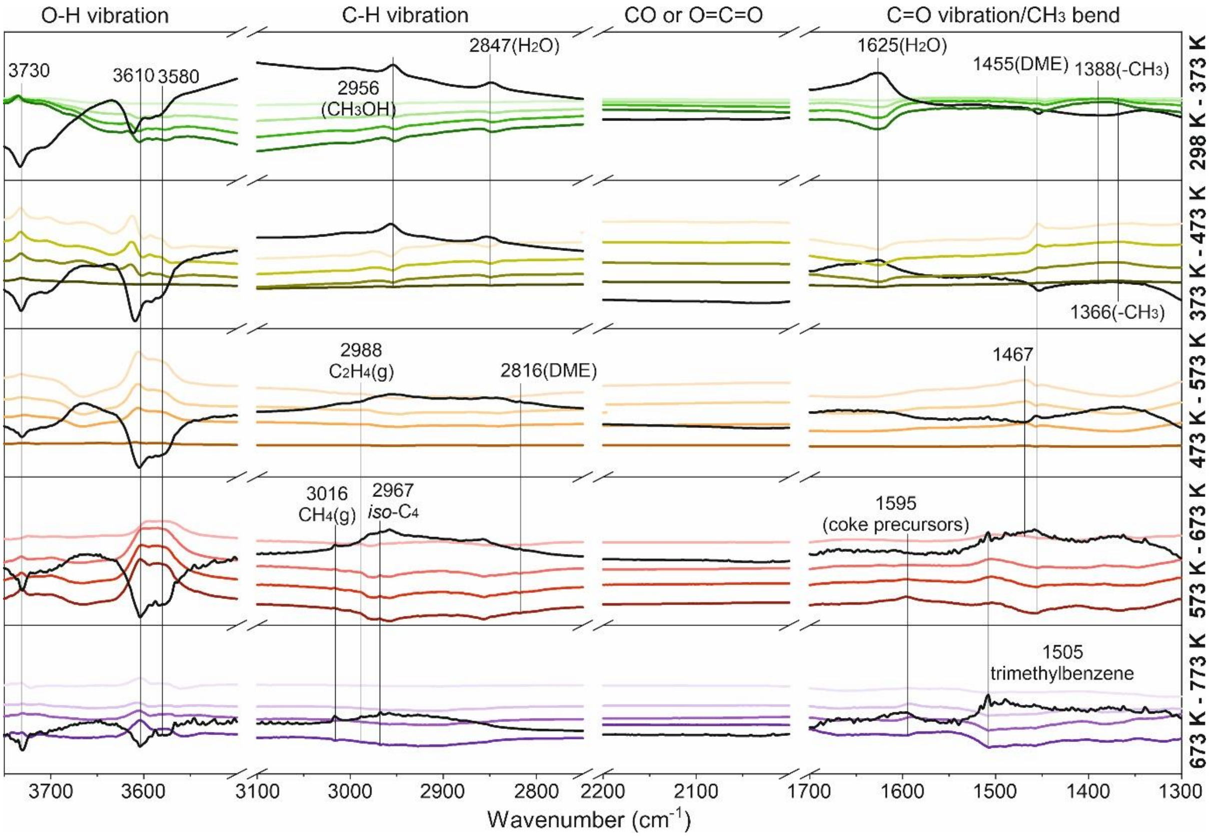

Figure S26. FT-IR spectra of $CH_3OH/H_2O=1$ as a reactant in H-SSZ-13 at temperatures of 298 K–373 K, 373 K–473 K, 473 K–573 K, 573 K–673 K, and 673 K–773 K.

The FT-IR-MS experiments using cofeeding $CH_3OH/H_2O=1$ as reactants (**Figure S25-S26**) is similar as the result using CH_3OH as reactant (**Figure 5A** and **S31**). At 298 K ~ 373 K, the band of methanol (2956 cm^{-1}) was not affected by the co-adsorption water (2847 cm^{-1}), and then DME was identified by the band of 2816 cm^{-1} at 373 K ~ 473 K but less than that without the co-feeding water (**Figure S31**). Therefore, water has an inhibiting effect on the formation of DME via the competitive adsorption of BAS. When the temperature increases to 473 K ~ 573 K, ethylene (2988 cm^{-1}) were produced with the consumption of DME. At above 573 K, methane (3016 cm^{-1}) and trace CO (2200~2000 cm^{-1}) was detected, and olefins are transformed into more conjugated olefins or aromatics (as confirmed by the increase of 1595 cm^{-1}, coke precursors).

To assign the m/z signal in the MS spectrum, we used the MS spectrum from the NIST Chemistry book as a reference. In the MS spectrum of DMM, m/z=45 was the strongest signal as the characteristic peak of DMM, but no signal of DMM was

detected (Figure 5C) because it rapidly transformed to DME and HCHO. The signal at m/z=75 was the characteristic peak of trimethoxyethane (P6), but we also noted that DMM also had a second strong peak at m/z=75 (**Figure S27A**). In our MS spectrum, the signal of HCHO (m/z=30), as the decomposition product of DMM, occurs before the signal of m/z=75, which means that m/z=75 cannot be assigned to DMM. P6 has a much stronger peak at m/z=75 than the others in **Figure S27B**, and it is much more reasonable to assign m/z=75 as the first C-C bond product between HCHO and DMM, that is, P6.

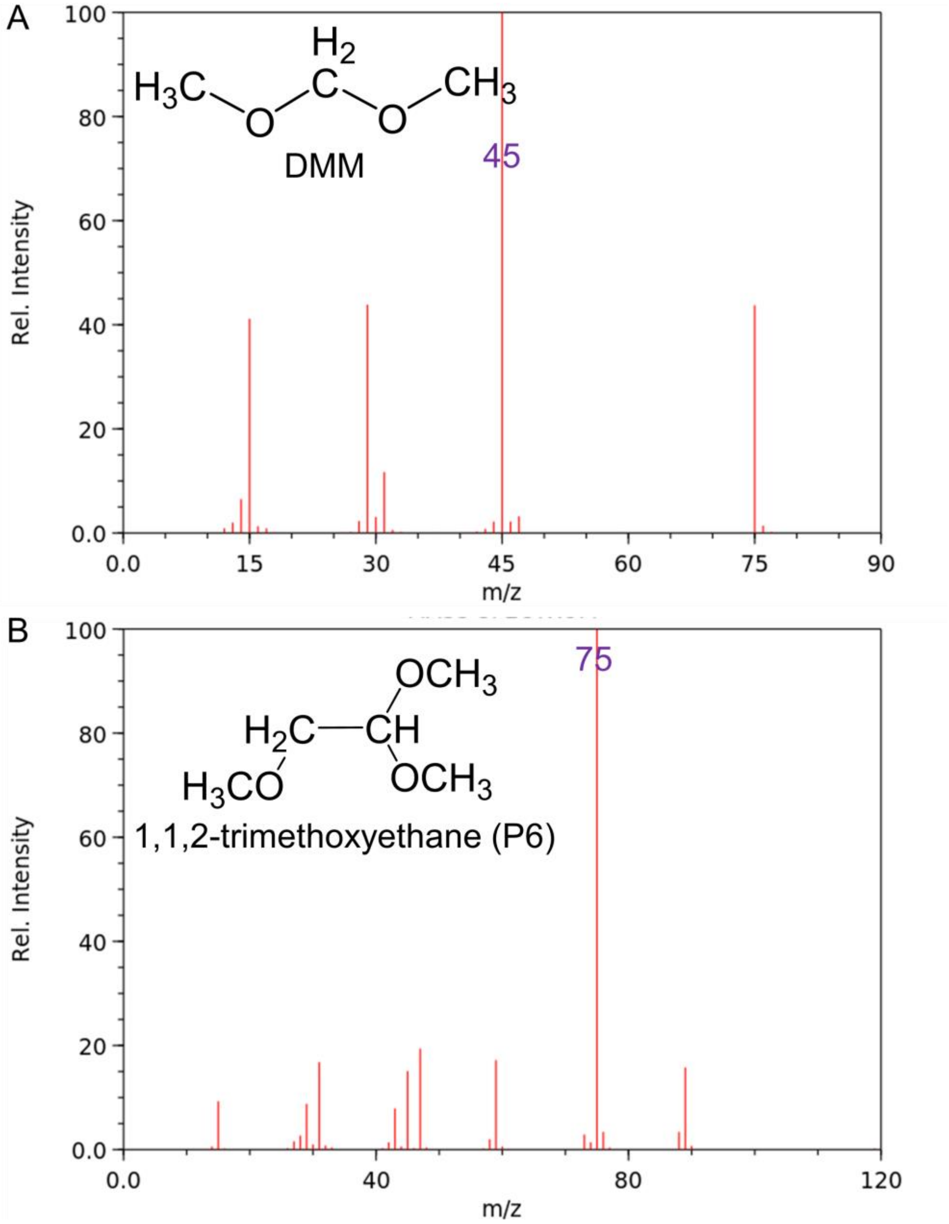

Figure S27. MS spectra of (A) DMM and (B) 1,1,2-trimethoxyethane.

Based on the referenced MS spectrum in **Figure S28**, both methyl formate and glycolaldehyde have the strongest m/z at 31, and it is difficult to distinguish where the m/z=31 comes from. However, we noted that the peaks at m/z=31 and m/z=60 were significantly increased after the consumption of P6 (m/z=75) (**Figure 5C-5D**). As the demethylation product of P6, glycolaldehyde contributed to the intensities of both m/z=31 and m/z=60. To further identify the occurrence of glycolaldehyde as an intermediate when DMM was used as a reactant, FT-IR spectroscopy was performed (**Figures 5D**, **S31-S33**).

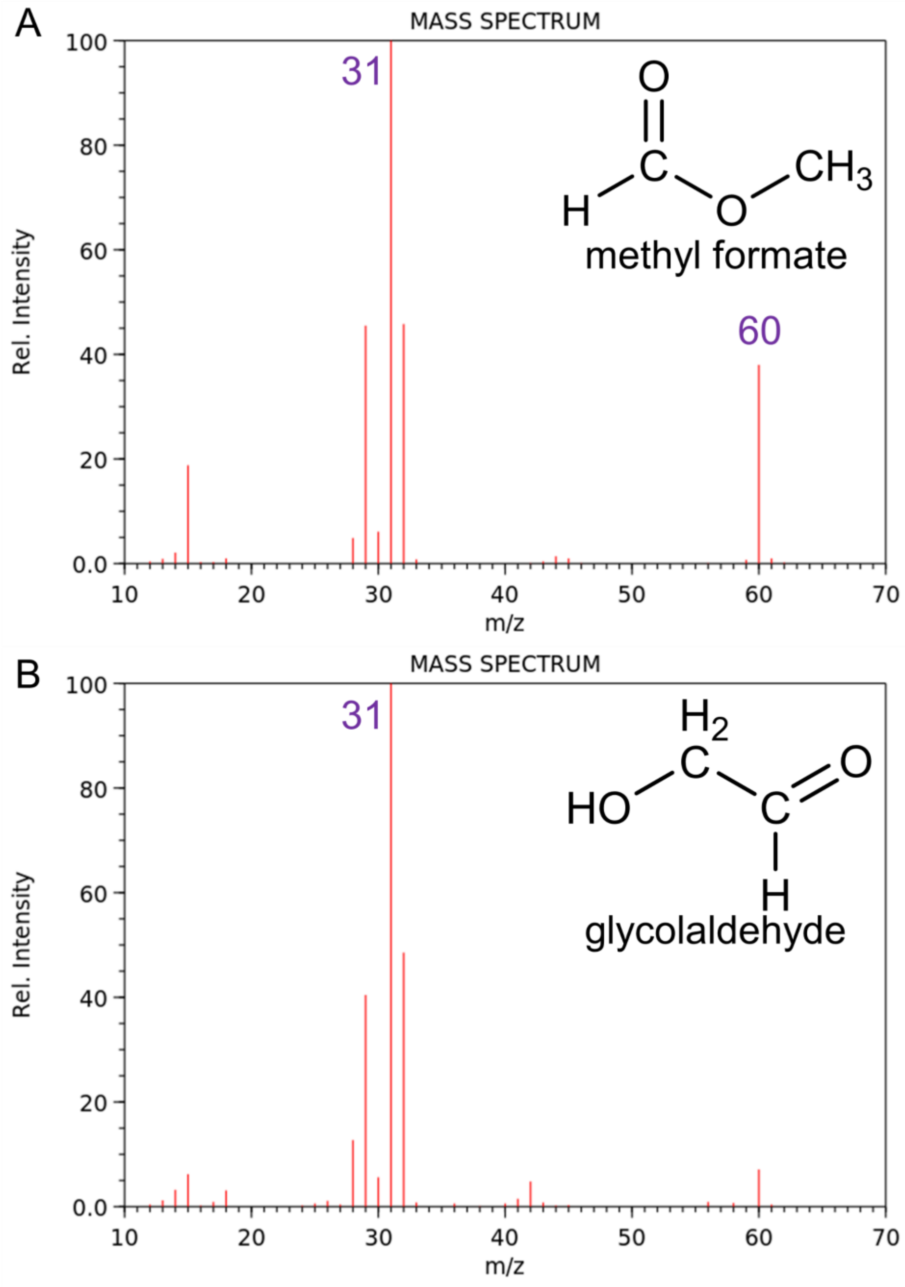

Figure S28. MS spectra of (A) methyl formate and (B) glycolaldehyde.

Both acetone and methyl acetate had the strongest signal at m/z=43, but acetone also had the secondary strongest signal at m/z=58. Therefore, the co-occurrence of m/z=43 and m/z=58 when methanol was the reactant (**Figure 5A**) was assigned to acetone. However, the sole occurrence of m/z= 43 when DMM was the reactant (**Figure 5C**) should be assigned to methyl acetate. More importantly, the signal of m/z=43 occurred and was enhanced after the consumption of P6 and glycolaldehyde (**Figures 5C-5D**), indicating a strong correlation between methyl acetate and both P6 and glycolaldehyde. The occurrence of methyl acetate can be denoted as an indicator of ketene because methyl acetate can be easily formed by the methylation of ketene with DME (m/z=45). Therefore, we can establish a reaction route for DMM to ketene based on the M3 mechanism, i.e., DMM−P6−glycolaldehyde−ketene.

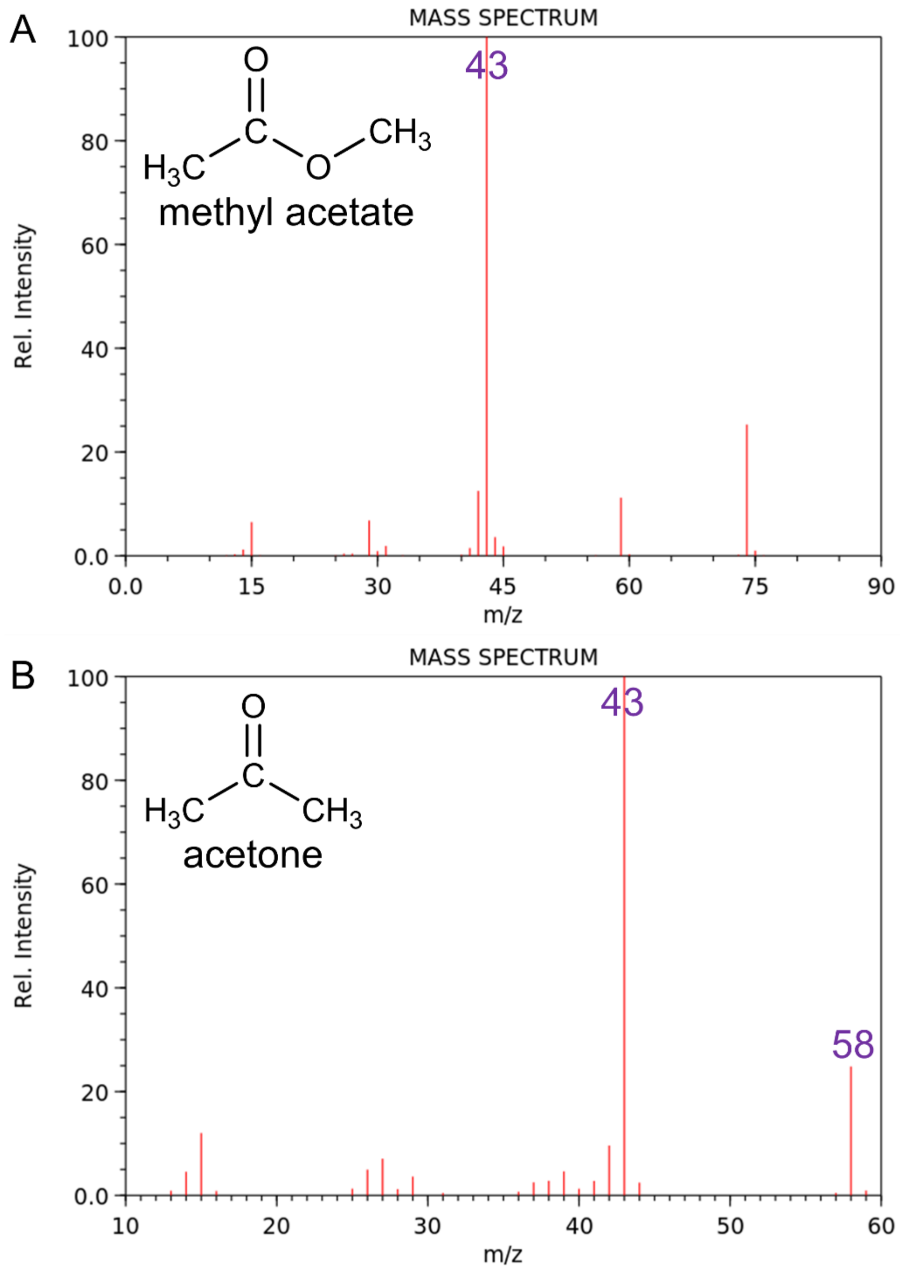

Figure S29. MS spectra of (A) acetone and (B) methyl acetate.

17. Additional FT-IR analysis

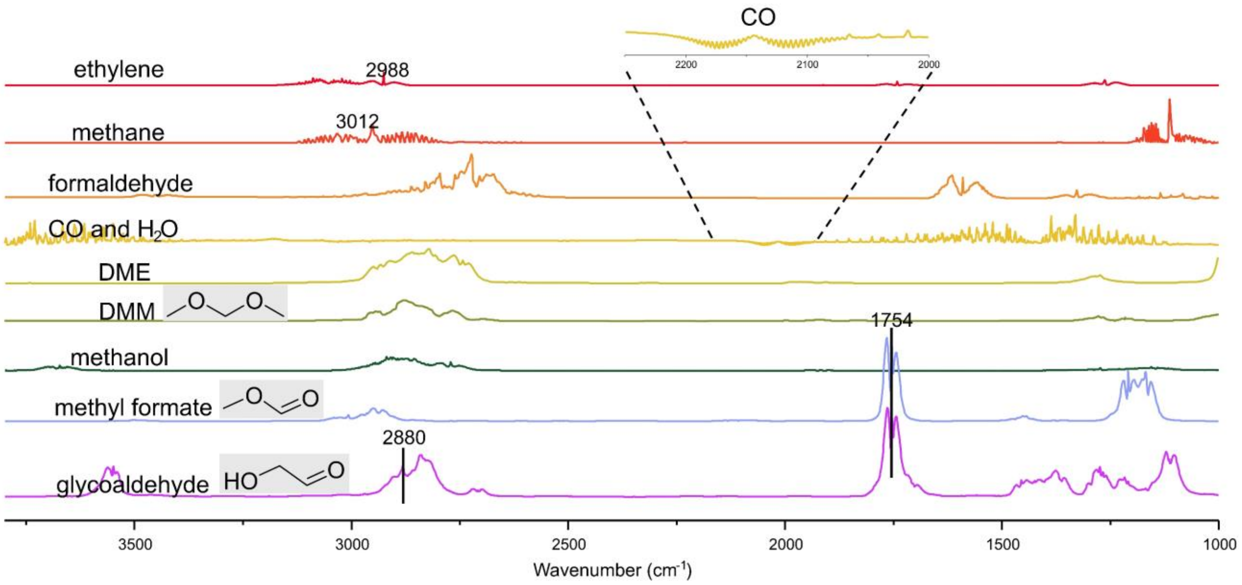

Figure S30. FT-IR spectra of related compounds in the gas phase.

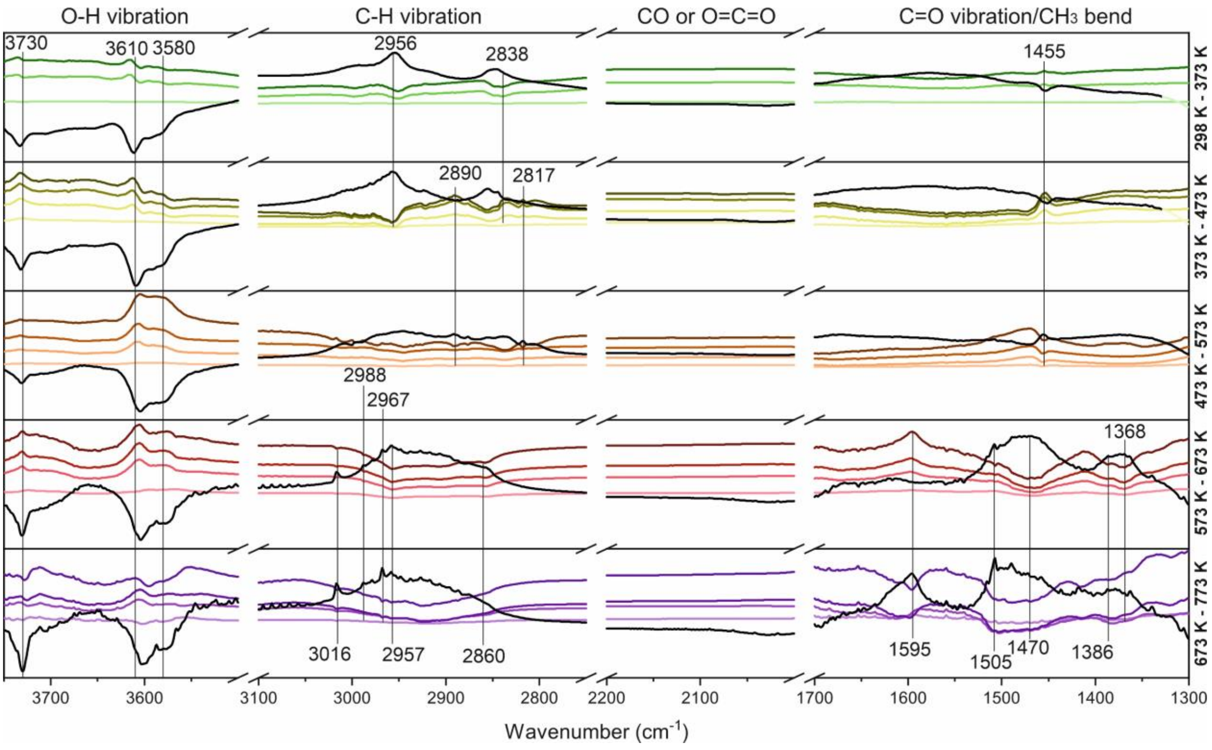

Figure S31. FT-IR spectra of CH_3OH as a reactant in H-SSZ-13 at various temperature ranges: 298 K–373 K, 373 K–473 K, 473 K–573 K, 573 K–673 K, and 673 K–773 K.

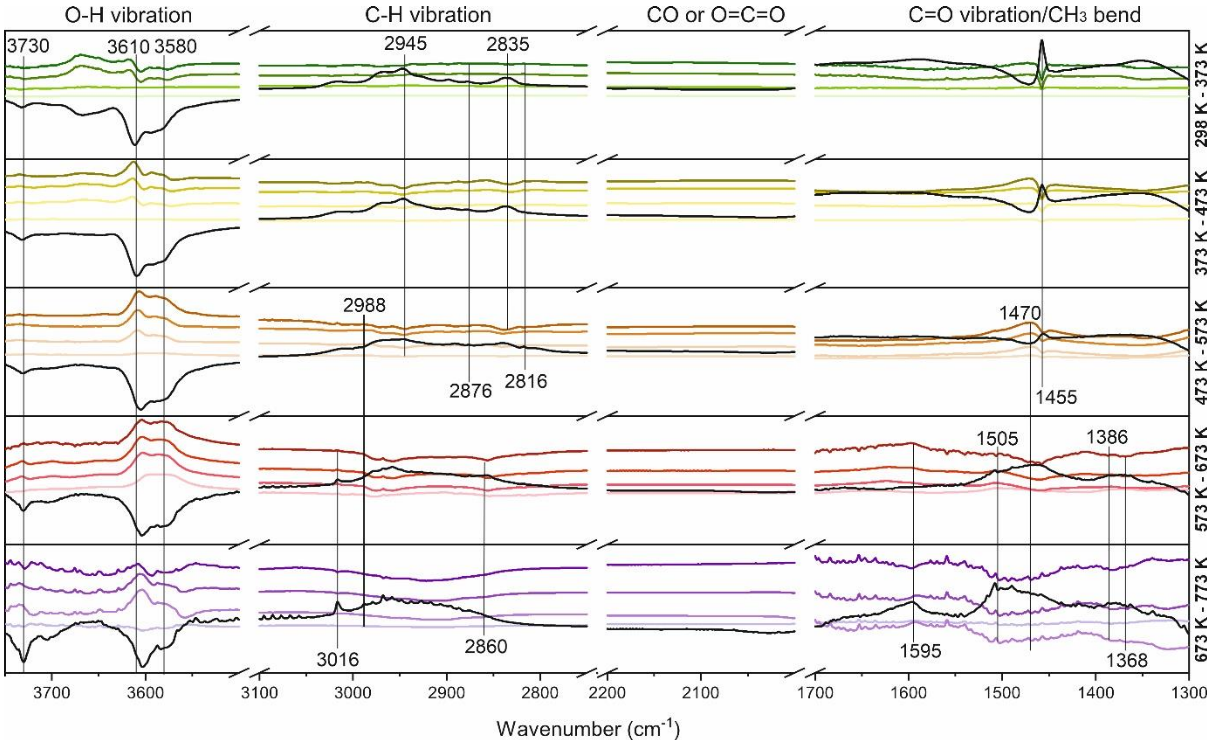

Figure S32. FT-IR spectra of DME as reactant in H-SSZ-13 at various temperature ranges: 298 K-373 K, 373 K-473 K, 473K-573K, 573K-673K, and 673K-773K.

18. MCR-ALS analysis

For the region of C-H stretching vibrations (3200 – 2600 cm^{-1}) in **Figure S33A**, it was confirmed that DME remained present for nearly the entire reaction (30 min), influencing three of the four concentration profiles. Initially, DMM is converted into HCHO and glycolaldehyde, as illustrated by the spectrum and concentration profile of species A. Concurrently, species B, recognized as methyl formate and, to a lesser degree, DME, exhibited an increase in concentration. The DME also contributes to the spectrum characteristics of species C and D. The formation of DME, associated with species C, occurred with a minor delay compared to the formation of HCHO and glycolaldehyde. In contrast, DME, represented by species D, is formed. Various formation pathways are anticipated, given that DME is engaged in multiple spectral profiles. Methanol is believed to be a source of DME in the spectrum profile of species D, whereas DME generated at the expense of DMM was observed in the spectrum profile of species C.

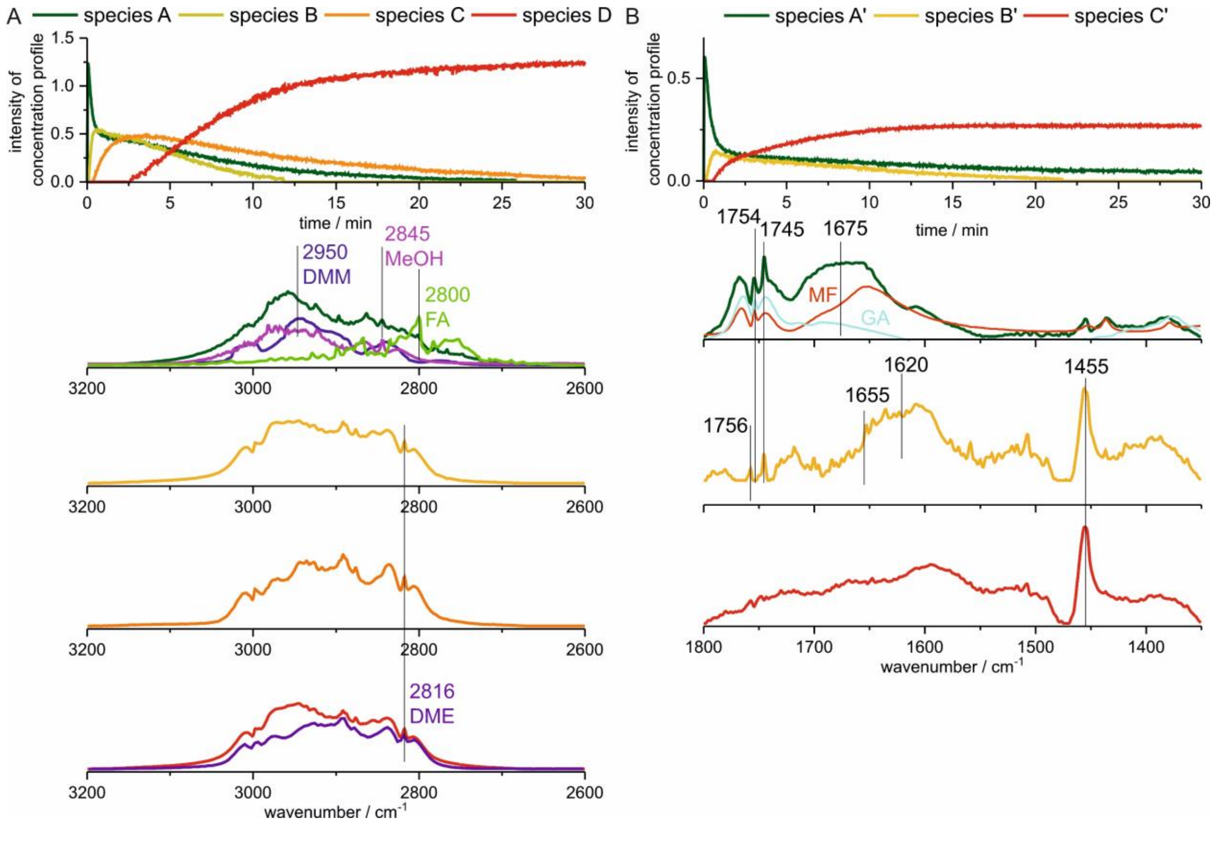

Figure S33. (A) Results of MCR analysis of *in situ* FT-IR spectra of DMM/CH_3OH=1 as a reactant in H-SSZ-13 at 493 K. (B) Spectra of selected species (MeOH, FA, DMM, GA, and MF) were added to the spectra profiles for comparison purposes.

MCR-ALS analysis in the 1800 – 1300 cm^{-1} region (**Figure S33B**) allowed us to avoid the input from the methanol and DMM substrates. Three components were then distinguished. The spectrum profile of species A' consisted of the gas phase of HCHO (1745 cm^{-1}) and glycolaldehyde (1754 cm^{-1}), with a 1675 cm^{-1} band of adsorbed glycolaldehyde. The slightly different position of the gas-phase band at 1756 cm^{-1} suggests the presence of methyl formate in the spectrum profile of species B'. In the complex band centered at 1620 cm^{-1}, adsorbed methyl formates (1655 cm^{-1}) and adsorbed water were observed. Similarly, as mentioned in the C-H stretching region analysis, DME contributes to multiple spectral profiles (species B' and C'); the DME content is predominant for species C'.

19. Time resolved FT-IR-MS analysis for methanol, DME, and DMM as reactants at 493 K and 673 K

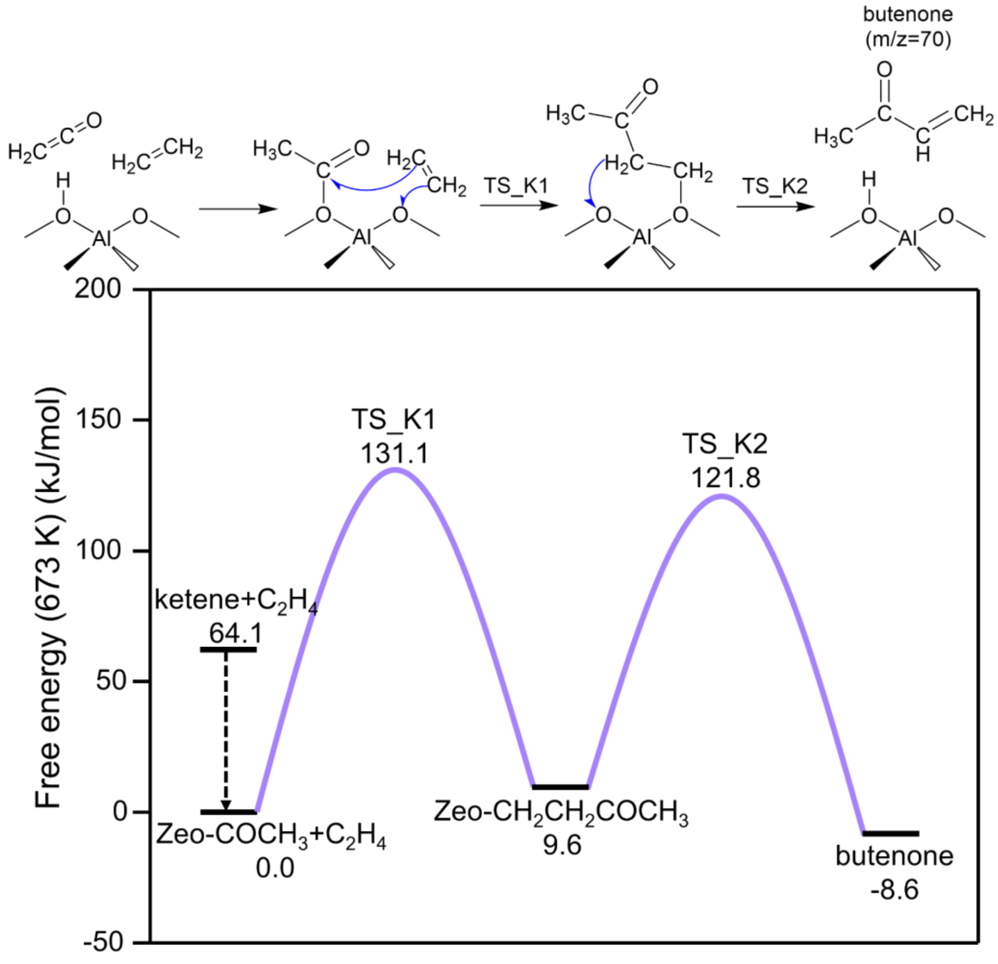

Figure S34. Reaction mechanisms and free energy surface of butenone formation from ketene and ethylene in H-SSZ-13.

We elucidated the formation of butenone and crotonaldehyde, as identified through FT-IR and MS (**Figures S36-S40),** by calculating the reaction pathway resulting from the (cyclo-)addition of ketene and ethylene. In the case of addition (**Figure S34**), ketene will be first protonated to surface acetate (Zeo-$COCH_3$), and then ethylene can couple with acetate by overcoming a barrier of 131.1 kJ/mol, finally the formed surface species (Zeo-$CH_2CH_2COCH_3$) will deprotonated to butanone ($CH_3COCHCH_2$) with a barrier of 121.8 kJ/mol. These two low barriers will allow the formation of butanone, as we observed by both FT-IR and mass spectra in **Figure S36**.

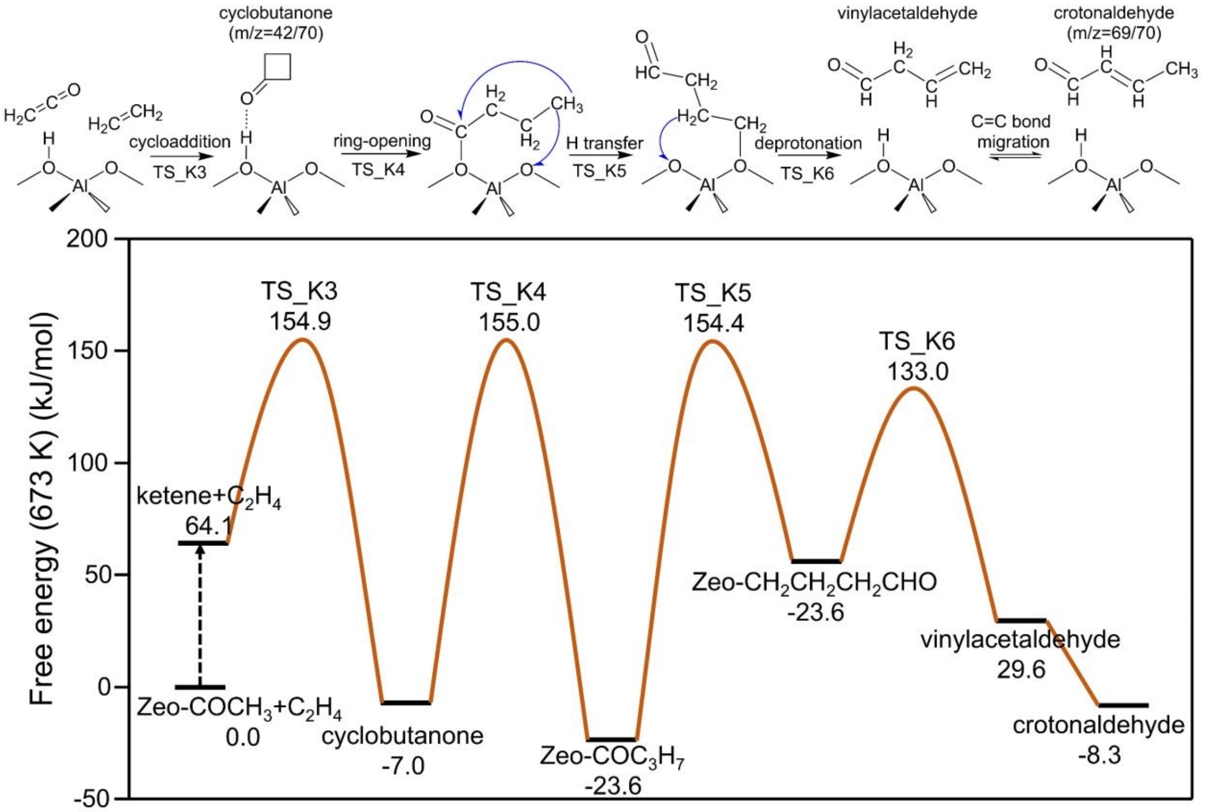

Figure S35. Reaction mechanisms and free energy surface of crotonaldehyde formation from ketene and ethylene in H-SSZ-13.

The detection of crotonaldehyde in the experiment with CH_3OH and DME as reactants (**Figures S38** and **S40**) is explained by the cycloaddition between ketene and ethylene, as displayed in **Figure S35**. The formation of crotonaldehyde will go through the cycloaddition to cyclobutanone, ring-opening of cyclobutanone to surface butyryl (Zeo-COC_3H_7), H transfer, deprotonation, and double bond migration. The

barrier of crotonaldehyde formation will be higher than that of butanone, but still possible at 673 K.

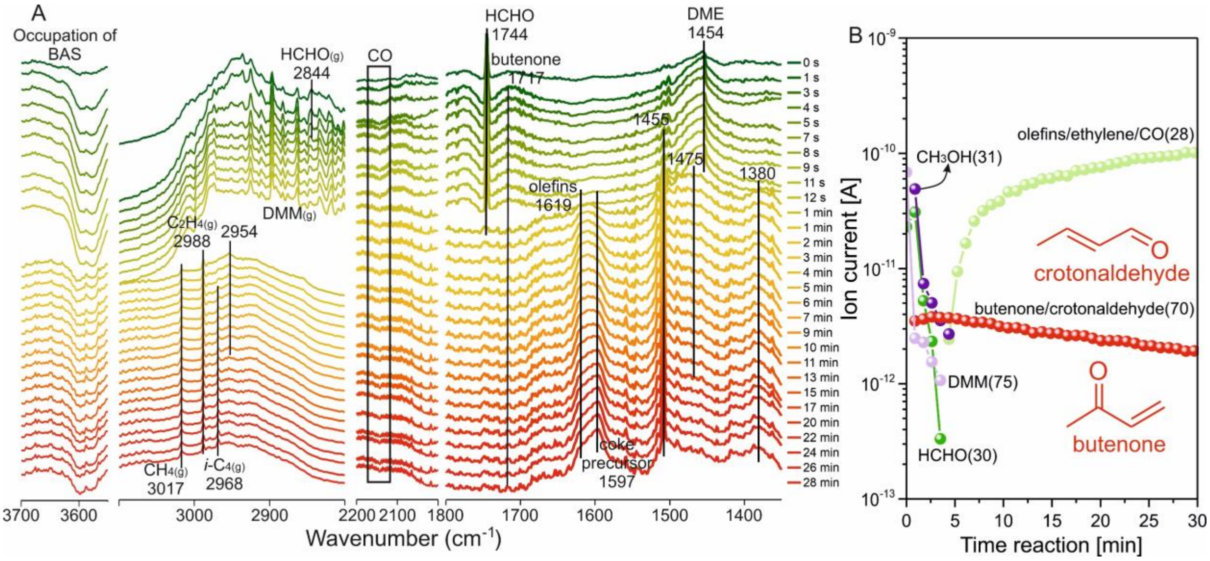

Figure S36. Time-resolved *in situ* FT-IR and mass spectra of DMM/CH_3OH=1 (2.666 kPa) as cofeeding reactants in H-SSZ-13 at 673 K. (A) FT-IR spectra, (B) mass spectra.

In the DMM/CH_3OH cofeeding experiments at 673 K (**Figure S36**), the band of glycolaldehyde was not detected because of its rapid conversion to ketene, but the band of butenone (1717 cm^{-1}) also indicates the existence of ketene and ethylene. Additionally, crotonaldehyde, as the other product of ketene and ethylene, was identified by m/z=70.

In the DMM/CH3OH cofeeding experiments at 493 K (**Figure 6**) and 673 K (**Figure S36**), two products (butenone and crotonaldehyde) for the reaction between ketene and ethylene were detected by both FT-IR and MS spectra. At 493 K, the ketene produced from the first C-C bond formation under a low conversion rate will be rapidly protonated to surface acetate by BAS, and further coupled with ethylene to produce butanone (**Figure 6B**). However, the large amount of ketene cannot be totally protonated to surface acetate at 673 K, and the cycloaddition between ketene and ethene will be possible. Then, crotonaldehyde will be formed by the cyclo-addition of ketene and ethylene to cyclobutanone and then converted into crotonaldehyde by going through the ring-opening, hydrogen transferring, and double bond migration as displayed in **Figure S35**.

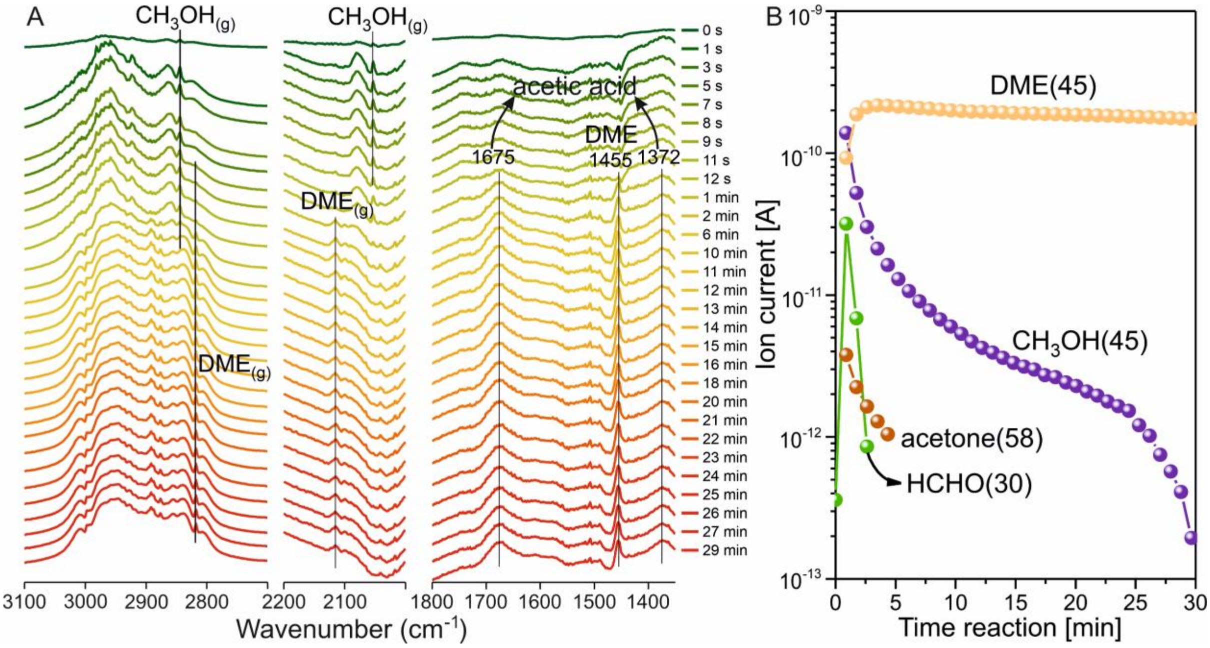

Figure S37. Time-resolved *in situ* FT-IR and mass spectra of CH_3OH (2.666 kPa) as a reactant in H-SSZ-13 zeolite at 493 K. (A) FT-IR spectra, (B) mass spectra.

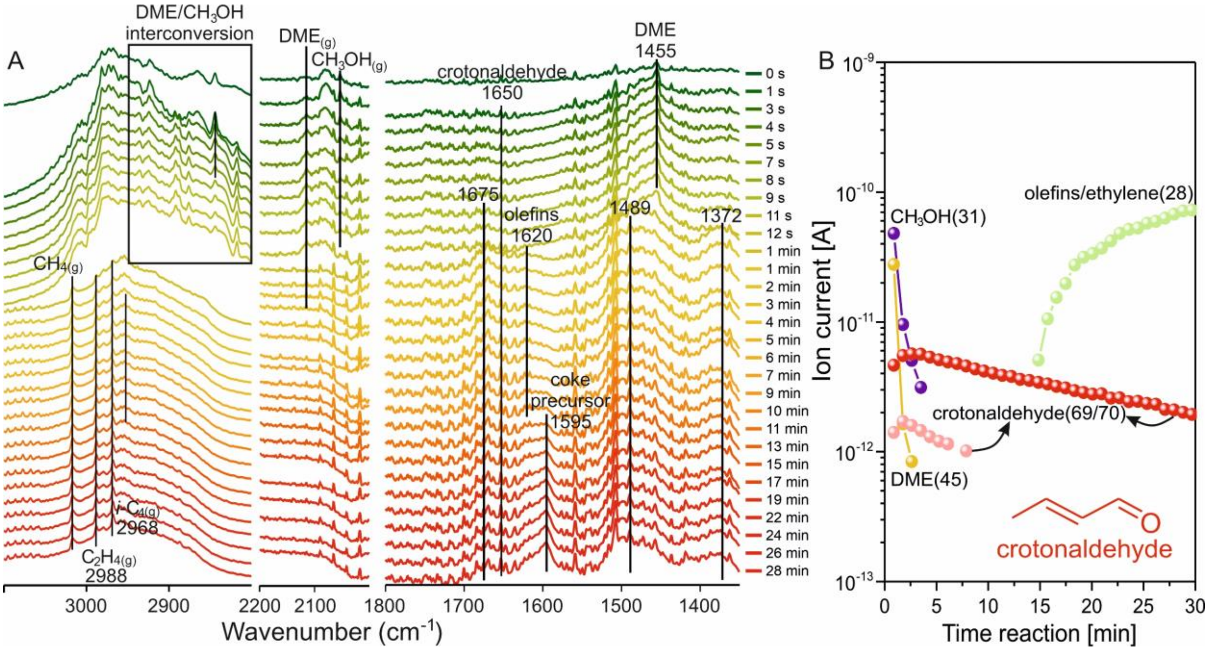

Figure S38. Time-resolved *in situ* FT-IR and mass spectra of CH_3OH (2.666 kPa) as a reactant in H-SSZ-13 at 673 K. (A) FT-IR spectra, (B) mass spectra.

In the experiment using CH_3OH as reactant at 493 K, the interconversion between CH_3OH and DME can be observed on both FT-IR and MS (**Figure S37**). Neither CO nor HCHO was detected on the IR spectrum, but some traces of m/z=30

before 3 min indicated the formation of HCHO. Then, acetone detected by m/z=58 of MS and acetic acid observed by both 1675 and 1372 cm^{-1} of FT-IR, with the consumption of HCHO, indicate the close correlation between HCHO and the first C-C bond formation in the induction period of the MTH process.

At 673 K, the MTH reaction was accelerated, and more species were detected after 1 min of DME/CH_3OH interconversion (**Figure S38**). Both 1675 and 1372 cm^{-1} of acetic acid were detected again then, but the rapid consumption of HCHO at the higher temperature lead to the no detection of HCHO by both FT-IR and MS. Almost at the same time, olefins was formed indicated by the band of 2988 cm^{-1} and 1620 cm^{-1}, both methane (3016 cm^{-1}) and isobutane (2968 cm^{-1}) in the gas phase was found on IR spectra until the end. Some coke precursors were identified by the band of 1597 cm^{-1} after 10 min of reaction.

More interestingly, two new signals of m/z=69 and 70 were detected with the same trend of evolution, probably assigned to the compound with the chemical formula of C_4H_6O. C_4H_6O can be ascribed to more than 20 compounds in the NIST chemistry book, and crotonaldehyde is the only identified compound exhibiting peaks at m/z = 69 and 70 with relative intensities comparable to those observed in our study. Moreover, the band of 1650 cm^{-1} from the beginning to the end of the reaction can be assigned to crotonaldehyde based on the reference.[27] Crotonaldehyde in zeolite can be formed by the cyclo-addition of ketene and ethylene to cyclobutanone, and then converted into crotonaldehyde by going through the ring-opening, hydrogen transferring, and double bond migration as displayed in **Figure S35**. The formation of crotonaldehyde indicates the large amount of ketene and ethylene formation at 673 K. Combining the detected HCHO at 493 K, both HCHO and ketene would play a critical role in the formation of the first C-C bond and the first olefin during the induction period of MTH process.

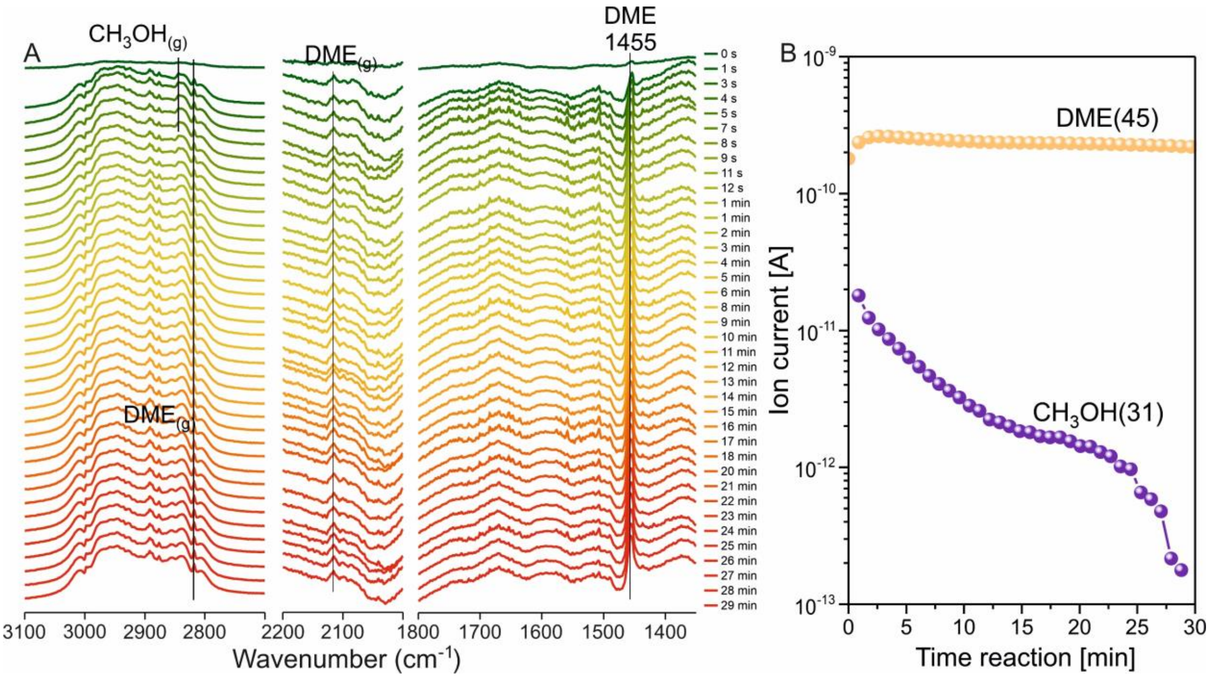

Figure S39. *In situ* time-resolved FT-IR and mass spectra of DME/CH_3OH=1 (2.666 kPa) as cofeeding reactant in H-SSZ-13 at 493 K. (A) FT-IR spectra, (B) mass spectra.

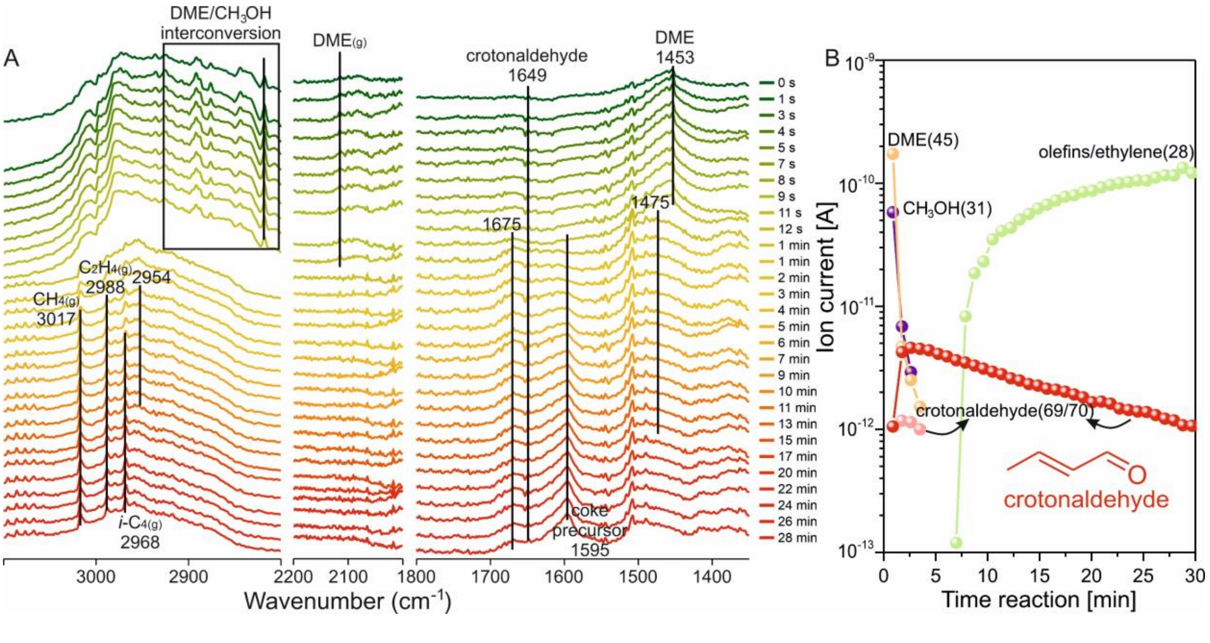

Figure S40. *In situ* time-resolved FT-IR and mass spectra of DME/CH_3OH=1 (2.666 kPa) as cofeeding reactants in H-SSZ-13 at 673 K. (A) FT-IR spectra, (B) mass spectra.

Different from CH_3OH as the sole reactant, the cofeeding of DME and CH_3OH with a ratio of 1:1 will not lead to the formation of the first C-C bond products at 493

K but will be dominated by the interconversion between DME and CH_3OH (**Figure S39**). However, like the CH_3OH feeding experiment at 673K, similar compounds were also detected by both FT-IR and mass spectrometry in **Figure S40**. Notably, crotonaldehyde was detected again by the m/z of 69 and 70, emphasizing the general applicability of the role of ketene in the induction period of the MTH process. Additionally, no detection of CO in the common MTH reaction using CH_3OH and DME as reactants (**Figures S37-S40**) may imply the weak correlation between CO and the first C-C bond formation.

20. GS analysis of gas compounds

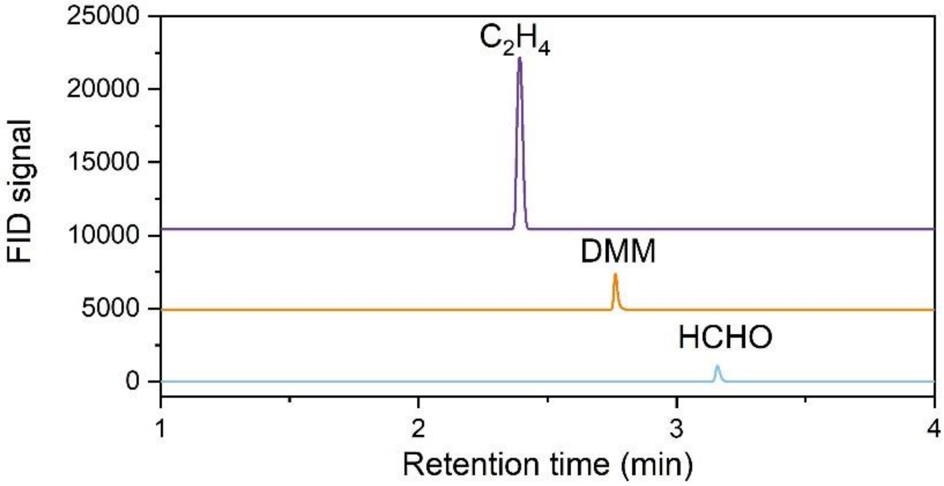

Figure S41. GC analysis of C_2H_4, DMM, and HCHO using DB-FFAP capillary column.

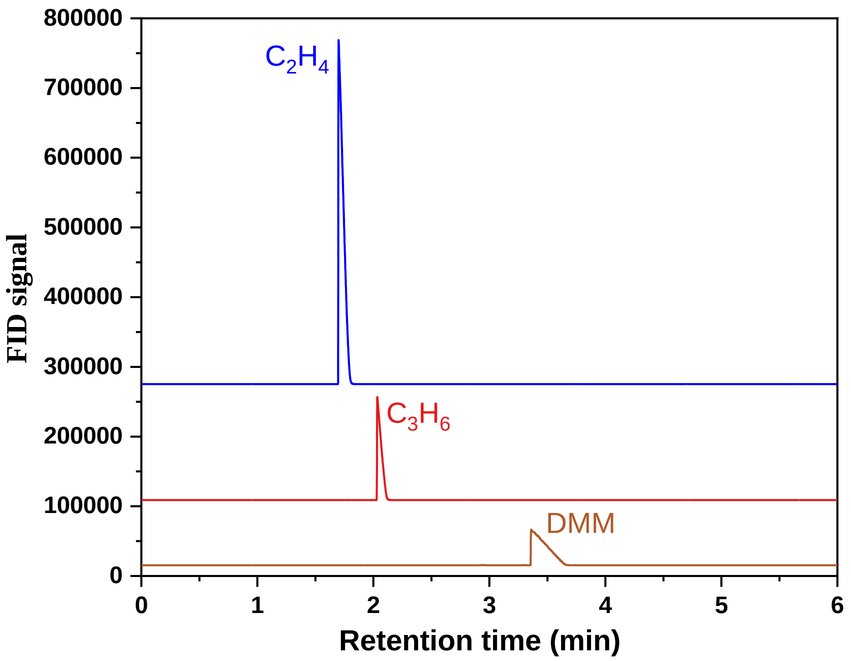

Figure S42. GC analysis of C_2H_4, C_3H_6, and DMM using PLOT-Q capillary column.

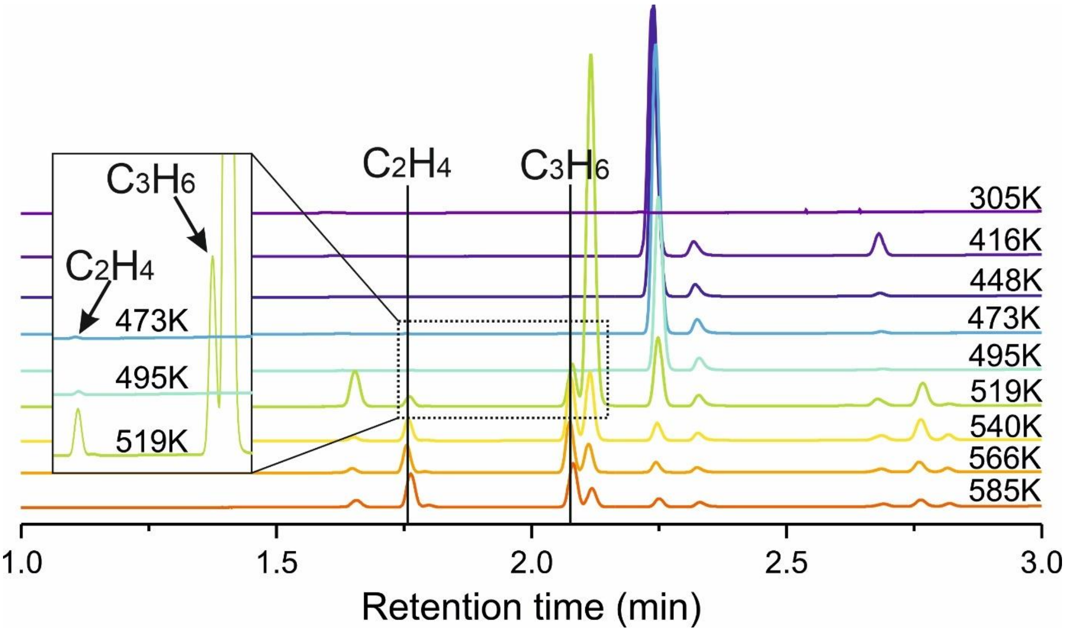

Figure S43. GC analysis of products using DMM as reactant in H-SSZ-13 (Si/Al=14.5).

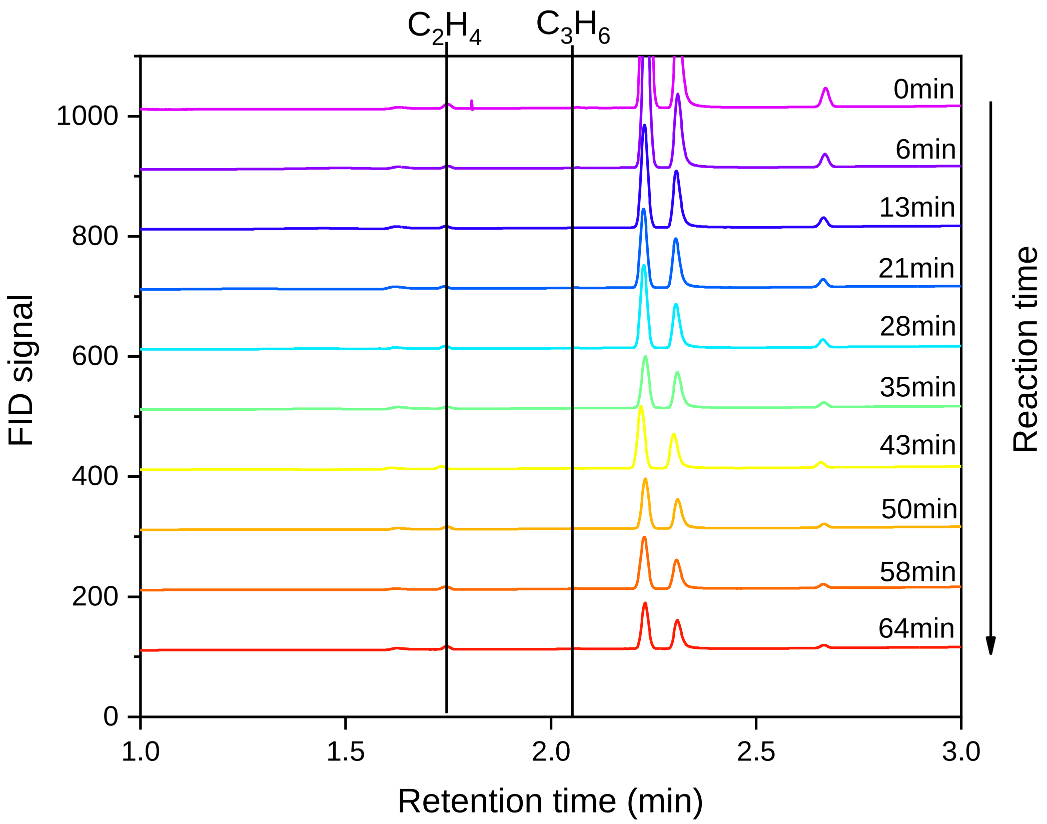

Figure S44. GC analysis of products along with reaction time using DMM as reactant in H-SSZ-13 (Si/Al=14.5) at 495 K.

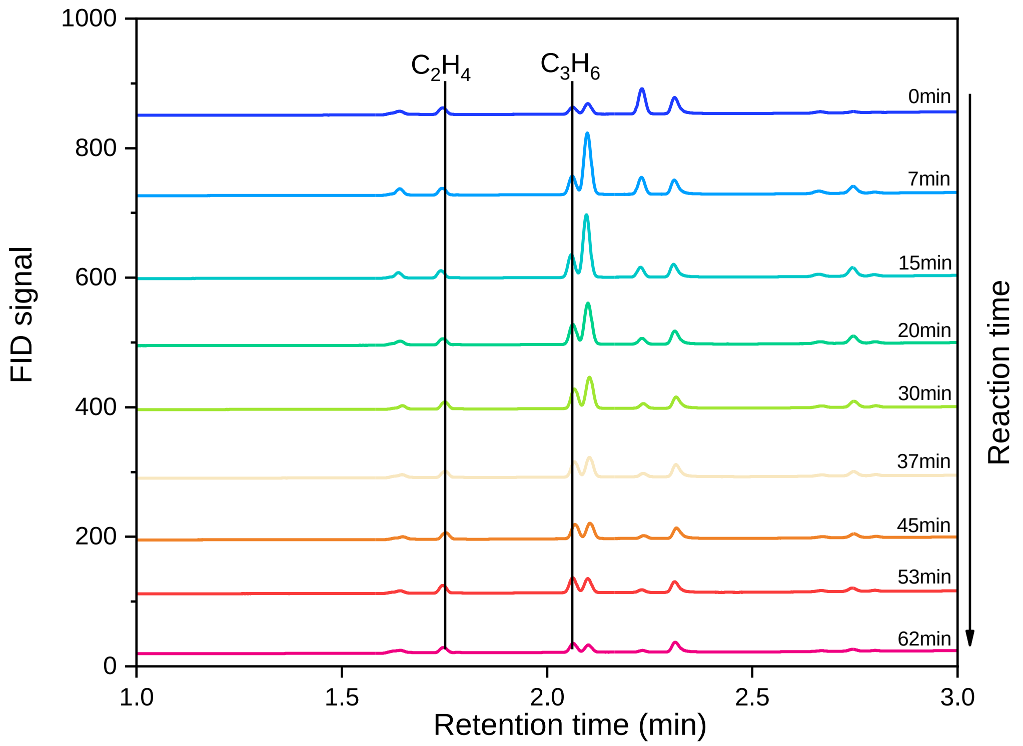

Figure S45. GC analysis of products along with reaction time using DMM as reactant in H-SSZ-13 (Si/Al=14.5) at 505 K.

21. Benchmark Study

Benchmark studies by Plessow and Studt[28-29] indicate that the PBE-D3 functional will greatly underestimate the barrier height but overestimate the adsorption energy in zeolite-catalyzed reactions, as the variant of PBE-D3, it is necessary to estimate the accuracy of revPBE-D3. Therefore, in this study, a benchmark was conducted to confirm the reliability of both barrier height and adsorption for the first C-C bond formation. There are nine different functionals in this benchmark study, including original PBE-D3 and its variants (revPBE-D3, PBEsol-D3, PBE0-D3, and RPBE-D3), BEEF-vdW as the represent of vdW-DF exchange-correlation functional, B3LYP-D3, M062X, M06 as the hybrid functionals, wB97XD as a range-separated hybrid functional with empirical dispersion corrections, post-Hartree Fock method, RPA with cc-QZVP basis set, was used as the reference method. The first C-C bond formation for CH_4, CH_3OH, and $HOCH_2OH$ to couple HCHO in SSZ-13 with SMS was employed to test, as listed in **Table S10**, and almost all DFT functionals underestimated the barrier height with a mean absolute error (MAE) of 9.4~49.5 kJ/mol, and PBEsol-D3 was excluded due to an MAE of 49.5 kJ/mol. In addition to the absolute accuracy of the barrier height by MAE, the MAE of the differences between the two barrier heights (MAE(ΔE)) is more important, which will help to determine the active order of different reactants, that is, $CH_2(OCH_3)_{2-n}(OH)_n$ (n=0, 1, 2). RPBE-D3, BEEF-vdw, B3LYP-D3, M06, and wB97XD predict a MAE($\Delta E^{\ddagger}$) larger than 10 kJ/mol. In addition to the barrier height, the adsorption energy also plays an important role in the calculation of the apparent free energy at high temperatures. Herein, the adsorption energies of $CH_2(OCH_3)_{2-n}(OH)_n$ (n=0, 1, 2) and HCHO on SSZ-13 with SMS as the adsorption process for the first C-C bond formation were calculated, as summarized in **Table S11**. Both the MAE of the adsorption energies and MAE(ΔE_{ads}) were calculated to estimate the accuracy of different functionals in the adsorption process. BEEF-vdW, B3LYP-D3, and wB97XD have an MAE of adsorption energy larger than 25 kJ/mol, but revPBE-D3, PBE-D3, and PBE0-D3 with MAE(ΔE_{ads}) smaller than 5 kJ/mol have better performance than other functionals. In consideration of both adsorption energy and barrier height, revPBE-

D3, PBE-D3, and PBE0-D3 show the low values of both MAE(ΔE_{ads}) and MAE($\Delta E^{\ddagger}$), 1.7~4.5 kJ/mol and 3.8~7.8 kJ/mol, respectively.

Table S10. Benchmark study of the energy barriers (kJ/mol) of the first C-C bond formation in SMS-SSZ-13. MAE: mean absolute error.

Methods	revPBE-D3	PBE-D3	RPBE-D3	PBEsol-D3	BEEF-vdW	B3LYP-D3	M06	M06-2X	PBE0-D3	ωB97XD	RPA
CH_4	148.8	147.3	154.5	128.1	179.1	155.8	173.3	165.2	162.9	160.3	181.4
CH_3OH	152.1	147.8	156.5	128.3	178.6	165.1	177.1	165.4	161.1	172.5	173.9
$HOCH_2OH$	118.9	118.7	130.7	91.5	162.5	122.7	115.1	120.1	126.7	160.5	141.1
MAE[a]	25.6	27.5	18.3	49.5	9.4	17.6	12.4	15.2	15.2	14.0	0.0
$\Delta_1(\Delta E^{\ddagger})$[b]	33.2	29.1	25.8	36.7	16.1	42.4	62.0	45.3	34.5	12.0	32.8
$\Delta_2(\Delta E^{\ddagger})$[c]	30.0	28.6	23.8	36.6	16.6	33.1	58.2	45.1	36.2	-0.2	40.3
$\Delta_3(\Delta E^{\ddagger})$[d]	3.2	0.4	2.0	0.1	-0.5	9.3	3.8	0.2	-1.8	12.1	-7.5
MAE($\Delta E^{\ddagger}$)[e]	7.1	7.8	11.0	5.1	15.8	11.2	19.4	8.3	3.8	27.0	0.0

[a]MAE was calculated using RPA as a reference.
[b]$\Delta_1(\Delta E^{\ddagger}) = \Delta E^{\ddagger}(CH_4) - \Delta E^{\ddagger}(CH_3OH)$
[c]$\Delta_2(\Delta E^{\ddagger}) = \Delta E^{\ddagger}(CH_4) - \Delta E^{\ddagger}(HOCH_2OH)$
[d]$\Delta_3(\Delta E^{\ddagger}) = \Delta E^{\ddagger}(CH_3OH) - \Delta E^{\ddagger}(HOCH_2OH)$
[e]MAE($\Delta E^{\ddagger}$) is the mean average error of $\Delta_1(\Delta E^{\ddagger})$, $\Delta_2(\Delta E^{\ddagger})$, and $\Delta_3(\Delta E^{\ddagger})$, using RPA as a reference.

Table S11. Benchmark studies of the adsorption energies (kJ/mol) of HCHO and CH_4/CH_3OH/HOCH2OH on SMS-SSZ-13. MAE: mean absolute error.

Methods	revPBE-D3	PBE-D3	RPBE-D3	PBEsol-D3	BEEF-vdW	B3LYP-D3	M06	M06-2X	PBE0-D3	ωB97XD	RPA
CH_4	-88.0	-85.6	-88.7	-79.7	-96.4	-93.5	-77.6	-69.7	-88.1	-40.0	-74.7
CH_3OH	-101.5	-100.9	-103.5	-91.7	-118.3	-100.9	-80.5	-74.8	-101.8	-52.4	-87.7
$HOCH_2OH$	-116.7	-116.9	-115.7	-112.9	-131.6	-142.5	-85.6	-99.0	-115.3	-72.4	-99.2
MAE[a]	14.8	13.9	15.4	7.6	28.2	25.1	7.9	6.0	14.5	32.3	0.0
$\Delta_1(\Delta E_{abs})$[b]	13.5	15.3	14.8	12.0	21.9	7.3	2.9	5.0	13.6	12.4	13.0
$\Delta_2(\Delta E_{abs})$[c]	28.7	31.3	27.0	33.2	35.2	48.9	8.0	29.3	27.1	32.4	24.5
$\Delta_3(\Delta E_{abs})$[d]	15.2	16.0	12.2	21.2	13.4	41.6	5.1	24.3	13.5	20.0	11.5
MAE(ΔE_{abs})[e]	2.8	4.5	1.7	6.4	7.1	20.0	11.0	8.5	1.7	5.6	0.0

[a]MAE was calculated using RPA as a reference.
[b]$\Delta_1(\Delta E_{ads}) = \Delta E_{ads}(CH_4) - \Delta E_{ads}(CH_3OH)$
[c]$\Delta_2(\Delta E_{ads}) = \Delta E_{ads}(CH_4) - \Delta E_{ads}(HOCH_2OH)$
[d]$\Delta_3(\Delta E_{ads}) = \Delta E_{ads}(CH_3OH) - \Delta E_{ads}(HOCH_2OH)$
[e]MAE(ΔE_{ads}) is the mean average error of $\Delta_1(\Delta E_{ads})$, $\Delta_2(\Delta E_{ads})$, and $\Delta_3(\Delta E_{ads})$, using RPA as a reference.

We further compare the potential deviation between Gaussian and Plane Waves (GPW) in CP2K and Projector Augmented Wave (PAW) method in VASP.[30] The free energy surface of SMS + HCHO + $CH_3OCH_2OCH_3$ to P6 obtained by the same revPBE-D3 functional in two software were calculated and displayed in **Figure S46**. During the geometrical optimization of both the transition state and minima, the cutoff

energy was set to 550 eV, and the convergence criteria of electrons and atoms were 10^{-7} eV and 0.01 eV/atom, respectively. VASPKIT software[31] was used to calculate the Gibbs free energy correction at 673 K based on the frequency calculation at the same level. The free-energy surfaces obtained by CP2K and VASP are quite close, and the difference in the free-energy barrier is only 2 kJ/mol. Moreover, the consistency of CP2K and VASP is also reflected in the prediction of the free energy of Prod. and reversed free energy barriers (Prod. to Int1) are 184.9 kJ/mol by CP2K and 186.0 kJ/mol by VASP. Based on these comparisons, the revPBE-D3/TZVP//CP2K strategy had the same accuracy as the regular revPBE-D3/PAW//VASP strategy.

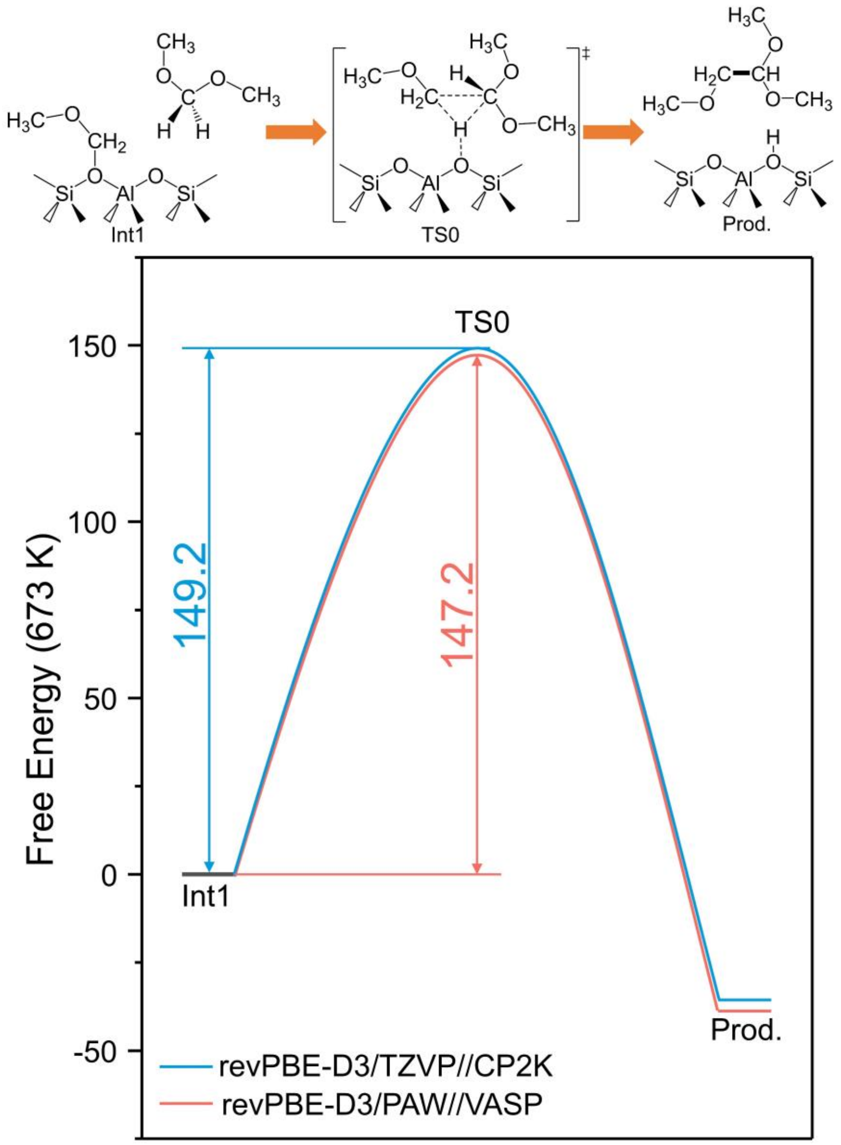

Figure S46. Comparison between VASP and CP2K for the free energy surface of SMS + HCHO + $CH_3OCH_2OCH_3$ to P6 in SSZ-13 zeolite at 673 K.

References

1. Hutter, J.; Iannuzzi, M.; Schiffmann, F.; VandeVondele, J., CP2K: atomistic simulations of condensed matter systems. *Wires Comput Mol Sci* **2014,** *4* (1), 15-25.
2. Perdew, J. P.; Burke, K.; Ernzerhof, M., Generalized gradient approximation made simple. *Phys Rev Lett* **1996,** *77* (18), 3865-3868.
3. Zhang, Y. K.; Yang, W. T., Comment on "Generalized gradient approximation made simple". *Phys Rev Lett* **1998,** *80* (4), 890-890.
4. Grimme, S.; Antony, J.; Ehrlich, S.; Krieg, H., A consistent and accurate ab initio parametrization of density functional dispersion correction (DFT-D) for the 94 elements H-Pu. *J Chem Phys* **2010,** *132* (15), 154104.
5. Lippert, G.; Hutter, J.; Parrinello, M., The Gaussian and augmented-plane-wave density functional method for ab initio molecular dynamics simulations. *Theor Chem Acc* **1999,** *103* (2), 124-140.
6. Goedecker, S.; Teter, M.; Hutter, J., Separable dual-space Gaussian pseudopotentials. *Phys Rev B* **1996,** *54* (3), 1703-1710.
7. Sun, T. T.; Chen, W.; Xu, S. T.; Zheng, A. M.; Wu, X. Q.; Zeng, S.; Wang, N.; Meng, X. J.; Wei, Y. X.; Liu, Z. M., The first carbon-carbon bond formation mechanism in methanol-to-hydrocarbons process over chabazite zeolite. *Chem* **2021,** *7* (9), 2415-2428.
8. Brogaard, R. Y.; Henry, R.; Schuurman, Y.; Medford, A. J.; Moses, P. G.; Beato, P.; Svelle, S.; Norskov, J. K.; Olsbye, U., Methanol-to-hydrocarbons conversion: The alkene methylation pathway. *J Catal* **2014,** *314*, 159-169.
9. Brogaard, R. Y.; Wang, C.-M.; Studt, F., Methanol–Alkene Reactions in Zeotype Acid Catalysts: Insights from a Descriptor-Based Approach and Microkinetic Modeling. *ACS Catal* **2014,** *4* (12), 4504-4509.
10. Lu, T.; Chen, Q. X., Shermo: A general code for calculating molecular thermochemistry properties. *Comput Theor Chem* **2021,** *1200*.
11. Bonomi, M.; Bussi, G.; Camilloni, C.; Tribello, G. A.; Banas, P.; Barducci, A.; Bernetti, M.; Bolhuis, P. G.; Bottaro, S.; Branduardi, D.; Capelli, R.; Carloni, P.; Ceriotti, M.; Cesari, A.; Chen, H. C.; Chen, W.; Colizzi, F.; De, S.; De La Pierre, M.; Donadio, D.; Drobot, V.; Ensing, B.; Ferguson, A. L.; Filizola, M.; Fraser, J. S.; Fu, H. H.; Gasparotto, P.; Gervasio, F. L.; Giberti, F.; Gil-Ley, A.; Giorgino, T.; Heller, G. T.; Hocky, G. M.; Iannuzzi, M.; Invernizzi, M.; Jelfs, K. E.; Jussupow, A.; Kirilin, E.; Laio, A.; Limongelli, V.; Lindorff-Larsen, K.; Lohr, T.; Marinelli, F.; Martin-Samos, L.; Masetti, M.; Meyer, R.; Michaelides, A.; Molteni, C.; Morishita, T.; Nava, M.; Paissoni, C.; Papaleo, E.; Parrinello, M.; Pfaendtner, J.; Piaggi, P.; Piccini, G.; Pietropaolo, A.; Pietrucci, F.; Pipolo, S.; Provasi, D.; Quigley, D.; Raiteri, P.; Raniolo, S.; Rydzewski, J.; Salvalaglio, M.; Sosso, G. C.; Spiwok, V.; Sponer, J.; Swenson, D. W. H.; Tiwary, P.; Valsson, O.; Vendruscolo, M.; Voth, G. A.; White, A., Promoting transparency and reproducibility in enhanced molecular simulations. *Nat Methods* **2019,** *16* (8), 670-673.
12. Posch, H. A.; Hoover, W. G.; Vesely, F. J., Canonical dynamics of the nose oscillator - stability, order, and chaos. *Phys Rev A* **1986,** *33* (6), 4253-4265.
13. Jin, Y.; Sun, Q.; Qi, G.; Yang, C.; Xu, J.; Chen, F.; Meng, X.; Deng, F.; Xiao, F. S., Solvent-free synthesis of silicoaluminophosphate zeolites. *Angewandte Chemie* **2013,** *125* (35), 9172-9175.
14. Gao, F.; Washton, N. M.; Wang, Y.; Kollár, M.; Szanyi, J.; Peden, C. H., Effects of Si/Al ratio on Cu/SSZ-13 NH3-SCR catalysts: Implications for the active Cu species and the roles of Brønsted acidity. *J Catal* **2015,** *331*, 25-38.
15. Molokova, A. Y.; Abasabadi, R. K.; Borfecchia, E.; Mathon, O.; Bordiga, S.; Wen, F.; Berlier, G.; Janssens, T. V. W.; Lomachenko, K. A., Elucidating the reaction mechanism of SO2 with Cu-CHA catalysts for NH3-SCR by X-ray absorption spectroscopy. *Chem Sci* **2023,** *14* (41), 11521-11531.
16. de Juan, A.; Jaumot, J.; Tauler, R., Multivariate Curve Resolution (MCR). Solving the mixture analysis problem. *Analytical Methods* **2014,** *6* (14), 4964-4976.

17. Jaumot, J.; de Juan, A.; Tauler, R., MCR-ALS GUI 2.0: New features and applications. *Chemometrics and Intelligent Laboratory Systems* **2015,** *140*, 1-12.
18. Jaumot, J.; Gargallo, R.; de Juan, A.; Tauler, R., A graphical user-friendly interface for MCR-ALS: a new tool for multivariate curve resolution in MATLAB. *Chemometrics and Intelligent Laboratory Systems* **2005,** *76* (1), 101-110.
19. Frisch, M. J.; Trucks, G. W.; Schlegel, H. B.; Scuseria, G. E.; Robb, M. A.; Cheeseman, J. R.; Scalmani, G.; Barone, V.; Petersson, G. A.; Nakatsuji, H.; Li, X.; Caricato, M.; Marenich, A. V.; Bloino, J.; Janesko, B. G.; Gomperts, R.; Mennucci, B.; Hratchian, H. P.; Ortiz, J. V.; Izmaylov, A. F.; Sonnenberg, J. L.; Williams; Ding, F.; Lipparini, F.; Egidi, F.; Goings, J.; Peng, B.; Petrone, A.; Henderson, T.; Ranasinghe, D.; Zakrzewski, V. G.; Gao, J.; Rega, N.; Zheng, G.; Liang, W.; Hada, M.; Ehara, M.; Toyota, K.; Fukuda, R.; Hasegawa, J.; Ishida, M.; Nakajima, T.; Honda, Y.; Kitao, O.; Nakai, H.; Vreven, T.; Throssell, K.; Montgomery Jr., J. A.; Peralta, J. E.; Ogliaro, F.; Bearpark, M. J.; Heyd, J. J.; Brothers, E. N.; Kudin, K. N.; Staroverov, V. N.; Keith, T. A.; Kobayashi, R.; Normand, J.; Raghavachari, K.; Rendell, A. P.; Burant, J. C.; Iyengar, S. S.; Tomasi, J.; Cossi, M.; Millam, J. M.; Klene, M.; Adamo, C.; Cammi, R.; Ochterski, J. W.; Martin, R. L.; Morokuma, K.; Farkas, O.; Foresman, J. B.; Fox, D. J. *Gaussian 16 Rev. C.01*, Wallingford, CT, 2016.
20. Tajima, N.; Tsuneda, T.; Toyama, F.; Hirao, K., A new mechanism for the first carbon-carbon bond formation in the MTG process: A theoretical study. *J Am Chem Soc* **1998,** *120* (32), 8222-8229.
21. Lesthaeghe, D.; Van Speybroeck, V.; Marin, G. B.; Waroquier, M., Understanding the failure of direct C-C coupling in the zeolite-catalyzed methanol-to-olefin process. *Angew Chem Int Ed* **2006,** *45* (11), 1714-1719.
22. Wei, Z. H.; Chen, Y. Y.; Li, J. F.; Guo, W. P.; Wang, S.; Dong, M.; Qin, Z. F.; Wang, J. G.; Jiao, H. J.; Fan, W. B., Stability and reactivity of intermediates of methanol related reactions and C-C bond formation over H-ZSM-5 acidic catalyst: A computational analysis. *J Phys Chem C* **2016,** *120* (11), 6075-6087.
23. Chu, Y.; Yi, X.; Li, C.; Sun, X.; Zheng, A., Brønsted/Lewis acid sites synergistically promote the initial C–C bond formation in the MTO reaction. *Chem Sci* **2018,** *9* (31), 6470-6479.
24. Li, J. F.; Wei, Z. H.; Chen, Y. Y.; Jing, B. Q.; He, Y.; Dong, M.; Jiao, H. J.; Li, X. K.; Qin, Z. F.; Wang, J. G.; Fan, W. B., A route to form initial hydrocarbon pool species in methanol conversion to olefins over zeolites. *J Catal* **2014,** *317*, 277-283.
25. Khare, R.; Arora, S. S.; Bhan, A., Implications of Cofeeding Acetaldehyde on Ethene Selectivity in Methanol-to-Hydrocarbons Conversion on MFI and Its Mechanistic Interpretation. *ACS Catal* **2016,** *6* (4), 2314-2331.
26. Huber, P.; Plessow, P. N., A computational investigation of the decomposition of acetic acid in H-SSZ-13 and its role in the initiation of the MTO process. *Catal Sci Technol* **2023,** *13* (6), 1905-1917.
27. Lindenmaier, R.; Williams, S. D.; Sams, R. L.; Johnson, T. J., Quantitative Infrared Absorption Spectra and Vibrational Assignments of Crotonaldehyde and Methyl Vinyl Ketone Using Gas-Phase Mid-Infrared, Far-Infrared, and Liquid Raman Spectra: s-cis vs s-trans Composition Confirmed via Temperature Studies and ab Initio Methods. *The Journal of Physical Chemistry A* **2017,** *121* (6), 1195-1212.
28. Goncalves, T. J.; Plessow, P. N.; Studt, F., On the accuracy of density functional theory in zeolite catalysis. *ChemCatChem* **2019,** *11* (17), 4368-4376.
29. Plessow, P. N.; Studt, F., How Accurately Do Approximate Density Functionals Predict Trends in Acidic Zeolite Catalysis? *J Phys Chem Lett* **2020,** *11* (11), 4305-4310.
30. Monkhorst, H. J.; Pack, J. D., Special Points for Brillouin-Zone Integrations. *Phys Rev B* **1976,** *13* (12), 5188-5192.
31. Wang, V.; Xu, N.; Liu, J. C.; Tang, G.; Geng, W. T., VASPKIT: A user-friendly interface facilitating high-throughput computing and analysis using VASP code. *Comput Phys Commun* **2021,** *267*, 108033.
